# Identification of methylation-sensitive human transcription factors using meSMiLE-seq

**DOI:** 10.1038/s41467-026-71387-y

**Published:** 2026-08-05

**Authors:** Antoni J. Gralak, Katerina Faltejskova, Ally W. H. Yang, Clemence Steiner, Julie Russeil, Nadia Grenningloh, Sachi Inukai, Mustafa Demir, Riccardo Dainese, Cooper Owen, Eugenia V. Pankevich, Codebook/GRECO-BIT Consortium, Antoni J. Gralak, Katerina Faltejskova, Ally W. H. Yang, Sachi Inukai, Philipp Bucher, Oriol Fornes, Jan Grau, Ivo Grosse, Arttu Jolma, Fedor A. Kolpakov, Vsevolod J. Makeev, Mihai Albu, Marjan Barazandeh, Alexander Brechalov, Zhenfeng Deng, Ali Fathi, Chun Hu, Samuel A. Lambert, Kaitlin U. Laverty, Zain M. Patel, Sara E. Pour, Rozita Razavi, Mikhail Salnikov, Isaac Yellan, Hong Zheng, Georgy Meshcheryakov, Giovanna Ambrosini, Marie-Luise Plescher, Semyon Kolmykov, Ivan Yevshin, Nikita Gryzunov, Ivan Kozin, Mikhail Nikonov, Vladimir Nozdrin, Arsenii Zinkevich, Pavel Kravchenko, Sergey Abramov, Alexandr Boytsov, Vasilii Kamenets, Dmitry Penzar, Anton Vlasov, Ilya E. Vorontsov, Aldo Hernandez-Corchado, Hamed S. Najafabadi, Quaid Morris, Xiaoting Chen, Matthew T. Weirauch, Timothy R. Hughes, Ivan V. Kulakovskiy, Judith F. Kribelbauer-Swietek, Bart Deplancke, Timothy R. Hughes, Ivan V. Kulakovskiy, Judith F. Kribelbauer-Swietek, Guido van Mierlo, Bart Deplancke

**Affiliations:** 1 Laboratory of Systems Biology and Genetics, Institute of Bioengineering, School of Life Sciences, Ecole Polytechnique Fédérale de Lausanne (EPFL), Lausanne, Switzerland; 2 Swiss Institute of Bioinformatics, Lausanne, Switzerland; 3 Institute of Organic Chemistry and Biochemistry, Czech Academy of Sciences, Prague, Czechia; 4 Computer Science Institute, Faculty of Mathematics and Physics, Charles University, Prague, Czechia; 5 Donnelly Centre and Department of Molecular Genetics, University of Toronto, Toronto, ON Canada; 6 Institute of Protein Research, Russian Academy of Sciences, Pushchino, Russia; 7 Vavilov Institute of General Genetics, Russian Academy of Sciences, Moscow, Russia; 8 Institute of Biochemistry and Genetics, Ufa Federal Research Centre of Russian Academy of Sciences, Ufa, Russia; 9 Department of Biological Sciences, University of Southern California, Los Angeles, CA USA; 10 Department of Medical BioSciences, Radboud University Medical Center, Nijmegen, The Netherlands; 11 Present Address: Chugai Pharmaceutical Co. Ltd, Tokyo, Japan; 12 Present Address: Alithea Genomics SA, Epalinges, Switzerland; 13 Department of Medical Genetics, Centre for Molecular Medicine and Therapeutics, BC Children’s Hospital Research Institute, University of British Columbia, Vancouver, BC Canada; 14 Institute of Computer Science, Martin Luther University Halle-Wittenberg, Halle, Germany; 15 Department of Computational Biology, Sirius University of Science and Technology, Sirius, Russia; 16 Bioinformatics Laboratory, Federal Research Center for Information and Computational Technologies, Novosibirsk, Russia; 17 Moscow Center for Advanced Studies, Moscow, Russia; 18 Bioinformatics Competence Center, Ecole Polytechnique Fédérale de Lausanne, Lausanne, Switzerland; 19 Bioinformatics Competence Center, Université de Lausanne, Lausanne, Switzerland; 20 Biosoft.Ru LLC, Novosibirsk, Russia; 21 Life Improvement by Future Technologies (LIFT) Center, Moscow, Russia; 22 Faculty of Bioengineering and Bioinformatics, Lomonosov Moscow State University, Moscow, Russia; 23 Max Planck Institute of Biochemistry, Planegg, Germany; 24 Altius Institute for Biomedical Sciences, Seattle, WA USA; 25 TUD Dresden University of Technology, Center for Molecular and Cellular Bioengineering (CMCB), Biotechnologisches Zentrum (BIOTEC), Dresden, Germany; 26 McGill University and Génome Québec Innovation Centre, Montréal, QC Canada; 27 Memorial Sloan Kettering Cancer Center, Rockefeller Research Laboratories, New York, NY USA; 28 Cincinnati Children’s Hospital, Cincinnati, OH USA; 29 Present Address: Cancer Research UK National Biomarker Centre, University of Manchester, Manchester, UK

**Keywords:** DNA, Methylation analysis, DNA methylation, Transcription, DNA methylation

## Abstract

Transcription factors (TFs) are key players in eukaryotic gene regulation, but the DNA binding specificity of many TFs remains unknown. Here, we assay 284 mostly uncharacterized putative human TFs using selective microfluidics-based ligand enrichment followed by sequencing (SMiLE-seq), revealing 74 new DNA binding motifs. To investigate whether TFs lacking detectable motifs preferably bind epigenetically modified DNA, we develop methylation-sensitive SMiLE-seq (meSMiLE-seq), a microfluidic assay that simultaneously probes binding to methylated and unmethylated DNA. Using meSMiLE-seq, we assay 114 TFs and identify DNA-binding models for 48 proteins, including known methylation-sensitive binding modes for POU5F1 and RFX5. 11 TFs prefer methylated DNA or display alternative methylation-dependent motifs (e.g. PRDM13), while 13 show aversion to methylated sequences (e.g. USF3). Finally, we identify ZHX2 as a putative Z-DNA binder. Altogether, our study significantly expands the human TF codebook, while providing a versatile platform to quantitatively assay the impact of DNA modifications on TF binding.

## Abstract (web summary)

As part of the Codebook consortium, this study profiles 284 largely uncharacterized human transcription factors with a microfluidic methylation-sensitive DNA-binding assay, uncovering 74 new motifs and showing how DNA methylation shapes TF binding.

## Introduction

Transcription factors (TFs) are critical for gene regulation by binding their cognate binding sites (TFBS) to modulate target gene expression^1^. The interaction between TFs and DNA tends to be mediated through DNA-binding domains (DBDs) that recognize distinct DNA patterns (also called “DNA motifs”). Despite their crucial role in gene regulation, the TFBS for approximately 400 out of around 1600 putative human TFs remain poorly characterized^1–3^. The largest and most diverse TF family is the Cys_2_His_2_ zinc finger proteins (C2H2 ZNFs), which constitute the majority of uncharacterized TFs. A human ZNF contains, on average, 11 individual zinc finger domains (ZFDs), most of which are thought to be capable of binding DNA^1,4^. However, not all ZFDs necessarily make DNA contacts, while others have noncanonical effects, which complicates the inference of binding properties, resulting in nearly one third of all ~750 predicted ZNFs still lacking clearly defined binding motifs^1,5^. Additionally, recent studies have provided evidence that DNA binding is not exclusively mediated by structured DBDs and that intrinsically disordered regions (IDRs) can also alter a TF’s sequence specificity and even affinity to DNA^6–8^. Given the difficulty in structurally modeling such IDRs, this also renders the in silico prediction of DNA binding motifs challenging despite major recent computational advances^4,9^. The experimental identification of protein-DNA interactions, therefore, remains an important outstanding challenge, as it continues to be seen as the gold standard to derive DNA binding motifs.

Another convoluting factor for defining TF binding models is the epigenetic state of DNA, specifically the methylation of CG dinucleotides, which can drastically alter the binding affinity of TFs to their respective binding sites^10,11^. For instance, TF families including bHLH and bZIP TFs are frequently repelled by CG methylation^12^, while MBDs or C/EBP have an increased affinity for methylated CGs in a specific sequence context^13,14^. Various in vitro methods based on protein binding microarrays (PBMs) and systematic evolution of ligands by exponential enrichment (SELEX) have been developed to define the effect of DNA modification on TF binding in high-throughput, and have revealed that CG methylation can differentially modulate binding affinities even for TFs in the same TF family^12,15,16^. These methods enable profiling TF interactions with methylated DNA, but they also retain the disadvantages of the original techniques, such as the limited combinatorial space in PBM and the surplus enrichment of the strongest binding sites in SELEX. While information about methylation specificity can to some extent also be imputed from genomic assays such as chromatin immunoprecipitation sequencing (ChIP-seq), these approaches typically lack resolution (fragment sizes generally span hundreds of base pairs) and require an indirect link between TF-associated peaks with in-depth whole genome bisulfite sequencing (WGBS) from the matched cell types, as the methylation status of pulled-down, TF-bound sequences is often not available. Additionally, ChIP-seq-derived binding motifs might be cell-type- or co-factor-specific and do not necessarily reflect a TF’s ability to directly bind or evade epigenetically modified DNA.

To assign binding motifs for the remaining, uncharacterized human TFs, the Codebook and GRECO-BIT initiatives joined in a collaborative attempt to determine the DNA binding sites for the remaining putative DNA-binding TFs^17^. In this effort, a total of 393 TFs (332 putative TFs, and 61 control TFs) were assayed using five orthogonal experimental DNA binding assays. The latter included ChIP-seq^18^, genomic and classical high-throughput SELEX ((G)HT-SELEX)^19^, and PBMs^17^. A fifth method is presented here: selective microfluidics-based ligand enrichment followed by sequencing (SMiLE-seq), which is a high-throughput microfluidic technique for examining TF-DNA interactions that relies on the mechanically induced trapping of molecular interactions (MITOMI)^20^ concept. This comprehensive experimental strategy aimed to leverage the strengths of each of the five methods while mitigating their respective limitations, such as validating the primary motif of a TF from the often noisy but genomic binding sites acquired by ChIP-seq with the high information content motifs from HT-SELEX. Additionally, the obtained data were analyzed with multiple motif discovery tools and conservatively hand-curated^17^. The resulting motifs were then benchmarked^21^ to identify the most robust binding models.

Here, we report the SMiLE-seq-based findings of the Codebook/GRECO-BIT collaboration, having assayed 284 putative TFs and derived binding models for 74 TFs. To address the hypothesis that some of the TFs for which no DNA binding motifs were recovered may in fact bind epigenetically modified DNA instead, we developed methylation-sensitive SMiLE-seq (“meSMiLE-seq”), an assay to simultaneously probe a TF’s affinity to unmethylated and methylated DNA. By screening 114 putative TFs using meSMiLE-seq, we identified DNA motifs that include information about (me)CG affinity for 48 TFs out of which 23 were also screened with “classical” SMiLE-seq, thus yielding 74 (SMiLE-seq) plus 25 (meSMiLE-seq) motifs. In addition, we derived motifs for several TFs that exclusively associate with methylated DNA, rationalizing why they remained orphan in the canonical DNA binding assays that were employed within the Codebook Consortium. Finally, we provide evidence that certain methylation-sensitive TFs are directed to distinct binding sites in the genome in a CG methylation-dependent manner, thus validating our in vitro observations.

## Results

### SMiLE-seq identifies binding motifs of 74 putative human TFs

We aimed to define the DNA binding specificities of 284 putative, uncharacterized TFs (selected through manual curation and including 3 positive controls, see **Supplementary Data 1** and^17^) using SMiLE-seq^22^. In this assay, DNA libraries are added to in vitro translated TF-GFP fusion proteins and transferred into a microfluidic device, where binding events between the TF and DNA are captured after a single enrichment step. Subjecting the TF-bound fraction to high-throughput sequencing of eluted DNA fragments then allows for computational de novo motif discovery (Supplementary Fig. 1a, b)^22^. Compared to alternative approaches such as HT-SELEX, SMiLE-seq has the advantage of capturing both strong and weak interactions without compromising the sampling space, such as in PBMs^23^. However, given the single round of enrichment, SMiLE-seq is sensitive to synthesis-related, skewed nucleotide distributions in the input libraries since these can obscure true TF binding signals (Supplementary Fig. 2a).

**Fig. 1:**
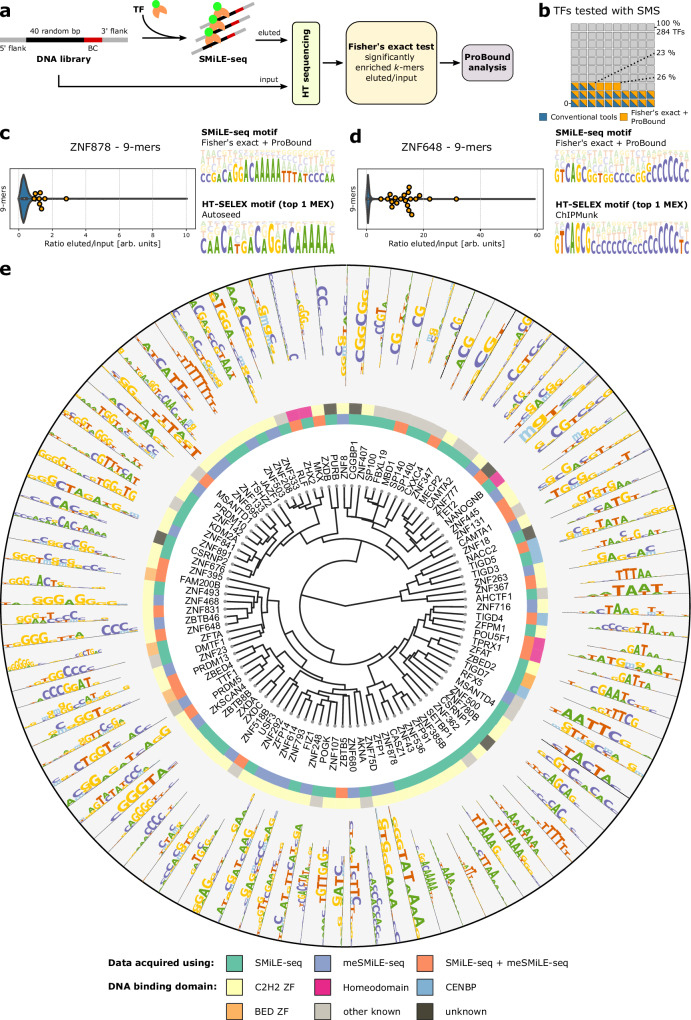
SMiLE-seq identifies DNA binding motifs for 99 putative human TFs. **a** Schematic description of SMiLE-seq: DNA libraries are incubated with a TF of interest and transferred to the microfluidic device where interactions between DNA and TF are captured and sequenced (eluted fraction). Naïve DNA libraries are sequenced without TF enrichment (input fraction). Significantly enriched *k*-mers in the eluted fraction are identified using a one-sided, right-tailed Fisher’s exact test, with the input serving as a background distribution. Raw sequences containing significant *k*-mers are then analyzed using the ProBound Suite to infer TFBS. **b** Waffle chart (10  × 10 grid, each square represents 1%) showing the proportion for which SMiLE-seq datasets (100% = 284 TFs) DNA binding motifs were successfully inferred. 65 binding models (23%) were retrieved with standard motif discovery pipelines as described in ref. ^21^, whereas our analytical strategy yielded high-quality binding models for 74 TFs (26%). **c**, **d** Violin plots depict the distributions of normalized 9-mers in the eluted fraction from one SMiLE-seq experiment (*n* = 253,133 unique 9-mers for ZNF878, *n* = 254,674 for ZNF648), calculated as a ratio of counts in the eluted versus input fraction (eluted/input, arbitrary units), with the most significant 9-mers shown as yellow dots (top 8 for ZNF878, top 20 for ZNF648). DNA motifs generated using the ProBound Suite after processing steps described in **a)** yield similar binding motifs as top ranked motifs reported in the collaborator’s study (abbreviated with MEX), generated by orthogonal experimental methods^21^. Statistical significance assessed for each 9-mer via one-sided Fisher’s exact test (eluted vs input) with Benjamini-Hochberg correction (FDR < 0.05); violin plots show full distribution; embedded boxes indicate the median (center line), interquartile range (box), and whiskers extend to values within 1.5 IQR. **e** Radial dendrogram based on motif similarity of all TF binding motifs generated by SMiLE-seq and meSMiLE-seq (see below), with TF family annotation. The clustering was performed using unmethylated motifs for consistency, however, where available, the displayed motif is the meSMiLE-seq-derived PSAM motif. For visualization purposes, only positive PSAM values are shown. Radial dendrogram was plotted with motifStack^89^. See also Supplementary Figs. 1 and 2.

To mitigate noise from these sequence biases introduced during library synthesis, we applied a one-sided Fisher’s exact test independently for each of the 284 TFs. This allowed the selection of sequencing reads enriched for statistically overrepresented *k*-mers in the eluted fraction relative to input (Fig. 1a, Methods). These filtered sequences were then passed to the ProBound Suite, a recently developed motif discovery pipeline that proved to be powerful in predicting binding sites across a wide range of affinities^21,24^ (Fig. 1a).

Using this approach, we successfully identified binding motifs for 74 TFs (26% of all assayed TFs, including one positive control), achieving a high level of replicate reproducibility (Supplementary Fig. 2b, e). By comparison, direct motif discovery on unfiltered data using conventional motif discovery tools such as Dimont, HOMER, or MEME^21,25–27^ yielded only 65 approved binding models (22.8%). This showcases the benefit of explicitly improving the signal-to noise ratio before motif discovery by filtering for significantly enriched *k-*mers (Fig. 1b). In fact, several SMiLE-seq datasets did not yield consistent motifs when analyzed with conventional tools and were thus excluded by the purposefully conservative curation and benchmarking of the Codebook Consortium. However, our analytical strategy linked 17 of these datasets to seemingly valid binding motifs, including novel models for 9 TFs that are uniquely reported in this manuscript (Supplementary Data 1 and 2). These motifs either exhibited high replicate concordance (e.g., ZNF385B, Supplementary Fig. 2b) or matched independently derived binding models from orthogonal experimental methods. For example, our approach extracted a similar motif for ZNF878 from SMiLE-seq data as was inferred from HT-SELEX data (Fig. 1c), whereas unfiltered processing of the same SMiLE-seq data by conventional tools failed to derive this exact motif (Supplementary Fig. 2f). Importantly, even for some approved datasets, combining pre-filtering of reads based on *k*-mer enrichment with the ProBound Suite allowed the identification of the TF’s full-length binding site while the same datasets only yielded truncated models when analyzed with other tools, such as for ZNF648 (Fig. 1d and Supplementary Fig. 2g and^21^). In total, our SMiLE-seq analyses and linked analytical strategy yielded binding motifs for 74 TFs. Using a modified version of SMiLE-seq (described below), we derived additional binding models, characterizing in total 99 TFs for DNA-binding. These 99 TFs span 14 different TF families, with C2H2 ZNFs being the most represented (Fig. 1e, Supplementary Fig. 2h and Supplementary Data 1).

### Development and implementation of methylation-sensitive SMiLE-seq

**Fig. 2:**
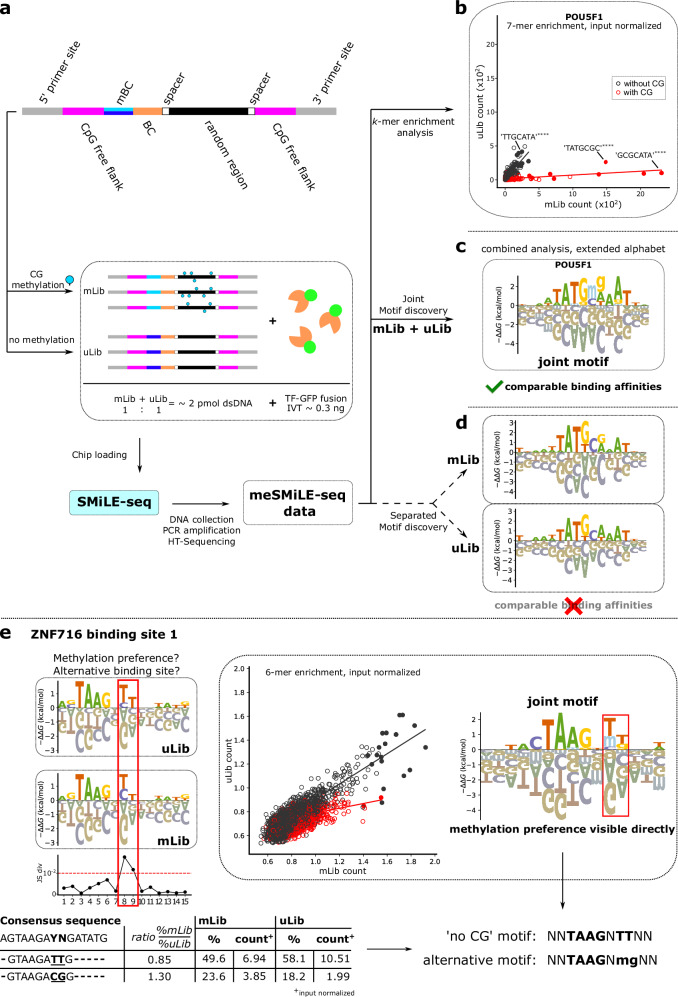
The workflow of meSMiLE-seq experiments. **a** Each meSMiLE-seq library carries two distinct molecular barcodes (mBC and BC) and a random region comprising 24 nucleotides. To mitigate any potential affinity towards CG dinucleotides not linked to the random region, the core library is flanked by CG-free regions. According to mBC, libraries (mLib) are split and enzymatically methylated before being combined with their unmodified counterpart (uLib) and exposed to in vitro translated TF of interest. After a single entrapment, captured DNA is collected, amplified, and sequenced. **b** The scatterplot shows the correlation of normalized *k*-mers (eluted/input) for POU5F1 from methylated (mLib, *x*-axis) and unmethylated (uLib, *y*-axis) libraries, with each circle representing a 7-mer, filled circles are the 20 most significant 7-mers. Black and red colors represent the absence or presence of a CG dinucleotide within the 7-mer, respectively. Significant enrichment is tested using a one-sided Fisher’s exact test. ****P < 0.00001. **c** Gibbs free energy motif generated by the ProBound Suite for POU5F1. Methylation sensitivity is depicted using an extended alphabet, where “mg” represents methylated CG dinucleotides. “g” also indicates methylation of the complementary cytosine. **d** Two independent free energy logos generated by ProBound for POU5F1. meSMiLE-seq data was split according to mBC. **e** ***Left:*** The two independent uLib and mLib DNA logos for ZNF716 do not clearly reveal methylation preference at divergent positions 8 and 9 (based on Jensen-Shannon divergence, JS div). Enrichment was assessed using exact matches to the 8-mer “GTAAGAYNG” (“Y” symbolizes a pyrimidine base, “N” any base). While “GTAAGATTG” was dominant in both libraries, “GTAAGACGG” was more enriched in mLib. ***Right:*** This preferential binding is directly apparent in the joint free energy motif with the extended alphabet. See also Supplementary Figs. 1, 3–5.

Despite extensive profiling attempts in our collaborative Codebook efforts, 155 putative TFs could still not be linked to binding motifs regardless of the profiling method (i.e., ChIP-seq, HT-SELEX, SMiLE-seq, etc.)^17^. We hypothesized that some of those might prefer binding to epigenetically modified DNA instead, as the methylation of CG dinucleotides can alter a TF’s specificity and affinity for a DNA sequence^12,15^. To concurrently infer DNA binding motifs and the methylation preference of TFs, we extended SMiLE-seq to allow for a methylation-aware motif discovery (a novel workflow that we will refer to as “meSMiLE-seq”). Specifically, we redesigned the bait DNA libraries to expose a TF to “naked” and modified DNA simultaneously (Fig. 2a). Each DNA library thereby contains two unique molecular barcodes that provide information about the position on the microfluidic chip (BC) and its modification status (mBC). Libraries that carry a methylation barcode are enzymatically methylated prior to the experiment (methylated library, mLib) before being combined in equimolar amounts with the corresponding, unmodified counterpart (unmethylated library, uLib) and added to in vitro translated TF-GFP fusion proteins (Fig. 2a, Supplementary Fig. 1, and Methods). Near-complete methylation of CG dinucleotides within mLib was confirmed using digestion with the methylation-sensitive restriction enzyme *BstBI* (Supplementary Fig. 3a, b). Considering the observed effect of input library biases in classical SMiLE-seq and the resulting challenges for data analysis, we compared different suppliers and synthesis protocols and chose input libraries with near-uniform *k*-mer distributions (Supplementary Fig. 3c, d, Methods). This rigorous control removed the need to prefilter sequencing reads, as was required for our SMiLE-seq data described above. Since most conventional motif discovery tools do not support DNA modifications, conveying position-specific information about DNA methylation using a position frequency matrix (PFM) inferred with classical tools is challenging. To overcome this issue, we used the ProBound Suite, which accommodates DNA modifications, and represents meSMiLE-seq-derived DNA binding motifs as Gibbs free energy logos (ddGs), inferred from position-specific affinity matrices (PSAMs)^28^ (Methods). For simplicity, we refer to these energy-based models as PSAM motifs throughout the manuscript. For each TF, up to three binding modes were modeled, and derived motifs are reported as “binding site 1”, “binding site 2”, etc., where applicable (Methods).

As a proof of concept for meSMiLE-seq, we profiled the TF POU5F1 (also known as OCT4), since it has previously been shown to bind to both methylated and unmethylated DNA motifs that direct its genomic location, making it a valuable positive control^12,29^. Using meSMiLE-seq, we found that POU5F1 enriches two distinct DNA *k*-mer species, correctly recapitulating its known genomic consensus binding sites “TATGCAA” and “ATG(meCG)CAT”^12,29^. The methylation-independent sequence “TATGCAA” (and its reverse complement “TTGCATA”) was equally frequent in uLib and mLib, while “G(meCG)CATA” was much more prevalent in mLib compared to any unmethylated sequence including “GCGCATA” in uLib, showcasing the protein’s strong preference only for the methylated version of the motif (Fig. 2b). Given meSMiLE-seq’s “one-pot reaction” approach, our data enables a more precise estimation of actual binding preferences toward methylated vs. unmethylated DNA, since uLib and mLib compete directly for TF binding. As a result, *k*-mer enrichment scatterplots directly indicate the DNA species preferentially bound by the TF. To also convey this information in PSAM logos, we used an extended alphabet in which “mg” represents a methylated “CG” dinucleotide (with “g” indicating the methylated “C” on the reverse strand)^28^ (Fig. 2c, Methods). Alternatively, meSMiLE-seq data can be stratified by molecular barcodes indicating library type (mBC) and analyzed separately, yielding two independent motifs for unmethylated and methylated binding events (Fig. 2d). In this separate analysis, we used the conventional alphabet (i.e. without methylation encoding) to better mirror standard motif discovery pipelines. However, interpreting methylation sensitivity based on these two independent motifs can be challenging, especially for TFs that exhibit only subtle methylation-dependent effects. For example, while POU5F1 shows a clearly distinct methylation-dependent binding site even without joint motif modeling (Fig. 2d), other TFs, such as ZNF716 (part of the extended TF library introduced below), exhibit more nuanced differences. Specifically, in the case of ZNF716 binding site 1 (Fig. 2e), the individual uLib and mLib motifs do not immediately suggest differential binding or a distinct methylation preference. Yet, a positional analysis using Jensen-Shannon divergence (Methods) revealed differences at positions 8 and 9. In both libraries, the most enriched sequence was “GTAAGATTG” (mLib: 6.94, uLib: 10.51, input normalized counts). However, the sequence “GTAAGACGG” showed preferential enrichment in the methylated library (mlib: 3.85, ulib: 1.99), suggesting that ZNF716 primarily recognizes a “TAAGNTT” motif, but also exhibits higher affinity for methylated “TAAGNCG” compared to the unmethylated version of the sequence. This subtle preference is more readily captured by the joint motif model (Fig. 2e), motivating its use in all subsequent analyses.

To further validate but also better place meSMiLE-seq in the context of existing methylation sensitivity motif profiling approaches, we examined three more positive controls with known methylation sensitivity next to POU5F1. We included RFX5 and ZNF23 as in vitro methylation-dependent binders, and ZNF263 as a methylation-independent control. Consistent with prior profiling^12^, meSMiLE-seq correctly recapitulated binding sites of these TFs, enriching the methylated sequences and predicting methylation-aware motifs for RFX5 and ZNF23, while identifying the canonical, methylation-independent motif for ZNF263 (Supplementary Fig. 4). We then benchmarked these results against a closely related methodology that combines HT-SELEX, methyl-SELEX, and bisulfite-SELEX^12^. This multi-step strategy assays TFs separately on unmethylated and methylated DNA across multiple enrichment cycles (typically two to four), followed by motif discovery (Supplementary Fig. 5a). However, this design can complicate the classification of methylation-sensitive TFs, as even weak CpG interactions can yield motifs, particularly in later enrichment cycles^12^. To resolve such ambiguity, an additional bisulfite-SELEX step is required after pre-enrichment to quantify CpG methylation effects (Supplementary Fig. 5b). While TFs like POU5F1 (as noted above), and to a lesser extent RFX5, show clear methylation-specific binding differences that can be detected without bisulfite-SELEX (Fig. 2d and Supplementary Fig. 4), ZNF23 requires this extra step for accurate classification^12^. meSMiLE-seq, by comparison, resolves these binding differences in a single experiment, after just one enrichment step and without bisulfite conversion (Supplementary Fig. 5c). These findings illustrate how meSMiLE-seq not only recapitulates known methylation-sensitive binding behaviors but also streamlines and strengthens motif discovery compared to existing multi-step strategies.

### Methylation-sensitive screening of poorly characterized human TFs

Next, we applied meSMiLE-seq to 114 TFs, profiling in total 128 “protein constructs”, i.e., 83 full-length proteins (FL) and 45 isolated DBDs (Supplementary Data 1 and Supplementary Fig. 3e, f). The screen included three subsets: (i) 70 randomly selected Codebook/GRECO-BIT constructs (33 FL, 37 DBDs, “set 1”) from both approved and non-approved datasets; (ii) 23 constructs (15 FL, 8 DBDs, “set 2”) for TFs that previously yielded motifs only in ChIP-based assays, which may imply interaction with epigenetically modified DNA; and (iii) 35 lab-available KRAB-ZNFs (“set 3”), a class of TFs often associated with heterochromatin-associated regions in ChIP-exo^30^ and, therefore, additional candidates for methylation sensitivity. Among the TFs tested by meSMiLE-seq, 23 overlapped with those profiled by classical SMiLE-seq. Overall, meSMiLE-seq yielded high-confidence binding models for 48 TFs: 27 TFs from “set 1”, 4 from “set 2”, and 17 from “set 3” (Fig. 1e, Supplementary Fig. 3e, f).

**Fig. 3:**
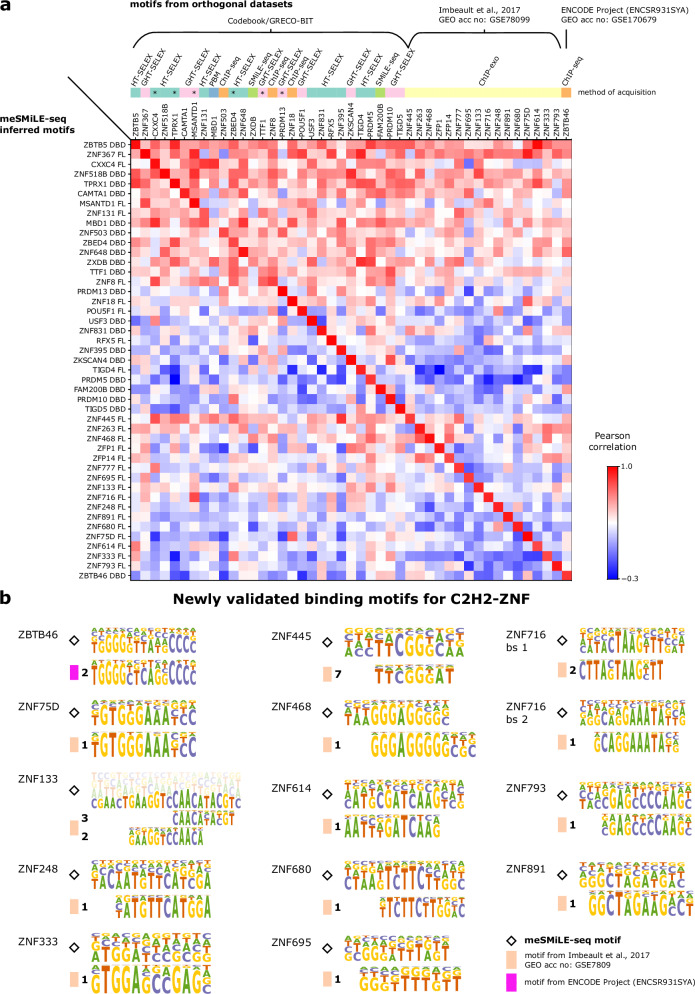
meSMiLE-seq-derived motifs correlate highly with orthogonal datasets. **a** Correlation matrix between meSMiLE-seq-derived PFMs and PFMs generated by orthogonal datasets, i.e., the Codebook/GRECO-BIT consortium^17^, and the ENCODE Project^90^, expressed as Pearson correlation coefficients. For HT-SELEX and GHT-SELEX, TFs were either expressed as GFP fusion proteins using an IVT-kit or expressed in HEK293 cells (marked with an asterisk “*”)^19^. **b** meSMiLE-seq-inferred binding motifs (generated using ProBound, displayed as PFMs) validate binding models for C2H2-ZNFs that were previously only assayed by ChIP-seq or ChIP-exo. The digit indicates the rank of the found motif generated by HOMER.

To confirm the overall validity of the meSMiLE-seq-derived motifs and the robustness of the assay, we used several approaches. First, we asked whether the meSMiLE-seq PSAMs are consistent with orthogonal datasets where methylation status was not explicitly considered, and for this comparison, we excluded the extended, methylation-specific alphabet (Methods and Supplementary Fig. 6a). meSMiLE-seq motifs showed high concordance with binding models predicted by orthogonal in vitro and in vivo methods, achieving an average Pearson correlation coefficient (PCC) of 0.899 across all TFs (based on values in aligned probability matrices) (Fig. 3a, Supplementary Data 3 and^17^). We observed that TFs with longer motifs, such as PRDM10 or ZNF793, do not display strong similarity towards other TFs, even within their respective TF families (Figs. 1e and 3a). This was especially noticeable for C2H2 ZNF proteins, consistent with the expected uniqueness of their binding sites^30,31^. Conversely, some TFs displayed seemingly highly similar motifs, such as CXXC4 and MBD1, both recognizing short, CG-containing sequences. However, their binding specificities differ with respect to DNA-methylation: MBD1 binds methylated DNA, whereas CXXC4 binds unmethylated DNA, which standard PFMs without extended alphabets fail to capture (Supplementary Data 4). Second, we compared the meSMiLE-seq motifs of 13 C2H2 ZNFs to those derived from ChIP-seq^18,32^ and ChIP-exo data^30^ (Supplementary Data 3) using HOMER de novo motif enrichment analysis for these same TFs (Fig. 3b). Importantly, while most of the first-ranked (i.e. most significant reported by HOMER) ChIP-seq-inferred motifs resembled meSMiLE-seq-derived models (as indicated by the digit next to the PFMs), this was not the case for ZBTB46 (second-ranked), ZNF133 (second- and third-ranked), and ZNF445 (seventh ranked) (Fig. 3b), illustrating the value of orthogonally validating ChIP-derived binding sites using in vitro assays. These findings align well with the observation that an estimated ~25% of all, most significant motifs reported by traditional motif discovery tools such as HOMER do not represent the “true” binding sites of the studied TFs^21,33^. Instead, these motifs likely reflect those of co-factors (e.g., in ChIP-seq) or over-amplified DNA sequences due to method-related errors or biases (e.g., in HT-SELEX)^21^.

### Classification of TFs based on methylation sensitivity inferred from meSMiLE-seq

**Fig. 4:**
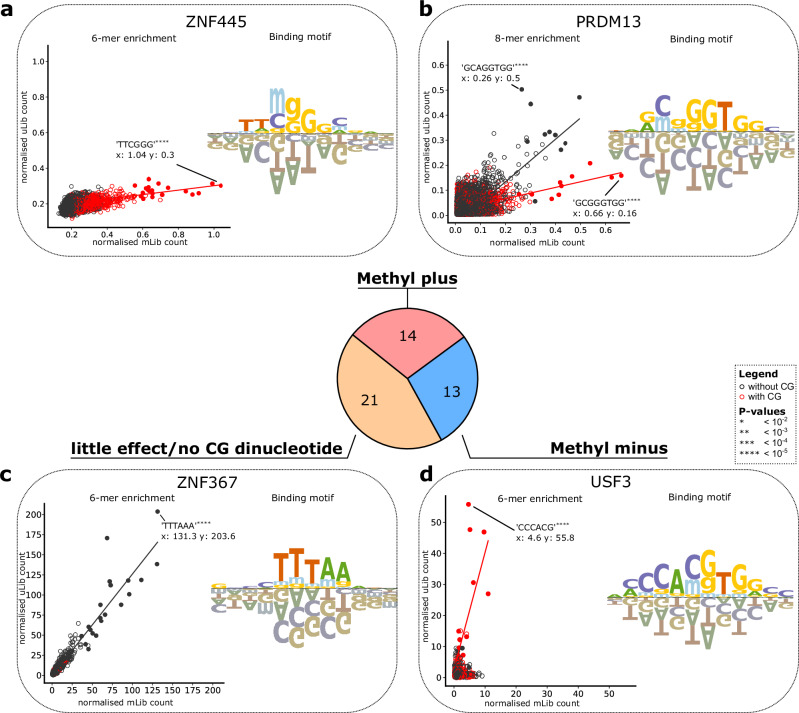
TF classification based on affinity towards methylated DNA. Categorization of TFs into three groups based on methylation sensitivity. “Methyl plus” TFs show **a)** higher affinity upon DNA methylation (e.g., ZNF445) or **b** alternative binding sites containing methylated CG (e.g., PRDM13). **c** Without an observable methylation-specific effect, or if TFs do not bind a CG-containing sequence, they are categorized as “little effect/no CG dinucleotide” (e.g., ZNF367). **d** “Methyl minus” TFs show decreased affinity toward methylated DNA (e.g., USF3). Depicted are correlation scatterplots and PSAMs as described in Fig. 2b, c. Statistical significance was assed via one-sided Fisher’s exact test (eluted vs. input) with Benjamini-Hochberg correction; ****P < 0.00001.

We next classified the 48 TFs with characterized binding motifs into three distinct groups depending on their affinity for methylated sequences based on ProBound predictions (see Methods), which we will refer to as “methyl plus”, “methyl minus”, and “little effect/no CG”^12^. Classifications were derived from joint motifs and scatterplots, and further validated using the workflow shown in Fig. 2e. In brief, for each divergent and C- or G-containing position in uLib and mLib motifs, all possible single-nucleotide permutations of the consensus sequence were assessed for relative enrichment in uLib and mLib (Supplementary Data 5, Methods). “Methyl plus” TFs (*n* = 14) demonstrated a greater attraction towards methylated CG dinucleotides compared to unmethylated DNA, such as ZNF445, a genomic imprinter predicted to bind methylated DNA in vivo^34^ (Fig. 4a and Supplementary Fig. 6b). Additionally, several TFs in this group enriched multiple consensus sequences. PRDM13 for example recognized the methylation-independent “GCAGGTGG” as well as the methylated “G(meCG)GGTGG”, similar to the dual binding behavior observed for POU5F1 (Fig. 4b and Supplementary Fig. 6c). TFs that interacted with sequences regardless of their methylation status or preferred motifs without CGs were classified as “little effect/no CG”, such as ZNF367 (Fig. 4c). In contrast, “methyl minus” TFs (*n* = 13) exhibited a reduced affinity to methylated DNA (e.g., USF3) (Fig. 4d and Supplementary Fig. 6d, see also Supplementary Data 4 and 5).

To provide further support for our meSMiLE-seq-derived findings, we performed electrophoretic mobility shift assays (EMSA) for PRDM13 (methyl plus) and USF3 (methyl minus). PRDM13 caused DNA shifts with both its “methyl plus” and “no CG” motifs, while USF3 exclusively engaged in binding with its motif when unmethylated, supporting the results from meSMiLE-seq (Supplementary Fig. 7).

### The methylation of binding sites dictates the genomic distribution of TFs

**Fig. 5:**
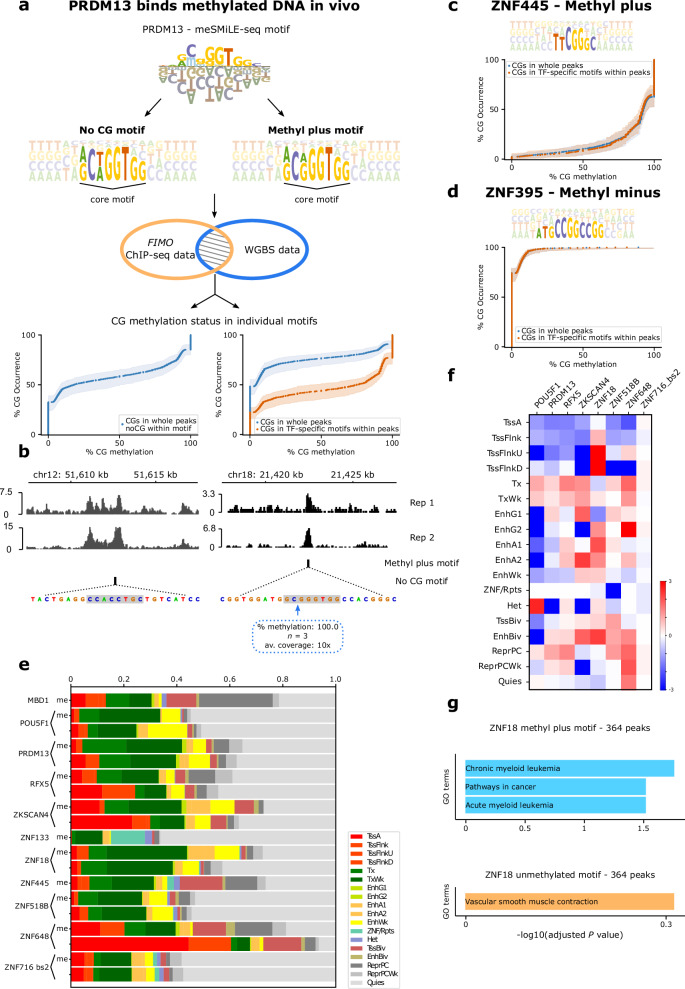
Methylation of TFBS drives genomic distribution of TFs. **a** Exemplary workflow to identify methylated TFBSs in cells, illustrated with PRDM13. “Methyl plus” and “no CG” motifs are extracted as PFMs from meSMiLE-seq-derived PSAMs and mapped to PRMD13-specific ChIP-seq peaks. Individual motif instances are intersected with WGBS data, and CG methylation levels plotted as ECDFs. Orange points mark CGs located within the motif; blue points mark CGs elsewhere in the same ChIP-seq peak. For motifs lacking CGs (“no CG motif”, left), only blue points are shown. **b** IGV browser snapshots for a “no CG” and “methyl plus” motif found in PRDM13-specific ChIP-seq peaks. **c**, **d** ECDFs depicting methylation levels of motifs for ZNF445 and ZNF395. For **a**, **c**, **d** the central line indicates the median; shaded bands represent the full bootstrap range (minimum–maximum across 1,000 bootstrap iterations), obtained by resampling up to 1000 data points with replacement. **e** ChromHMM annotations of TF-peak-specific motifs of “methyl plus” TFs identified as described in (**a**). “me” describes “methyl plus” motifs, while the lack of “me” represents “no CG” or unmethylated motifs. Tss: transcription start site, TssBiv: bivalent/poised transcription start site, Tx and TxWk: actively transcribed genes, EnhBiv: bivalent enhancer. The full legend for abbreviations can be found in Supplementary Fig. 8b**. f** Heatmap of TF-specific ChromHMM annotations expressed as log2-transformed ratios (“methyl plus”/”no CG”). **g** Gene ontology enrichment analysis of ZNF18’s “methyl plus” (at least 50% methylated) motifs (364 peaks in total) and “no CG” motifs (364 most significant peaks). Adjusted P values were calculated with Fisher’s exact test and corrected via the Benjamini-Hochberg method. See also Supplementary Fig. 8.

Next, we investigated whether our in vitro “methylation (in)sensitivity” assessments could be validated in a cellular context. We searched for individual occurrences of meSMiLE-seq-derived motifs for each TF within corresponding ChIP-seq^18^ or ChIP-exo^30^ data in HEK293 and HEK293T cells (for simplicity, abbreviated as HEK293/T) (Supplementary Data 3). To obtain information on the degree of methylation at the genomic loci that contained relevant motifs, we intersected the regions with publicly available WGBS data for both cell lines^35,36^ (Supplementary Data 3). We first focused on PRDM13, as PRDM13 binds both unmethylated and methylated DNA in different sequence contexts in vitro, and both of these binding sites were abundantly found in ChIP-seq peaks (Supplementary Fig. 8a). The absence of CG dinucleotides in the “no CG” motif “GCAGGTGG” prompted us to examine CG methylation across entire peaks containing this motif. Roughly 45% of CGs in these peaks were methylated at less than 10%, while ~25% exceeded 90% methylation (Fig. 5a, b). By contrast, over 38% of occurrences of PRDM13’s “methyl plus” motif “GCGGGTGG” within ChIP-seq peaks were found to be highly methylated (>90%) in HEK293 cells. This enrichment is particularly striking because methylation was generally low across the broader set of peaks harboring the “methyl plus” motif (i.e., ~64% showing less than 10% methylation and only ~14% exceeding the 90% methylation threshold; Fig. 5a, b, Methods). Together, these findings indicate that PRDM13 binds both meSMiLE-seq-predicted motifs in a methylation-dependent context in vivo. We expanded the analysis to include all TFs with available ChIP-seq^18^ or ChIP-exo data^30^. The number of significant motif hits within peaks varied, and was influenced by core-motif length (e.g. 12 bp for ZNF75D and 4 bp for CXXC4), information content, and data quality, amongst others, resulting in an uneven distribution of significant motif hits (Supplementary Fig. 8a). We observed similar patterns to those of PRDM13 for “methyl plus” TFs ZNF445 (Fig. 5c), POU5F1, ZNF716, ZNF18 and ZNF518B (Supplementary Data 6). Interestingly, while RFX5 successfully served as a positive control for a “methyl plus” TF in meSMiLE-seq, showcasing affinity to both methylated and unmethylated motifs in vitro (Supplementary Fig. 4), its genomic TFBSs were not methylated in HEK293 cells, consistent with previous observations in different cell lines^37^. Similar discrepancies were noticed for the “methyl plus” predicted TFs ZKSCAN4, ZNF133, ZNF503, and ZNF648, which bound predominantly unmethylated regions in HEK293/T cells (Supplementary Data 6). These data suggest that while a TF’s ability to bind methylated DNA in vitro is a prerequisite for binding the modification in vivo, it does not guarantee that this behavior will be observable in all cell types.

In contrast, motif occurrences for most “methyl minus” TFs (11 of 13) were predominantly unmethylated in cells, as illustrated by ZNF395 (Fig. 5d and Supplementary Data 6), suggesting that these proteins might, in general, have a weakened affinity towards their motifs when methylated, irrespective of cellular context.

Given that several TFs were found to associate with unmethylated and methylated DNA motifs with distinct sequences, we aimed to assess the genomic impact of the different binding profiles of these TFs. We intersected the binding sites of all TFs with available ChIP-seq or ChIP-exo data with ChromHMM annotations for HEK293/T cells^38^ (Supplementary Fig. 8a–c), but focused especially on “methyl plus” TFs to compare the genomic annotations of highly methylated motif occurrences (motif occurrences of “methyl plus” motifs that are at least 50% methylated in WGBS) to their unmethylated counterparts (i.e., “noCG” motifs) (Fig. 5e, f). The analyses revealed that methylated binding sites are mostly depleted around active transcription start sites (Tss) (except ZNF716 and ZNF18) while being enriched in most cases in bivalent/poised regulatory elements (TssBiv and EnhBiv) and actively transcribed genes (Tx and TxWk). These observations support the methylation status of these DNA elements (Fig. 5f) as they tend to be consistent with previous findings, such as heavy methylation of gene bodies of actively transcribed genes^39–42^. Annotation of the genomic regions using gene ontology analysis of nearby genes (<3 kb distance of the TFBS) also showed that different DNA motifs may direct TFs to distinct classes of genes and may thus contribute to the differential regulation of cellular function. This is illustrated by ZNF18, a poorly characterized KRAB-ZNF whose ZNF18 “methyl plus” motifs are significantly associated with “pathways in cancer” and more specifically with “chronic/acute myeloid leukemia” (Fig. 5g), which in turn might rationalize its involvement in the pathogenesis of various malignancies^43–45^. Although gene ontology enrichment for many other TFs was inconclusive, as most pathways did not surpass the significance threshold (Supplementary Fig. 8d–g), our analyses nevertheless suggest that methylation-sensitive TFs are recruited to different genomic locations based on their affinity towards DNA methylation and thus exert their regulatory function in different cellular contexts.

### ZHX2 as a putative Z-DNA binding protein

**Fig. 6:**
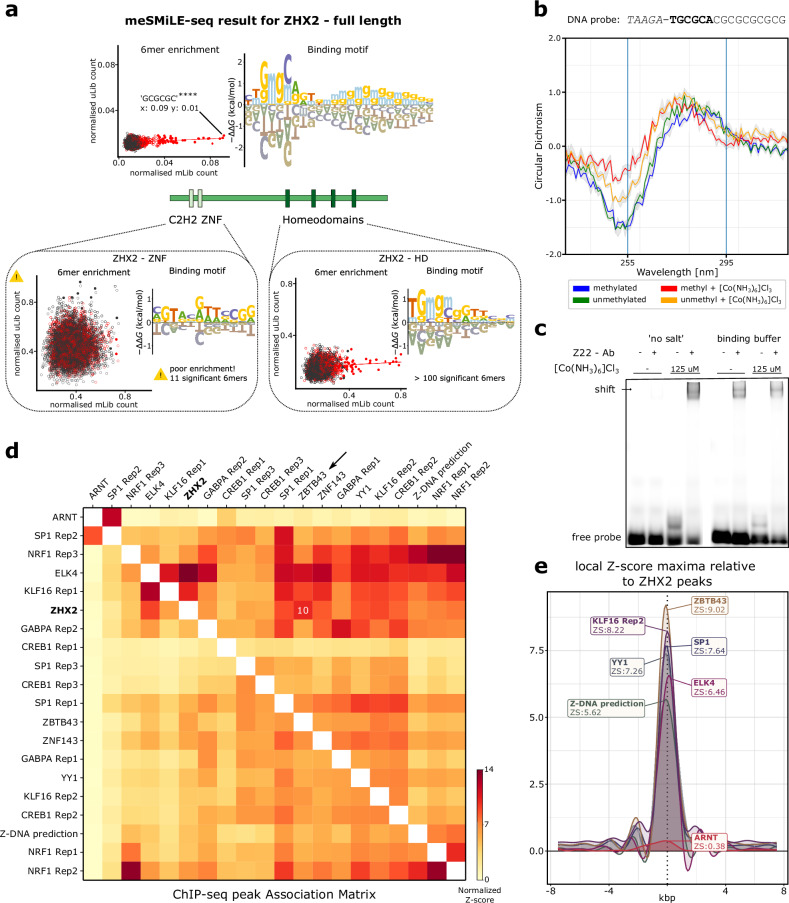
ZHX2 as a putative Z-DNA binding protein. **a** Schematic structure of ZHX2, depicting its C2H2 ZNFs and homeodomains (HDs). The full-length protein and isolated HDs, enrich methylated CG repeats in meSMiLE-seq, showcased by correlation scatterplots and PSAMs as described in Fig. 2. In contrast, ZNFs display poor enrichment, suggesting minimal, if any, DNA-binding capacity. **b** CD spectroscopy (ellipticity) of meSMiLE-seq-derived ZHX2 binding sites plotted in function of the wavelength. The data show B-Z transition as seen by the upshift and downshift in ellipticity at 255 nm and 295 nm, respectively. Blue and red lines: methylated DNA; green and orange lines: unmethylated DNA. Red and orange lines: added hexaaminecobalt(III) chloride. The central line represents the mean ellipticity; shaded areas indicate the full range (minimum-maximum) across three technical replicate measurements of the same sample. **c** Hemi-methylated meSMiLE-seq-derived ZHX2 binding site (same as in **b**)) forms Z-DNA in 1x EMSA binding buffer even without addition of hexaaminecobalt(III) chloride, as seen by the band-shift after addition of anti-Z-DNA (Z22) antibody. “no salt” buffer: 10 mM Tris-HCl, 1 mM DTT, 0.5 % NP40, 0.05 mg/mL BSA and 10 pmol uLib; binding buffer: 10 mM Tris-HCl, 10 mM NaCl, 40 mM KCl, 1 mM MgCl2, 1 mM EDTA, 1 mM DTT and 0.05 mg/mL BSA and 10 pmol uLib. EMSA shown is from a single independent experiment. **d** Heatmap shows ChIP-seq peak associations between TFs in HepG2 cells expressed as normalized Z-scores. ZHX2-specific peaks display highest scores with ZBTB43 peaks. **e** Local Z-scores between selected TFs and ZHX2 within a 7.5 kbp neighborhood. The sharp drop indicates a central, peak-specific association instead of lateral, region-wide overlaps.

Among the detected “methyl plus” TFs, we found one TF (zinc fingers and homeoboxes protein 2 (ZHX2)), which encodes two C2H2 ZNFs and four or five homeodomains (HDs)^46,47^ that specifically enriched methylated “CG” repeats when expressed as a full-length protein in meSMiLE-seq. To locate the DNA interaction domains of this protein, we performed experiments using the DBDs separately and identified the HDs as mediating the observed DNA binding properties (Fig. 6a). However, when comparing our data to that of ChIP-seq-derived ZHX2 motifs, we found that the motifs were dissimilar, as the latter mainly comprised of typical promoter motifs similar to those of other promoter binders such as “TGACG” or “AAGATGG” for CREB1 and YY1, respectively^32,48^. Other reported, predicted genomic binding sites for ZHX2 included sequences such as “AGGCTAGA”^49^ or “CCACCAC”^50^. The methylated poly(CG) core of the ZHX2 motif derived from meSMiLE-seq is, however, reminiscent of DNA sequences involved in the formation of non-canonical DNA structures, particularly Z-DNA^51^. Z-DNA is a higher-energy, left-handed DNA conformation that can form under various conditions depending on sequence and environment^52^. Purine-pyrimidine repeats are particularly prone to adopting this conformation in vitro when stabilized by specific reagents or multivalent salts such as MgCl_2_ or hexaaminecobalt(III) chloride^53,54^, and methylation of CG repeats further stabilizes the Z-form as compared to unmethylated poly(CG)^51^. In mammalian genomes, Z-DNA-forming regions are enriched in promoters, where the structure is temporally formed due to negative supercoiling during transcription^55–57^.

To test whether the meSMiLE-seq-derived sequences may form Z-DNA, thus indicating that ZHX2 might preferentially bind to this particular DNA conformation, we performed circular dichroism (CD) spectroscopy. We found that both methylated and unmethylated sequences can transition from canonical B-DNA to Z-DNA when incubated with hexaaminecobalt(III) chloride. However, methylated DNA displayed a higher potential to transition as seen by a stronger upshift in ellipticity at 255 nm (Fig. 6b). Importantly, this transition also occurs without hexaaminecobalt(III) chloride in EMSA binding buffer that does not contain extreme amounts of salts (10 mM Tris-HCl, 10 mM NaCl, 40 mM KCl, 1 mM MgCl_2_, Methods) as captured by the anti Z-DNA Ab Z22 (Fig. 6c). This demonstrates that methylated “CG” repeats shift the equilibrium from B-DNA towards Z-DNA compared to unmethylated sequences. The observation also suggests that Z-DNA could form in a complex medium such as the Wheat Germ IVT-kit that is used for TF production in meSMiLE-seq experiments (Methods).

We then searched for evidence of Z-DNA formation in the genomic target sites of ZHX2 to further test if this DNA conformation was preferentially bound by the protein. We compared ZHX2-specific ChIP-seq regions with peaks from other TFs with similar motifs or with Z-DNA ChIP-seq profiles (Methods, Supplementary Data 3)^56^. Additionally, we included datasets of in silico predicted Z-DNA forming sites^57^ and of ZBTB43, a TF that has been recently identified as a Z-DNA remodeler in prospermatogonia^58^. Peak associations were calculated as the enrichment of observed overlaps between peak sets relative to random overlaps, estimated by genome-wide shuffling (1000 permutations, Methods). Most notably, ZHX2 exhibited the highest association scores and strongest local overlap with ZBTB43 across all tested TFs. These observations strongly suggest that ZHX2 is recruited to its genomic locations by recognizing Z-DNA conformations rather than canonical B-DNA motifs (Fig. 6d, e). Altogether, our meSMiLE-seq analysis revealed reproducible and distinct ZHX2 binding motifs that diverge from the generic, promoter-like motifs obtained from ChIP-seq, suggesting an alternative mechanism for ZHX2 recruitment to promoters in vivo. Because Z-DNA is a transient feature of mammalian promoters and predicted Z-DNA regions are not cell type-specific, ChIP-seq data alone lacked the resolution to implicate Z-DNA binding. It was precisely the contrast between the in vitro meSMiLE-seq motifs and the ChIP-seq derived motifs, together with the overlap of ChIP-seq peaks with a known Z-DNA binding protein, that enabled us to propose this hypothesis, highlighting the unique value of in vitro DNA binding assays such as meSMiLE-seq.

## Discussion

In this study, we investigated the DNA binding properties of 284 putative human TFs using SMiLE-seq and 114 TFs via meSMiLE-seq, identifying motifs for 99 TFs, thereby substantially expanding the TF “codebook”. One of the defining features of SMiLE-seq, most clearly demonstrated by meSMiLE-seq, is its reliance on a single round of DNA enrichment. Because multiple DNA species are simultaneously exposed to a TF and bound molecules are entrapped in a single MITOMI step, the assay captures actual binding differences, including subtle differences between modified and unmodified DNA. Importantly, the absence of multiple amplification cycles preserves the native methylation state of the DNA and ensures the detection of TFBSs across a wide affinity range. We further showed that meSMiLE-seq-derived motifs correlate strongly with binding models from orthogonal datasets when methylation information is excluded. However, methylation sensitivity is not captured by standard zero-order background DNA logos, which have been the gold standard for reporting DNA-binding sites for at least 35 years^59^, underscoring the need for extended alphabets or models that incorporate dinucleotide contexts. Since most TF-DNA interaction assays do not include modified DNA by default, there is clear value in re-examining even well-characterized TFs for methylation sensitivity. Here, meSMiLE-seq offers a key advantage: as a scalable competition assay operating under equilibrium conditions, it internally controls for assay performance through the unmethylated library, provided a focal TF’s canonical binding site is known. In this regard, an important goal will be to extend meSMiLE-seq to other DNA modifications that may influence TF-DNA interactions but remain less studied than CpG methylation, such as 5′-hydroxymethylation of cytosine (5hmC)^60^ or N6′-methylation of adenosine (N6mA)^61^.

The Codebook/GRECO-BIT initiative successfully identified DNA binding sites for 177 of 332 predicted human TFs (without controls). However, for 155 TFs, motif discovery did not yield reproducible motifs, suggesting a possible misclassification of proteins as TFs^1^, or a need for specific interaction partners, such as heterodimers, which were not considered within the Codebook/GRECO-BIT collaboration or in this study. Our analysis yielded 74 high-quality binding motifs from 284 TFs using SMiLE-seq, with an initial success rate of ~26% with our analysis approach or ~23% using non-customized motif discovery pipelines. The lower-than-expected yield was likely due to technical challenges, including a single round of DNA enrichment and bias in nucleotide distribution, which complicated motif discovery. By changing the DNA libraries and ensuring uniform nucleotide distributions in meSMiLE-seq, the success rate nearly doubled (~42%). The improvement was achieved despite the additional experimental complexity by exposing the TFs to both methylated and unmethylated DNA, although part of this increase may be influenced by using a subset of TFs in meSMiLE-seq that were suspected to bind DNA. Nonetheless, this highlights the importance of customized analysis pipelines for overcoming background noise and extracting accurate TFBSs, indicating that further optimization could significantly enhance future discoveries.

Why some TFs exhibit higher (or lower) affinity for methylated DNA remains poorly understood^62^. Only a limited number of TF-DNA crystal or cryo-EM structures have been solved due to experimental complexities (15.000 entries, i.e., 6% in the Protein Data Bank (PDB)), and virtually none capture interactions with methylated DNA (661 entries tagged with “DNA methylation” or “methylated DNA, i.e., 0.3% in PDB). Consequently, mechanistic insight relies heavily on computational modeling, yet current approaches also remain insufficient as they are trained on databases. Even state-of-the-art methods such as AlphaFold3 or RoseTTAFoldNA struggle to reliably model large, flexible, or novel TF-DNA complexes^63,64^, and accounting for methylation-dependent effects adds another layer of complexity. Nonetheless, many meSMiLE-seq-derived motifs were found in TF-associated ChIP-seq peaks in HEK293/T cells. Pairing the data with WGBS confirmed that predictions for most “methyl minus” TFs (11 out of 13) were correct, as motif occurrences for these TFs were predominantly not methylated in cells. Similarly, for “little effect” TFs, 8 out of 12 showed no significant difference in methylation distribution within their motifs compared to the surrounding ChIP-seq peaks. This analysis also showed that several “methyl plus” TFs (6 out of 11) bound methylated genomic regions. This indicates that not only can binding models acquired by in vitro methods, such as meSMiLE-seq, translate to the cellular context, but also that such TFs are likely involved in different regulatory pathways depending on the methylation status of their motifs. For example, gene ontology enrichment analysis for ZNF18 showed that its “methyl plus” binding sites were significantly associated with “Pathways in cancer”, consistent with the role of aberrant DNA methylation in transcriptional dysregulation and malignancy^65,66^. The observation that most “little effect” and some “methyl plus” TFs nevertheless bound largely unmethylated DNA in HEK293/T cells may be due to the inaccessibility of those motifs in a cell type-specific chromatin landscape. Since CG methylation is frequently associated with heterochromatin formation and gene silencing, TFs that lack “pioneering” capability, i.e., the ability to access nucleosome-occluded sites and/or remodel chromatin remodel, may be unable to engage their methylated motifs in vivo^65,67–69^. Another constraining factor might be competition for methylated binding sites between these TFs and other TFs with high methylated DNA affinities, such as MBDs, as suggested by previous studies^37^. In this case, binding to methylated motifs may be restricted to specific loci and cell types.

Lastly, our study identified ZHX2 as a potential Z-DNA binding protein, as it consistently enriched methylated purine-pyrimidine repeats. The stabilization of poly(CG) sequences in the Z-form by multivalent salts and cytosine methylation suggests that Z-DNA may be randomly adopted in meSMiLE-seq experiments where TFs are incubated with DNA for an extended time. While non-canonical DNA conformations like Z-DNA are already well known to impact gene regulation^70–72^, recent computational advancements have renewed interest in predicting genomic regions that are likely to adopt non-B-DNA structures and linking these structures to various cellular processes^57^. However, validating protein binding to these structures remains challenging due to their temporal instability under physiological conditions, and we acknowledge that our current evidence for ZHX2 remains correlational. In this regard, definitive structural data, though far from trivial to obtain, will eventually be required to fully establish ZHX2’s capacity to recognize Z-DNA. Nonetheless, meSMiLE-seq could offer a valuable platform to more systematically study these interactions. For example, incubating proteins with DNA to reach equilibrium and adding reagents to trigger specific DNA transitions would allow the comparison of eluted DNA with and without reagents to identify interactions with specific DNA structures.

Altogether, our work expands the human TF “codebook” and establishes meSMiLE-seq as a robust platform for profiling methylation-sensitive DNA binding. Beyond recapitulating known effects, it uncovers new regulatory modes, providing a foundation for linking DNA modifications and alternative structures to transcriptional control.

## Methods

### Experimental procedures

#### TF selection and plasmids

Transcription factors were provided as EGFP fusion proteins in pF3A WG (Promega) by the Codebook/GRECO-BIT collaboration^17^. Plasmids encoding additional KRAB-Zinc finger proteins were kindly provided by Didier Trono’s laboratory and were cloned into pDONR221 plasmids (ThermoFisher Scientific) as EGFP fusions and further shuffled into custom-made Gateway-compatible pF3A WG.

#### SMiLE-seq procedure

##### Library generation

SMiLE-seq libraries, comprising a random region of 40 nucleotides, and meSMiLE-seq libraries, comprising a random region of 24 nucleotides, were ordered as hand-mixed DNA oligos from IDT and resuspended to a concentration of 100 µM (Supplementary Data 7). dsDNA libraries were synthesized via enzymatic reaction. 3 µl library was mixed with 6 µl annealing_primer (100 µM), 3 µl 10x NEBuffer2 (NEB), and 18 µl ddH2O, and incubated for 5 min at 95 °C followed by 1 min at 60 °C. 20 µl were transferred into a new tube containing 16 µl ddH2O, 5 µl 10x NEBuffer2, 4 µl 10 mM dNTPs (Thermo), and 5 µl (25 units) DNA Polymerase I (Large Klenow Fragment) (NEB). The reaction was incubated for 60 min at 37 °C and subsequently purified and eluted in 13 µl ddH2O using a MinElute kit (Qiagen) following the manufacturer’s instructions.

##### Methylation of libraries for meSMiLE-seq

DNA libraries were treated differently depending on their respective mBCs (Supplementary Data 7). uLibs remained unmodified while mLibs were methylated by mixing 250 ng of DNA with 5 µl 1.6 mM SAM (NEB), 5 µl 10x NEBuffer2, and 1 µl (4 units) M.SssI (NEB) in a total volume of 50 µl (topped off with ddH2O), and incubated for 4 h at 37 °C followed by 20 min at 65 °C. To ensure a maximal degree of CG methylation, this step was performed twice. Libraries were purified using a MinElute kit (Qiagen).

The efficacy of the methylation protocol was verified by methylation of a control library (CL) (Supplementary Data 7) followed by enzymatic digestion using BstBI (NEB). 1 μg of both modified and unmodified CLs were mixed with 1 μl (25 units) enzyme, 5 μl 10x rCutSmart buffer (NEB) in a total volume of 50 μl and incubated for 15 min at 65 °C. The samples were analyzed via agarose gel electrophoresis using a 1% agarose gel (Thermo) in 1x Tris-Acetate-EDTA buffer stained with 1:10000 SYBR Safe (Thermo).

Subsequently, mLibs and uLibs were mixed in a 1:1 molar ratio and diluted 1:10 in ddH2O. Each library aliquot of 10 µl was mixed with 50 ng of poly-dIdC (Sigma). Aliquots were stored at −20 °C for a maximum duration of 3 months.

##### SMiLE-seq assay

The SMiLE-seq pipeline, including chip fabrication and functionalization, was carried out essentially as previously described in Isakova et al.^22^. In brief, TFs of interest were expressed as GFP fusion proteins using the TNT SP6 High-Yield Wheat Germ Protein Expression System from Promega (referred to as IVT-kit) following the manufacturer’s instructions. TFs were incubated with one aliquot of DNA library for at least 2 h at 25 °C and then transferred into the functionalized microfluidic device (detailed protocol Isakova et al.^73^), where mechanical trapping of molecular interactions was performed.

##### Post-experiment library purification

Recovered DNA was purified with a MinElute kit (Qiagen) and eluted in 20 μl elution buffer. The eluate was mixed with 32.5 μl NEBNext High-Fidelity 2x PCR Master Mix (NEB), 0.5 μl library_primer_fwd (10 μM), 0.5 μl library_primer_rev (10 μM), 0.5 μl 100x SYBR Green I (Thermo) in a total reaction volume of 65 μl. 50 μl of the reaction were kept on ice while 15 μl were used to estimate the suitable amount of amplification cycles using StepOnePlus Real-Time PCR instrument (Applied Biosystems) following the subsequent program: hot start at 98 °C for 30 s and 25 cycles of 98 °C for 10 s, 63 °C for 30 s and 72 °C for 1 min, followed by a final elongation at 72 °C for 1 min, then kept at 4 °C. The remaining 50 μl were amplified accordingly and purified with a MinElute kit (Qiagen). DNA was size-selected using 1.5x (one-sided) AMPureXP beads (Beckman Coulter) to remove primer dimers.

After purification, libraries were amplified for 5 cycles with 0.5 μl i5 and i7 Nextera adapters (10 μM) following the instructions provided by Illumina. Finally, DNA was purified (MinElute, Qiagen) and size selected using 1x (one-sided) AMPure XP beads (Beckman Coulter) to remove impurities.

Libraries were sequenced on Illumina MiSeq and NextSeq500 Python platforms. All data were acquired in four sequencing batches.

#### Electrophoretic mobility shift assays

Electrophoretic mobility shift assays of PRDM13 and USF3 were performed using precast 6 % TBE gels (Novex) and were run in 0.5% TBE buffer at 4 °C and 100 V. The TFs were expressed as GFP fusion proteins using the IVT-kit. 2 μl of unpurified IVT-TF solution was mixed with 0.1 pmol of Cy5-labeled DNA probe (IDT, Supplementary Data 7) in 1x binding buffer (80 ng poly-dIdC, 10 mM Tris-HCl, 10 mM NaCl, 40 mM KCl, 1 mM MgCl_2_, 1 mM EDTA, 1 mM DTT, and 0.05 mg/mL BSA). To outcompete binding between TF and labeled DNA, 1 pmol (10x molar excess) of unlabeled DNA probe (referred to as “cold probe”) was added where indicated. The solutions were incubated for 15 min at RT, then supplemented with 1x loading dye (0.1 M Tris-HCl, 10 % Glycerol, and 0.01 % Bromophenol blue (Sigma)) and loaded onto the gel. After electrophoresis, gels were imaged using an Amersham Typhoon scanner (Cytiva).

Z-DNA EMSAs were performed by incubating 1 pmol Cy5-labeled, hemimethylated Z-DNA probe (IDT, Supplementary Data 7) in 0.125 mM hexaaminecobalt(III) chloride [(NH_3_)_6_Co]Cl_3_ in H2O for 30 min at RT to allow a B-Z transition, where indicated. After the first incubation step, 500 ng anti-Z-DNA/Z-RNA antibody Z22 (Absolute Antibody cat. No. Ab00783-3.0) was added to the solution in either 1x binding buffer (as described above, poly-dIdC replaced with 10 pmol uLib) or “no salt” buffer (10 mM Tris-HCl, 1 mM DTT, 0.5 % NP40, 0.05 mg/mL BSA and 10 pmol uLib) and incubated for another 30 min at RT. Subsequently, mixes were supplemented with 1x loading dye and run on 6% TBE gels in 0.5% TBE buffer at RT and 100 V.

#### CD spectroscopy

CD spectroscopy measurements were conducted on a Chirascan V100 (AppliedPhotophysics) using 10 μM DNA probes (Merck) in 270 μl (Supplementary Data 7) CD buffer (15 mM NaCl, 10 mM Tris-HCl) with and without hexaaminecobalt(III) chloride (1 mM) (Sigma). DNA probes were incubated at 37 °C for 2 hours to allow a potential B-Z transition. CD measurements were acquired between 230 and 320 nm with a bandwidth of 1 nm, and intervals averaged over 0.5 s at 25 °C.

### Data analysis

#### SMiLE-seq analysis

Sequenced reads were filtered and demultiplexed using custom Python scripts available on GitHub (https://github.com/DeplanckeLab/meSMiLEseq) (version 3.9.5) and pandas (version 2.0.3)^74,75^. To test for enrichment of DNA motifs, the random stretches from both input and eluted libraries were split into *k*-mers (by default, 6, 7, 8, and 9-mers). For each TF, *k*-mers from its corresponding input and eluted fractions were counted separately, and significantly enriched *k*-mers in the eluted library were identified using a right-tailed, one-sided Fisher’s exact test. Calculated p-values were corrected for multiple testing via the Benjamini-Hochberg method, where the number of tests corresponded to the number of unique *k*-mers in the dataset (*p*-value threshold 0.05). Raw sequencing reads without significantly enriched *k*-mers were filtered out; de novo motif discovery was performed with the ProBound Suite^24^ as described below. All calculations were performed using NumPy (version 1.26.4) and SciPy (version 1.13.1)^76,77^.

#### meSMiLE-seq analysis

Data were processed and analyzed as described above (SMiLE-seq analysis) for both uLib and mLib data to assess enrichment of unmethylated and methylated *k*-mers. Scatterplots were created using the ratios of respective *k*-mers (eluted count/ input count), considering their methylation status unless otherwise stated. Empirical cumulative distribution functions (ECDFs) were plotted using Z-transformed *k*-mer counts from the input libraries. Graphs were plotted using matplotlib (version 3.6.2)^78^.

Motif similarities were assessed by flattening both PSAMs and PFMs into one-dimensional arrays. Pearson correlation coefficients (PCC) were then calculated between these flattened vectors for each TF pair. For motifs of unequal lengths, the shorter motif was used as a sliding window across the longer motif, and all possible PCCs were computed. The most extreme PCC value (either closest to 1 (indicating high correlation) or −1 (indicating high anticorrelation)) was reported.

DNA motifs were visualized using the Python package logomaker (version 0.8)^79^.

#### ProBound analysis

SMiLE-seq and meSMiLE-seq data were passed to the ProBound Suite^24^ for de novo motif discovery using the same optimizer settings that were used in the collaborative motif discovery benchmarking study^21^: i.e., the lambdaL2 parameter was set to 0.000001, Dirichlet regularizer weight to 20, and the likelihoodThreshold parameter to 0.000218. meSMiLE-seq data, i.e., uLib and mLib combined, were analyzed using ProBound’s methylation-aware binding models with an extended alphabet, where “mg” and “CG“ indicate methylated and “naked” CG dinucleotides, respectively. The “g” in “mg” also indicates methylation on the complementary DNA strand. Data were given to ProBound as full sequences, with the input libraries serving as background. For each TF, the model was configured to test up to three binding modes, as defined in the original ProBound framework^24^. These modes correspond to potentially distinct TF-DNA interactions, including non-specific binding, dimeric interactions, or potential contributions from co-factors. Where applicable, secondary binding modes are reported as “binding site 2, etc.” Motif discovery was conducted using seed lengths of 6, 9, 12, 15, and 24 base pairs to account for the wide range of DNA-binding domain types, including longer recognition sites commonly associated with C2H2 ZNFs.

Additionally, to obtain clear, canonical DNA binding logos without the extended alphabet, meSMiLE-seq data were split by methylation status according to their molecular barcode (mBC) and analyzed with standard nucleotide encoding. The same aforementioned ProBound optimizer settings, binding modes, and motif seed lengths were applied.

To compare ddG/PSAM models to PFMs from other datasets, ddGs/PSAMs were converted into PFMs as described in Supplementary Fig. 4a. To extract “methyl plus” motifs, ddGs/PSAMs were manually adapted by swapping values of “mg” dinucleotides with “CGs” at respective positions. Similarly, for “methyl minus” or “no CG” motifs values for “m” and “g” nucleotides were not considered when converting ddGs/PSAMs into PFMs.

#### HOMER analysis

Data for KRAB-ZNFs that were not part of the Codebook/GRECO-BIT consortium data (Geo accession number GSE78099)^30^ (Supplementary Data 3) were analyzed by calling “findmotifsGenome.pl” using standard settings, hg19 as reference genome, and a search window of 200 bp.

### TF classification

TFs were classified based on ProBound-generated joint PSAM models, where letter sizes represent the impact of a nucleotide on the stability of the TF-DNA interaction. A TF was classified as methyl minus if a canonical “CG” dinucleotide was present at a given position and the contributions of both C and G exceeded 10% of the Euclidean norm of the PSAM columns. Vice versa, a TF was classified as methyl plus if “mg” was present in place of “CG” under the same criterion. If both “CG” and “mg” were present at the same position, a CG/mg ratio was calculated; ratios differing by 10% or more determined classification into the respective group. TFs not meeting these conditions were labeled as “little effect/no CG dinucleotide”.

Additionally, this classification was confirmed by comparing independently inferred PSAM logos from uLib and mLib. Core motif positions were defined as those with Kullback-Leibler (KL) divergence from the background nucleotide distribution (estimated from input libraries) exceeding 0.1:

1 $${D}_{{KL}}\left(P\parallel Q\right)=\mathop{\sum }\limits_{i}P\left(i\right){\log }_{2}\frac{P\left(i\right)}{Q\left(i\right)}$$

where $P\left(i\right)$ is the nucleotide probability at position i in the motif, and $Q\left(i\right)$ is the background probability. Divergent positions between uLib and mLib motifs were identified using Jensen-Shannon (JS) divergence with a threshold of 0.01, as described in ref. ^80^:

2 $${D}_{{JS}}\left({P|Q}\right)=\frac{1}{2}{D}_{{KL}}\left({P|M}\right)+\frac{1}{2}{D}_{{KL}}\left({Q|M}\right),M=\frac{P+Q}{2}$$

For each divergent position, all possible consensus variants were enumerated, and exact matches were quantified in the input-normalized data for both libraries. Ratios of mLib to uLib >1 indicated preferential enrichment in mLib, whereas ratios <1 indicated enrichment in uLib. Positions containing C or G within a CG context were considered putative methylation-sensitive sites.

### Motif occurrences in ChIP-seq and ChIP-exo data and their methylation status

All ChIP-seq and ChIP-exo datasets were mapped to or lifted over to the reference genome hg19. Sequences were extracted from TF-specific datasets (Supplementary Data 3) by calling “bedtools getfasta”^81^. Methylation-sensitive PSAM models were transformed into PFMs in MEME format as described above, and all matrices were elongated by the 0-order background model used in the FIMO tool from the MEME suite to have a standard size of 20 bp^27,82^. To find individual motif occurrences within TF-specific peaks, PFMs were applied using FIMO with default parameters (*p*-value threshold <10–4). The resulting file “best_site.narrowPeak” was intersected with publicly available WGBS data for HEK293/T cells^35,36^ (Supplementary Data 3) via “bedtools intersect”^81^ to obtain information about DNA methylation. Motif-specific methylation levels were compared to overall CG methylation levels of ChIP-seq or ChIP-exo peaks containing those motifs. WGBS data were filtered to exclude low-coverage loci, ensuring the recommended minimum average coverage of 15x across analyzed regions^83^. To assess whether motif-associated methylation patterns differed significantly from genomic background, we constructed a background methylation distribution by randomly selecting 29,076 genomic regions (the median number of ChIP-seq peaks used in this study), each 463 bp in length (average peak size) using “bedtools random”^81^. These regions were equally intersected with WGBS data to compute background methylation levels. Statistical significance was assessed using a Kolmogorov-Smirnov test comparing motif-specific and background methylation distributions.

In addition, genomic loci were intersected with ChromHMM tracks for HEK293/T cells^38^ to extract motif-specific chromatin annotations. Datasets for “methyl plus” TFs were split based on methylation levels, comparing motif occurrences being at least 50% methylated to those below the threshold with adjusted number of reads. These TFBS containing regions were used for methylation-specific gene ontology (GO) enrichment using Enrichr within a 3 kb distance of the motif^84–86^.

### Calculating ChIP-seq associations for ZHX2

Permutation-based global associations between ChIP-seq tracks (Supplementary Data 3) were calculated with the regioneReloaded package in R (version 4.3.1)^87^ using the “crosswisePermTest” function with the settings “resampleGenome”, ntimes = 1000, evFUN = “numOverlaps”. Associations were quantified as:

3 $${Z}_{s}=\frac{{O}_{{actual}}-{\mu }_{{random}}}{\sqrt{n}}$$

where ${O}_{{actual}}$ is the number of overlaps between genomic regions, and ${\mu }_{{random}}$ is the mean of overlaps by randomly shuffling peak sets n times. Local associations were calculated with the “multiLocalZscore” function with the same settings mentioned above, a sliding window of 7500 bp, and a step size of 100 bp.

### Reporting summary

Further information on research design is available in the Nature Portfolio Reporting Summary linked to this article.

## Supplementary information

Supplementary Information

Transparent Peer Review file

Reporting Summary

Description of Additional Supplementary Files

Supplementary Data 1

Supplementary Data 2

Supplementary Data 3

Supplementary Data 4

Supplementary Data 5

Supplementary Data 6

Supplementary Data 7

## Source data

Source Data

## Data Availability

Raw SMiLE-seq sequencing datasets were deposited on ArrayExpress under accession code E-MTAB-14597 (meSMiLE-seq) and E-MTAB-14598 (SMiLE-seq). A comprehensive list of data used in this study is available in Supplementary Data 3. DNA motifs can be browsed at mex.autosome.org, https://cisbp.ccbr.utoronto.ca/, and are available in Supplementary Data 4. An updated list of human TFs is available at https://humantfs.ccbr.utoronto.ca/. Information on constructs, experiments, analyses, processed data, comparison tracks, and browsable pages with information and results for each TF is available at codebook.ccbr.utoronto.ca. DNA and AA sequences of TFs that were used in this study are also available in Supplementary Data 1. Source data are provided with this paper.

## Code availability

Custom analysis pipelines and PSAMs with logos used in this manuscript have been deposited on ZENODO^88^ and are available on GitHub (https://github.com/DeplanckeLab/meSMiLEseq).
