# Supplementary material for: Identification of methylation-sensitive human transcription factors using meSMiLE-seq
Source: Nat Commun. 2026 Aug 5;17:7771. doi: 10.1038/s41467-026-71387-y (PMC13444138; doi:10.1038/s41467-026-71387-y)
Supplement: Supplementary file 2 [file 41467_2026_71387_MOESM1_ESM.pdf]

# Identification of methylation-sensitive human transcription factors using meSMiLE-seq

***Supplementary Information***

Antoni J. Gralak[1,2], Katerina Faltejskova[3,4], Ally W.H. Yang[5], Clemence Steiner[1], Julie Russeil[1,2], Nadia Grenningloh[1], Sachi Inukai[1,27], Mustafa Demir[1,28], Riccardo Dainese[1,28], Cooper Owen[1], Eugenia V. Pankevich[1], Codebook/GRECO-BIT Consortium[+], Timothy R. Hughes[5], Ivan V. Kulakovskiy[6,7,8], Judith F. Kribelbauer-Swietek[1,2,9], Guido van Mierlo[1,2,10], Bart Deplancke[1,2]*

**Supplementary Figures 1-8**

**Supplementary Data 1-7**

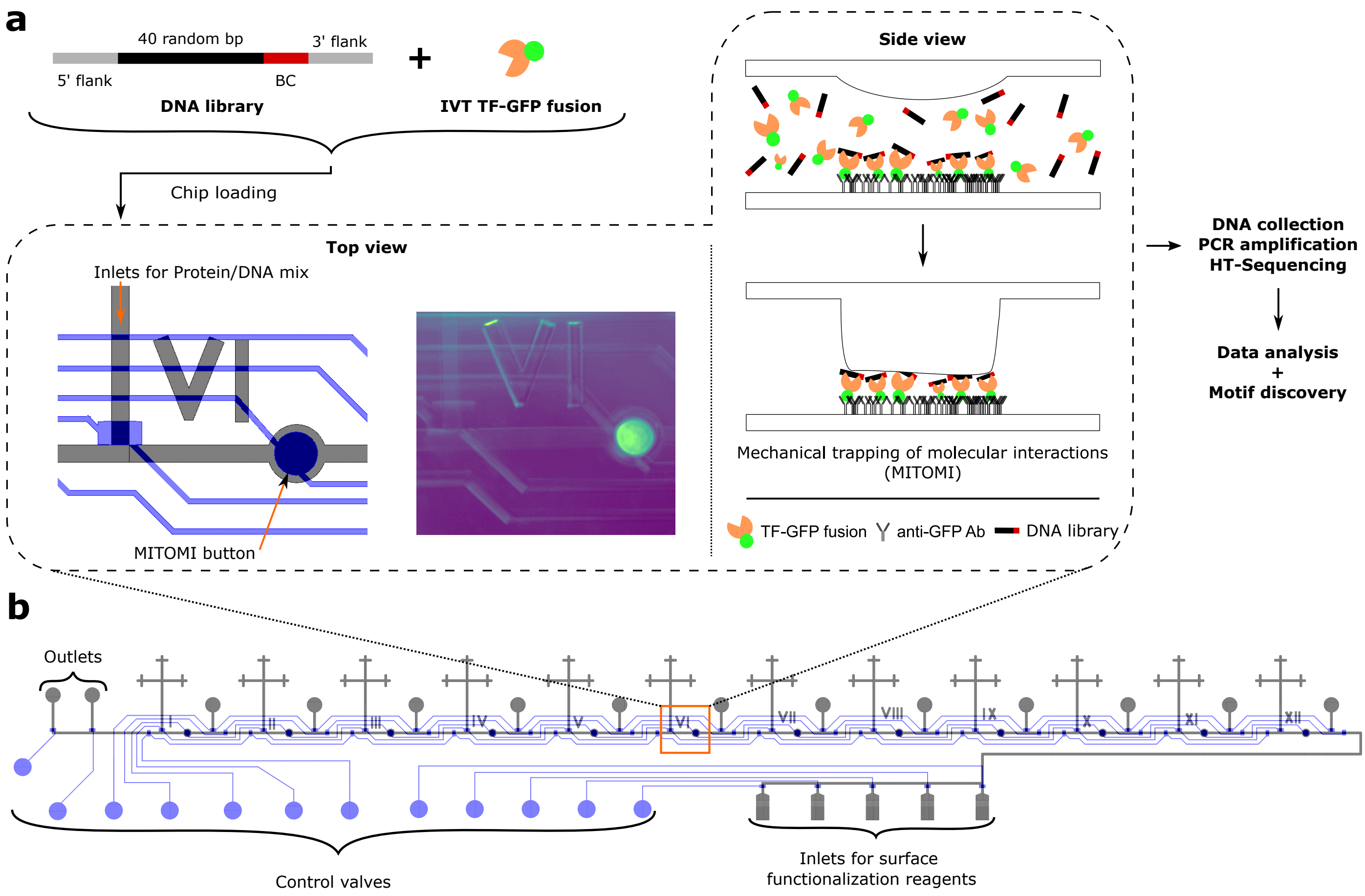


**Supplementary Figure 1.**

**a)** Illustration of the SMiLE-seq workflow with schematic description of DNA libraries used in classical SMiLE-seq experiments. BC: Barcode, IVT: *in vitro* transcription and translation. **b)** Schematic structure of a SMiLE-seq chip, the control layer is depicted in blue, the flow layer in black. Each chip contains 12 MITOMI buttons allowing for 12 separate experiments per assay.

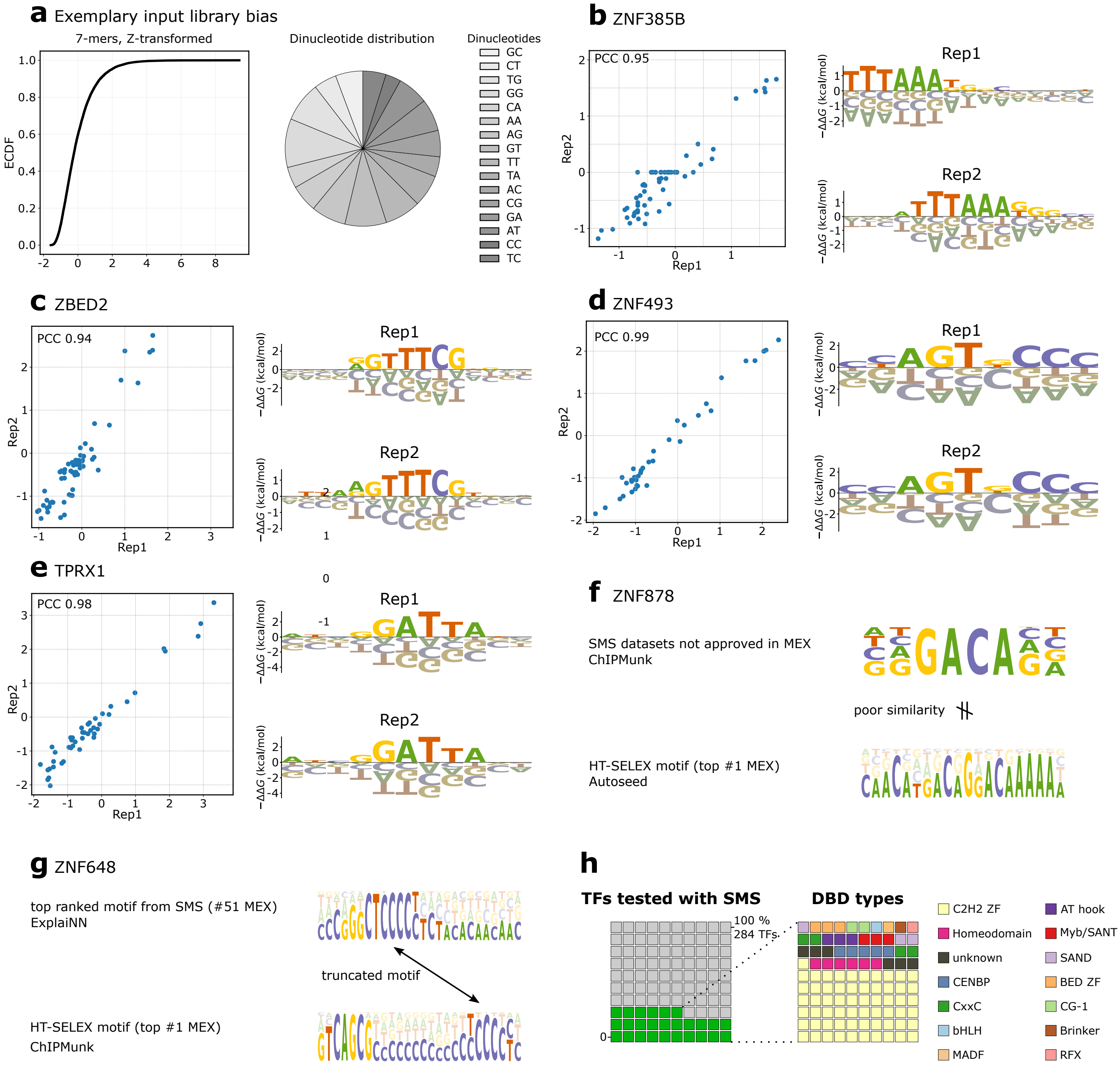


**Supplementary Figure 2.**

**a)** Empirical cumulative distribution function (ECDF) of 7-mers and the dinucleotide distribution of an exemplary input library used in classical SMiLE-seq. **b) to e)** Correlation scatterplots of flattened, ProBound generated PSAMs for ZNF385B, ZBED2, ZNF493, and TPRX1. PCC: Pearson correlation coefficient. **f) and g)** PFMs inferred from SMiLE-seq data for ZNF878 and ZNF648 using non-customized standard motif discovery tools, compared to top-ranked motifs in the collaborator's study[21]. In standard pipelines, synthesis-related nucleotide biases in the DNA libraries are insufficiently corrected, leading to hallucinated (**f**) or truncated motifs (**g**). By contrast, analysis of the same SMiLE-seq datasets without our approach recovered binding motifs closely resembling those from the collaborator's study (see **Figure 1c** and **d**). **h)** Waffle chart (10 x 10 grid, one square represents 1%) represents the distribution of DNA-binding domain (DBD) types among TFs that yielded DNA motifs in this study. ***Left***: proportion of assayed TFs that yielded motifs are depicted in green (see **Figure 1b**). ***Right***: Zoom-in of the highlighted portion (26% of TFs), subdivided by DBD type.

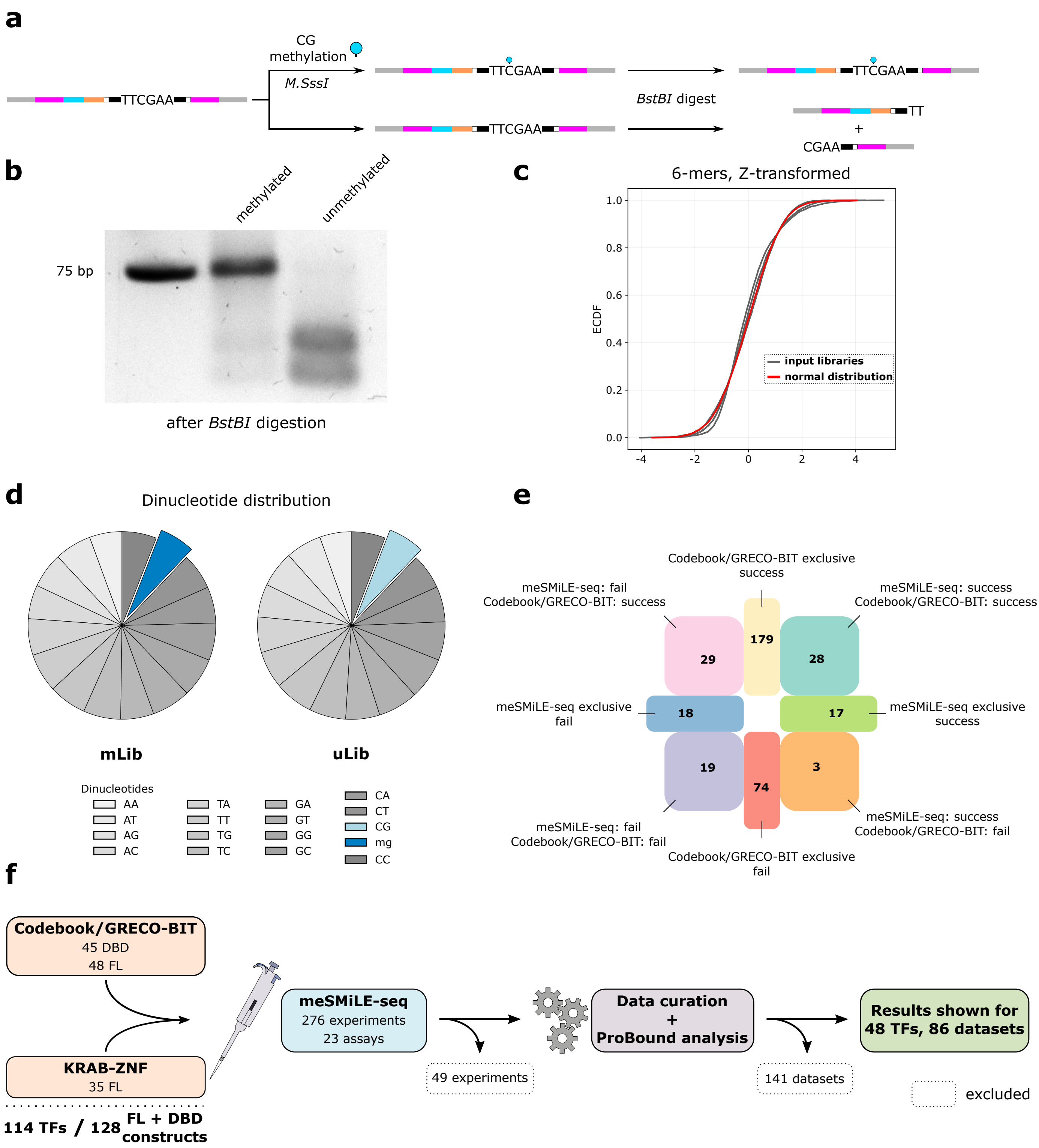


**Supplementary Figure 3.**

**a)** Control library designed to optimize the methylation reaction. The structure is equivalent to other meSMiLE-seq libraries, with an additional cleavage site for the methylation-sensitive restriction enzyme *BstBI* within the random region. **b)** Agarose gel electrophoresis of methylated and unmethylated control libraries after *BstBI* digest. **c) and d)** ECDF of 6-mers and the dinucleotide distribution of newly designed DNA libraries used in meSMiLE-seq. **e)** Overlap of TFs assayed in meSMiLE-seq and the Codebook/GRECO-BIT collaboration. Numbers indicate the number of TFs that belong to the reported description. For example, 'meSMiLE-seq exclusive success/fail' indicates the number of TFs that were assayed solely via meSMiLE-seq and not in the collaboration, out of which 17 yielded DNA motifs and 18 did not. **f)** Schematic overview of how many TFs were assayed with meSMiLE-seq and for how many TFs, DNA binding motifs were approved.

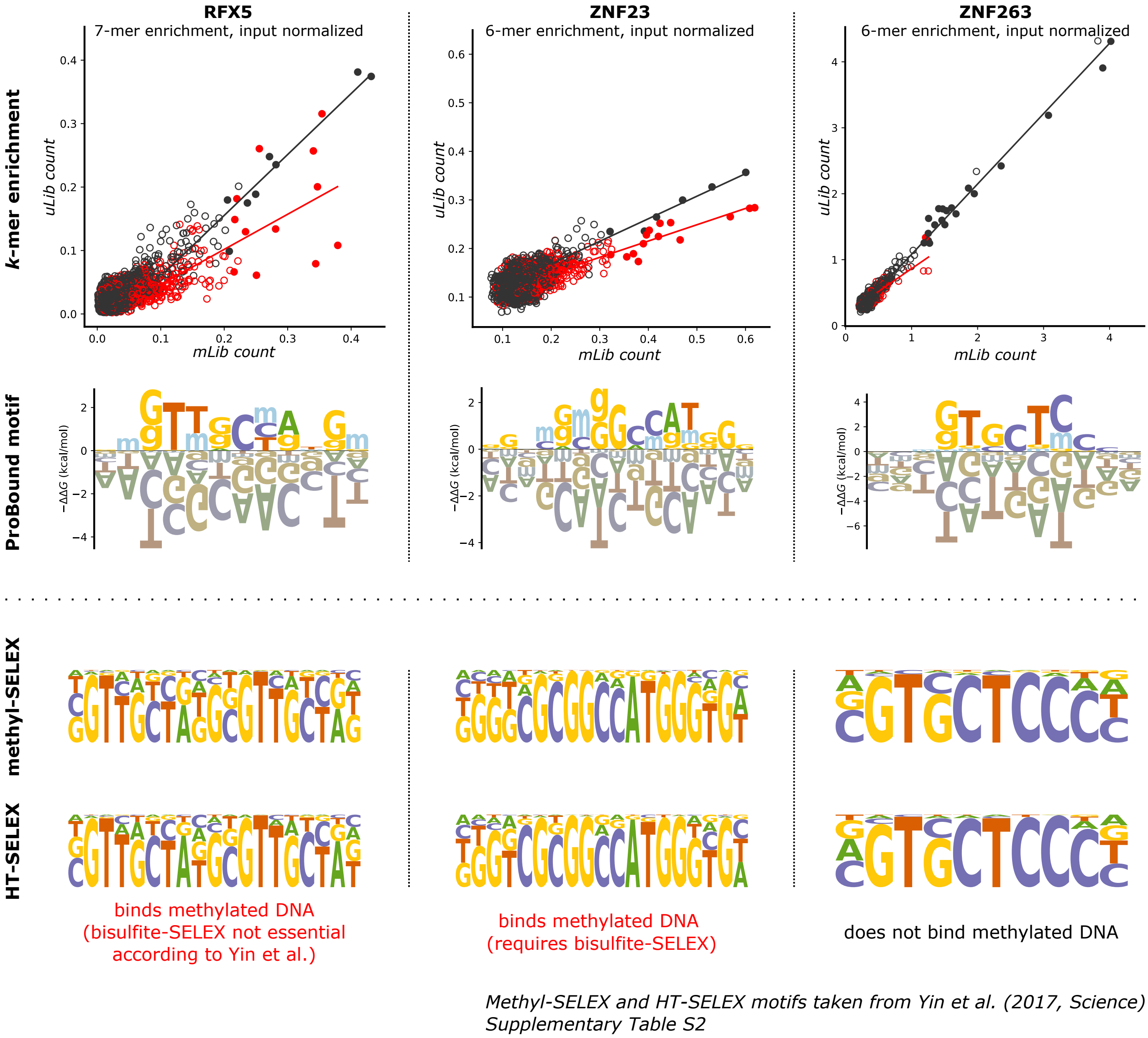


**Supplementary Figure 4.**

***Top:*** meSMiLE-seq-derived DNA binding sites and corresponding *k*-mer scatterplots for three positive controls: RFX5 and ZNF23 as methylation-dependent binders, and ZNF263 as a methylation-independent TF. ***Bottom****:* DNA logos and classification for the same TFs predicted by methyl-SELEX and HT-SELEX (data derived from[12]). meSMiLE-seq correctly recapitulates the previously reported DNA-binding models.

**a** *HT-SELEX and methyl-SELEX processes*

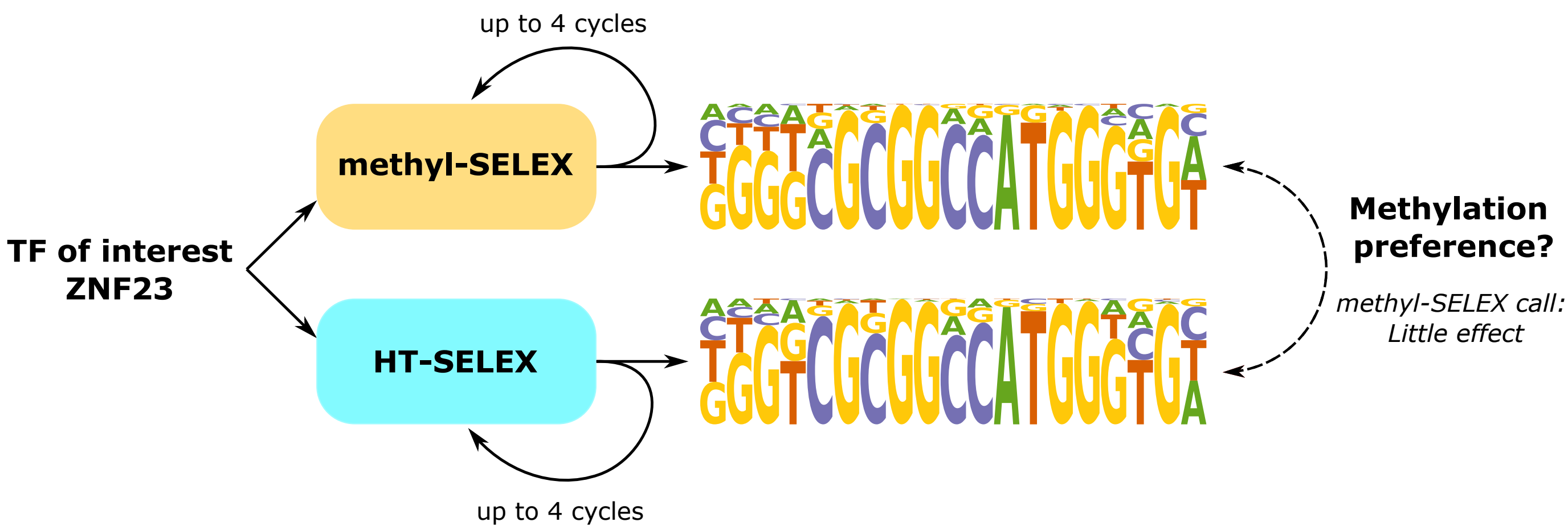


**b** *bisulfite-SELEX process*

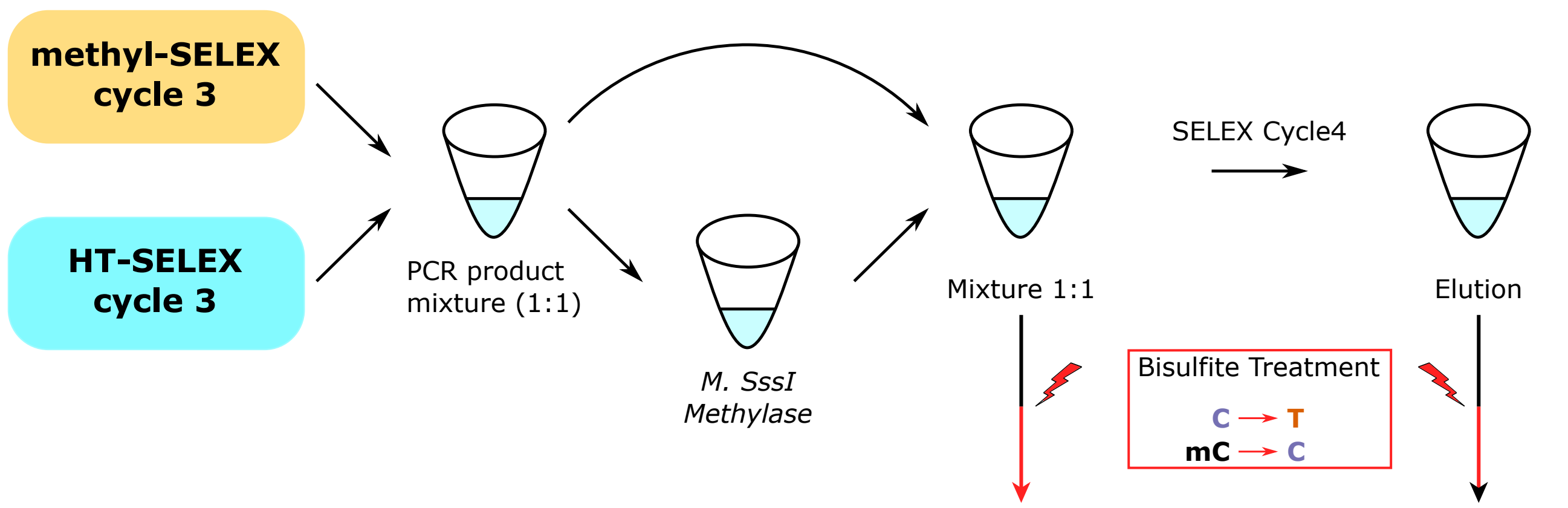


| Sequence | mCG enrichment | cycle3 HTS | | cycle3 BS | | cycle4 HTS | | cycle4 BS | |
|---|---|---|---|---|---|---|---|---|---|
| | | %CG | %TG | %CG | %TG | %CG | %TG | %CG | %TG |
| NNN**YG**CGGCCATGGGCGN | 36.33% | 0.67 | 0.01 | 0.34 | 0.34 | 0.82 | 0.00 | 0.55 | 0.22 |
| NNNCG**YG**GCCATGGGCGN | 40.75% | 0.97 | 0.00 | 0.51 | 0.46 | 1.00 | 0.00 | 0.74 | 0.26 |
| NNNCGCGGCCATGGG**YG**N | 29.63% | 0.16 | 0.46 | 0.09 | 0.63 | 0.15 | 0.61 | 0.10 | 0.72 |

*bisulfite-SELEX call:*
*MethylPlus*

*Figure adapted from Yin et al. (2017, Science)*
*Figure 4 and Supplementary Tables S2 and S3*

**c** *meSMiLE-seq*

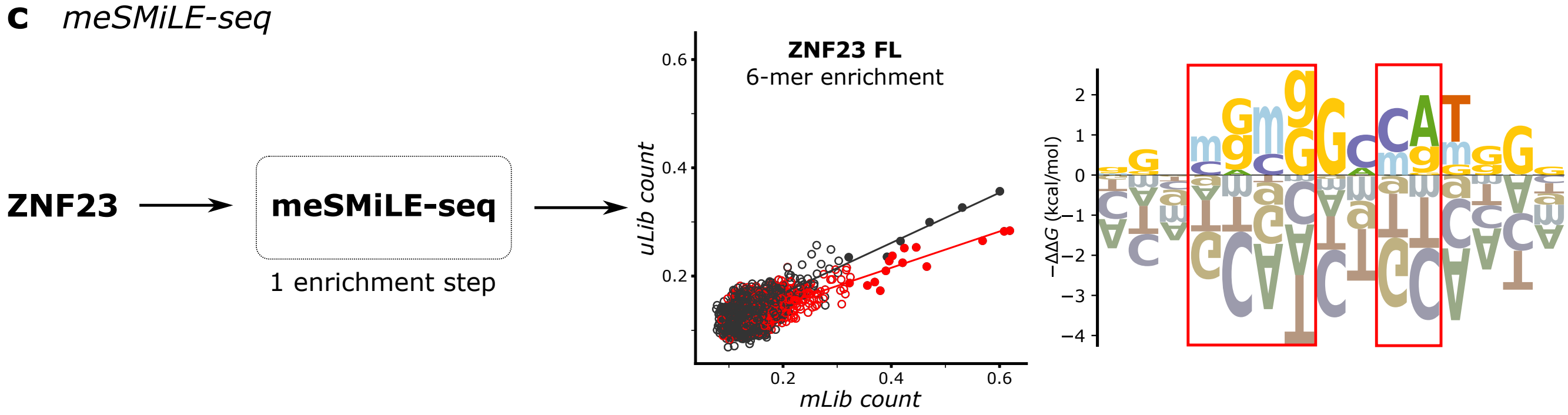


| Sequence GGG**CGCG**GC**CA**TGGG | ratio %mg/%CG | mLib %mg | mLib count⁺ | uLib %CG | uLib count⁺ |
|---|---|---|---|---|---|
| -GG**NG**CGG------- | 1.20 | 43.9 | 1.14 | 36.5 | 0.51 |
| --GCG**NG**GC------ | 1.40 | 55.9 | 1.16 | 39.8 | 0.56 |
| -------GC**CN**TGG- | 1.06 | 18.6 | 0.59 | 17.6 | 0.35 |

+input normalized

*meSMiLE-seq call:*
*MethylPlus*

**Supplementary Figure 5.**

**a)** HT-SELEX and methyl-SELEX workflow[12]: a TF of interest is assayed separately with methylated or unmethylated DNA through up to four enrichment cycles. Because motifs are inferred independently, methylation preferences can be difficult to resolve, as illustrated with ZNF23. **b)** For correct prediction, an additional bisulfite-SELEX step is required. In short, enriched DNA from methyl- and HT-SELEX is mixed equimolarly and subjected to two bisulfite conversions. CG/TG dinucleotide occurrences are then used to calculate enrichment and infer methylation preference. **c)** In contrast, meSMiLE-seq resolves the same methylation preference in a single experiment with one enrichment step and no bisulfite conversion.

**a** ddG to pfm conversion

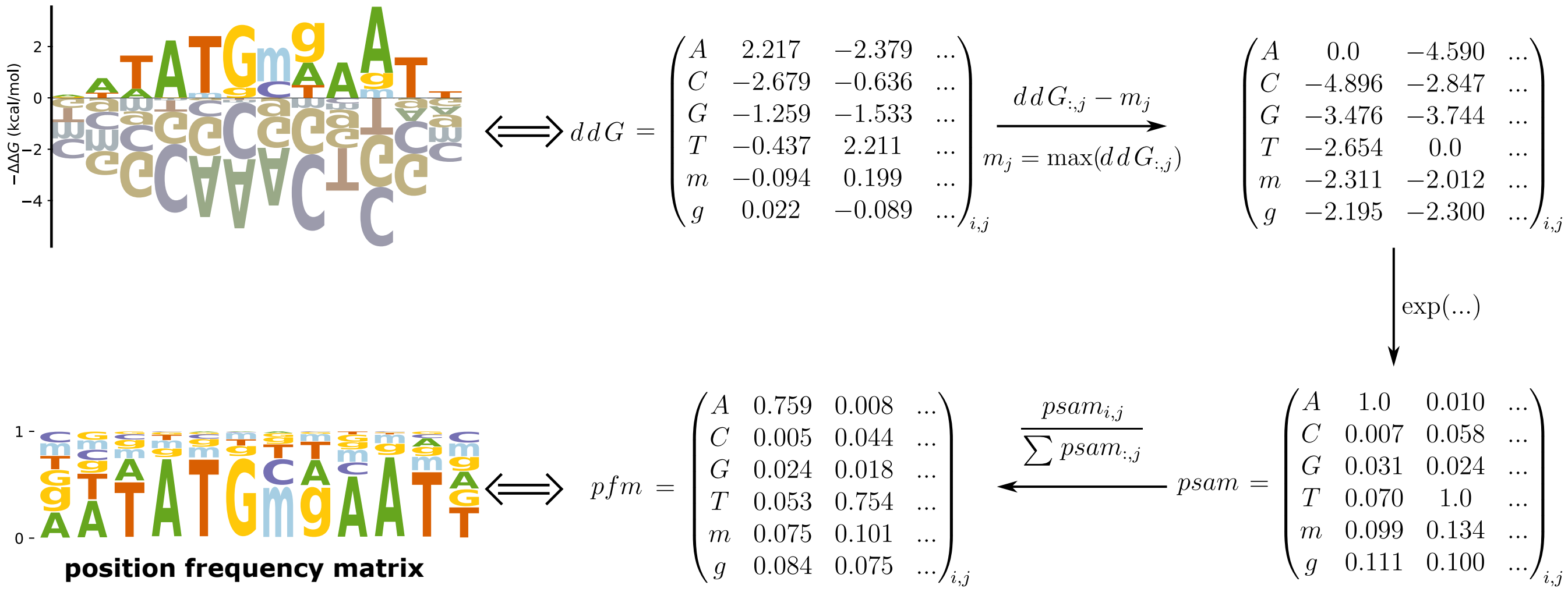


**b** ZNF445

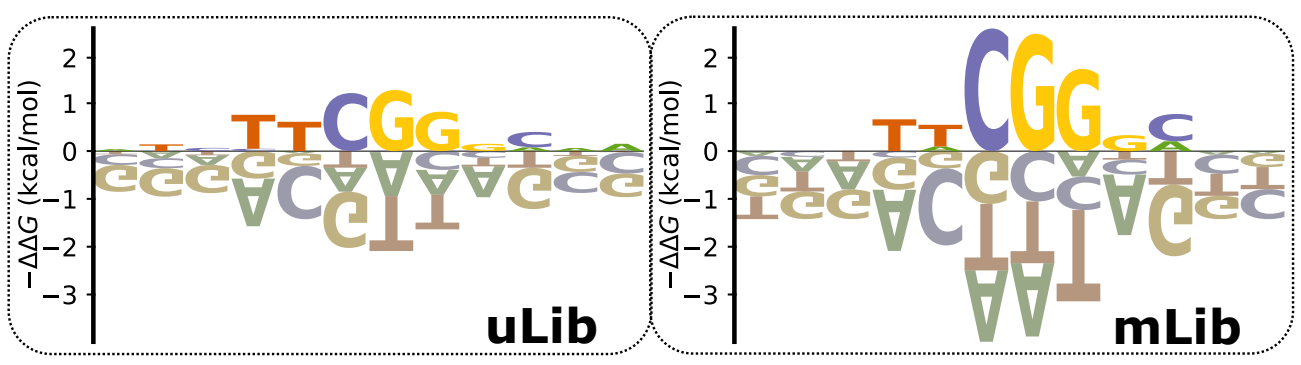


| Consensus | ratio $\frac{\%mLib}{\%uLib}$ | mLib %CG | mLib count$^+$ | uLib %CG | uLib count$^+$ |
|---|---|---|---|---|---|
| CTT**NG**GGC | 2.19 | 80.3 | 5.00 | 36.6 | 0.91 |

$^+$*input normalized*

**d** USF3

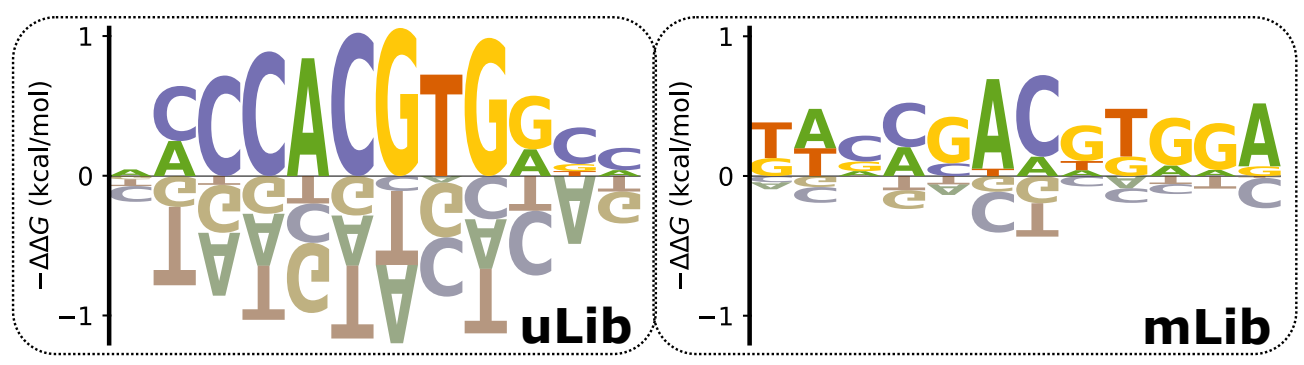


| Consensus | ratio $\frac{\%mLib}{\%uLib}$ | mLib %CG | mLib count$^+$ | uLib %CG | uLib count$^+$ |
|---|---|---|---|---|---|
| CCA**NG**TG | 0.89 | 87.2 | 69.0 | 98.4 | 269.7 |

$^+$*input normalized*

**c** PRDM13

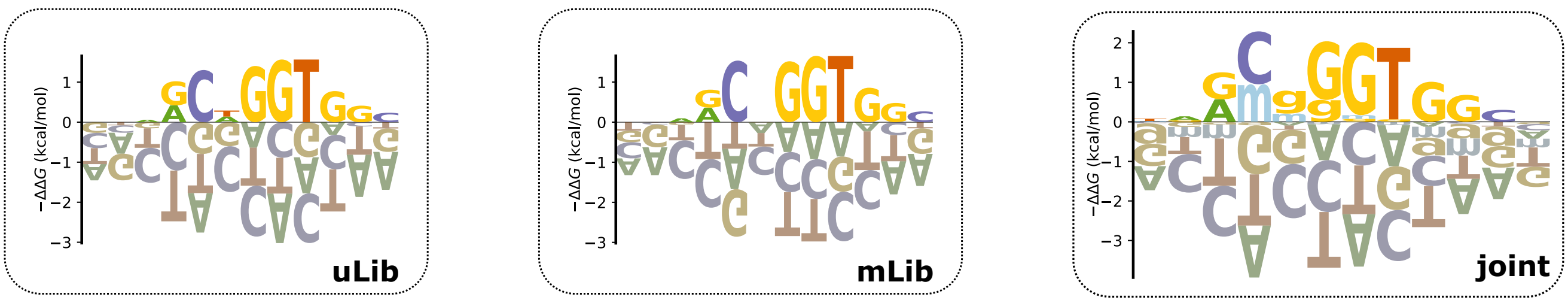


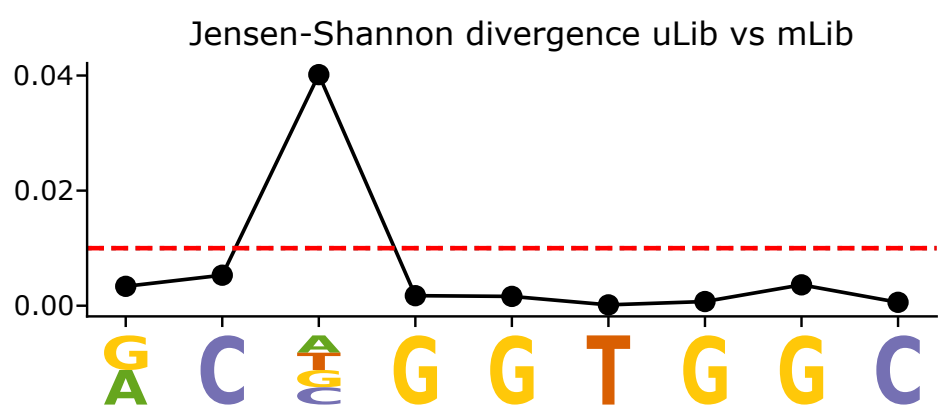


| ↓ div. position GC**N**GGTGGC | ratio $\frac{\%mLib}{\%uLib}$ | mLib % | mLib count$^+$ | uLib % | uLib count$^+$ |
|---|---|---|---|---|---|
| GC**G**GGTGGC | 2.90 | 45.8 | 2.08 | 15.8 | 0.53 |
| GC**T**GGTGGC | 0.58 | 18.3 | 0.83 | 31.5 | 1.06 |
| GC**A**GGTGGC | 0.46 | 20.2 | 0.92 | 43.9 | 1.47 |
| GC**C**GGTGGC | 1.80 | 15.8 | 0.72 | 8.8 | 0.30 |

$^+$*input normalized*

**Supplementary Figure 6.**

**a**) Schematic showing how a ddG matrix is transformed into PFMs. To exclude information about methylation sensitivity, the ‘m’ and ‘g’ rows are omitted. **b)** Independent uLib and mLib motifs of ZNF445 enrich the same DNA binding site, with the mLib motif indicating stronger binding and resembling the joint motif (see **Figure 4a**). The sequence ‘CTTCGGGC’ is markedly more enriched in mLib than in uLib. **c)** For PRDM13, mLib and uLib motifs diverge at the 3rd position, predicted as a methylation-sensitive site in the joint motif. Indeed, ‘GCGGGTGGC’ makes up 45.8% of counts in mLib versus only 15.8% in uLib. **d)** In contrast, the USF3 consensus sequence ‘CCACGTG’ is more enriched in uLib than in mLib, both in relative percentages (98.4 % vs. 87.2 %) and in absolute, input-normalized counts (269.7 vs 69.0).

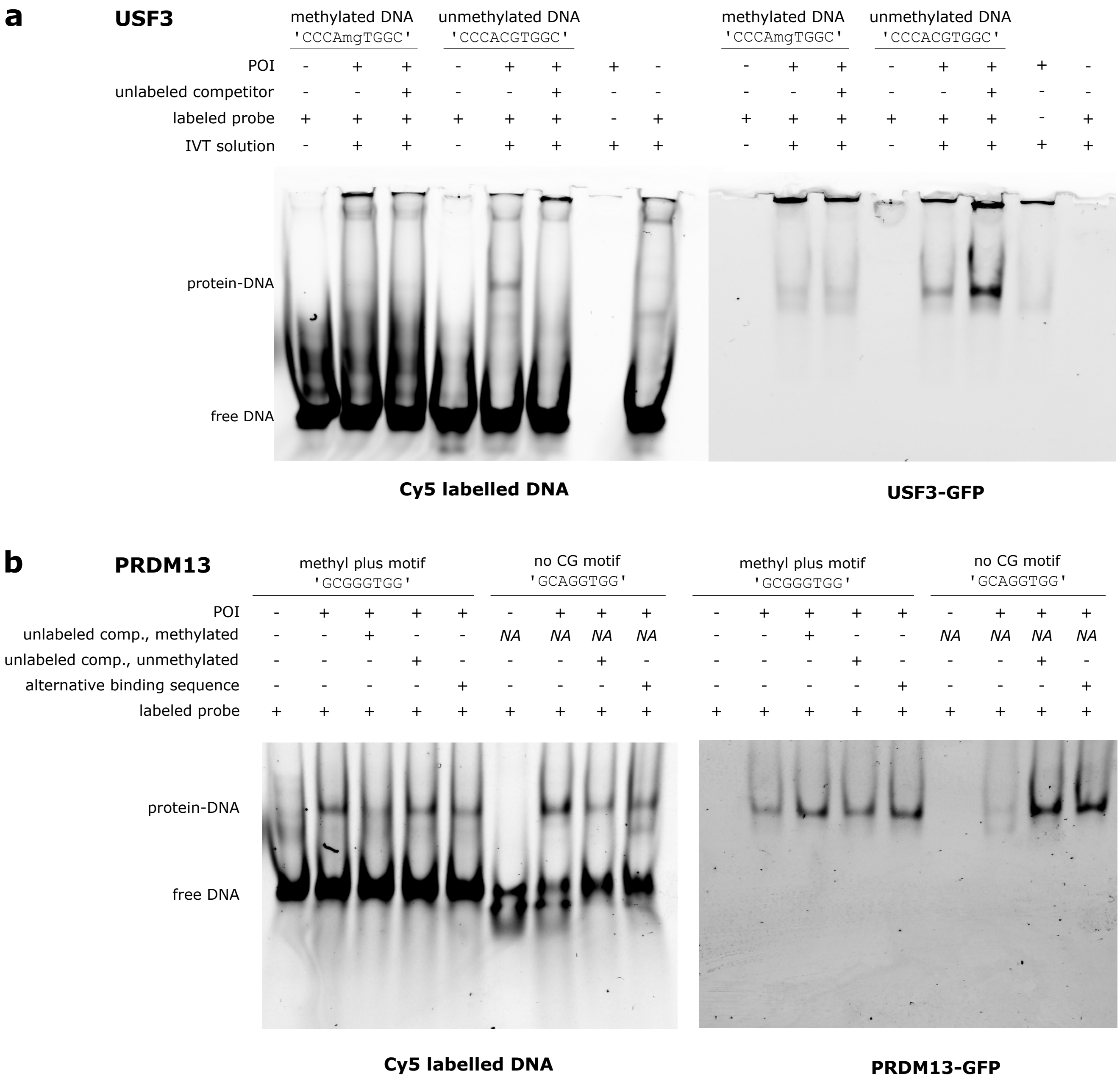


**Supplementary Figure 7.**

EMSA validation of methylation-sensitive DNA binding for USF3 ('methyl minus') (**a**) and for PRDM13 ('methyl plus') (**b**). ***Left***: signal corresponds to Cy5-labelled DNA probes; ***right:*** signal corresponds to GFP as TFs were expressed as TF-GFP fusion proteins. POI: protein of interest, 'cold, methyl.': methylated cold probe, 'cold, unmethyl.': unmethylated cold probe, alternative bs.: alternative binding site (applicable for PRDM13). EMSAs shown are from single independent experiments.

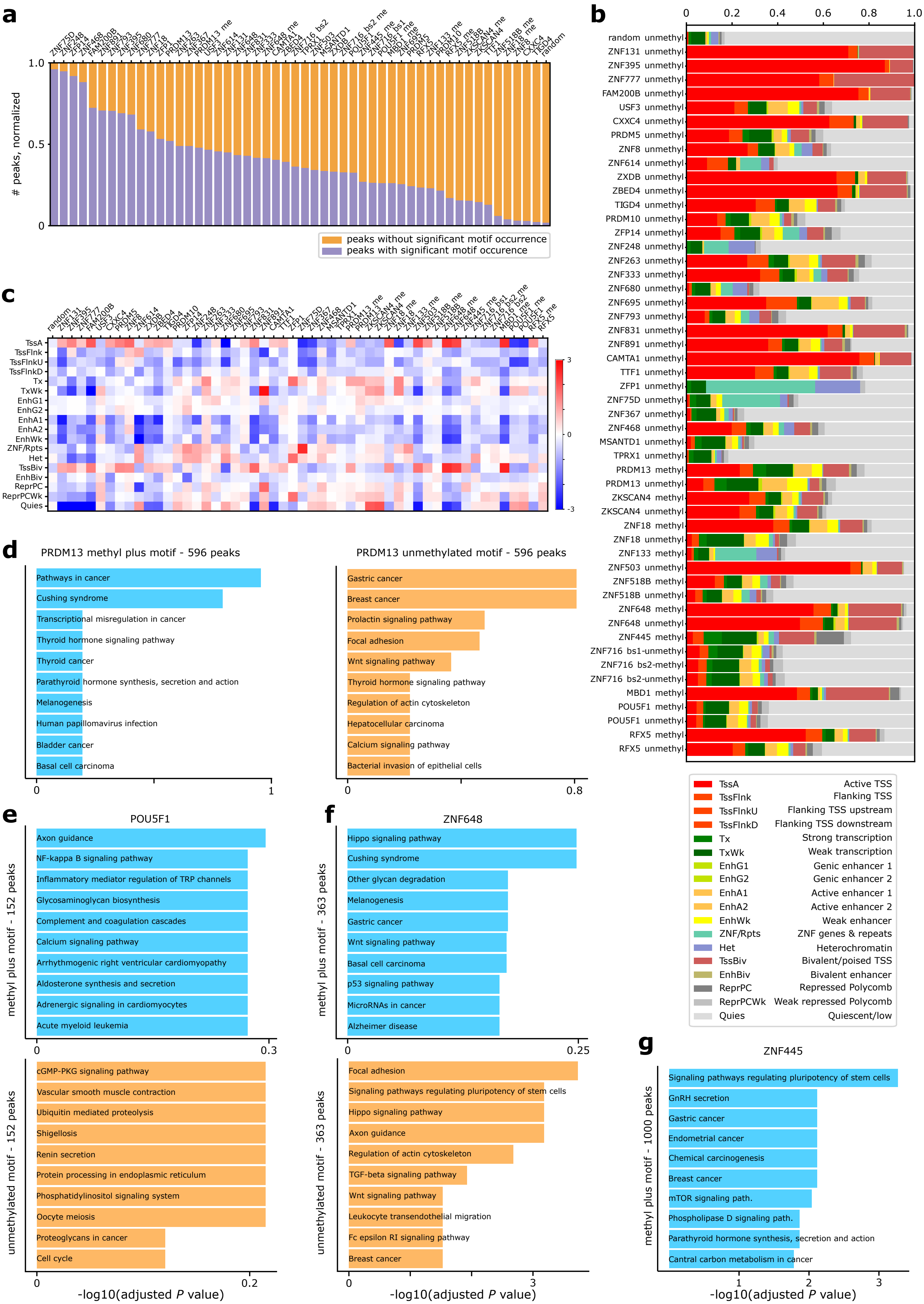

a
1.0
0.5
0
# peaks, normalized
peaks without significant motif occurrence
peaks with significant motif occurence
b
0
0.2
0.4
0.6
0.8
1.0
random unmethyl
ZNF131 unmethyl
ZNF395 unmethyl
ZNF777 unmethyl
FAM200B unmethyl
USF3 unmethyl
CXXC4 unmethyl
PRDM5 unmethyl
ZNF8 unmethyl
ZNF614 unmethyl
ZXDB unmethyl
ZBED4 unmethyl
TIGD4 unmethyl
PRDM10 unmethyl
ZFP14 unmethyl
ZNF248 unmethyl
ZNF263 unmethyl
ZNF333 unmethyl
ZNF680 unmethyl
ZNF695 unmethyl
ZNF793 unmethyl
ZNF831 unmethyl
ZNF891 unmethyl
CAMTA1 unmethyl
TTF1 unmethyl
ZFP1 unmethyl
ZNF75D unmethyl
ZNF367 unmethyl
ZNF468 unmethyl
MSANTD1 unmethyl
TPRX1 unmethyl
PRDM13 methyl
PRDM13 unmethyl
ZKSCAN4 methyl
ZKSCAN4 unmethyl
ZNF18 methyl
ZNF18 unmethyl
ZNF133 methyl
ZNF503 unmethyl
ZNF518B methyl
ZNF518B unmethyl
ZNF648 methyl
ZNF648 unmethyl
ZNF445 methyl
ZNF716 bs1-unmethyl
ZNF716 bs2-methyl
ZNF716 bs2-unmethyl
MBD1 methyl
POU5F1 methyl
POU5F1 unmethyl
RFX5 methyl
RFX5 unmethyl
TssA Active TSS
TssFlnk Flanking TSS
TssFlnkU Flanking TSS upstream
TssFlnkD Flanking TSS downstream
Tx Strong transcription
TxWk Weak transcription
EnhG1 Genic enhancer 1
EnhG2 Genic enhancer 2
EnhA1 Active enhancer 1
EnhA2 Active enhancer 2
EnhWk Weak enhancer
ZNF/Rpts ZNF genes & repeats
Het Heterochromatin
TssBiv Bivalent/poised TSS
EnhBiv Bivalent enhancer
ReprPC Repressed Polycomb
ReprPCWk Weak repressed Polycomb
Quies Quiescent/low
c
TssA
TssFlnk
TssFlnkU
TssFlnkD
Tx
TxWk
EnhG1
EnhG2
EnhA1
EnhA2
EnhWk
ZNF/Rpts
Het
TssBiv
EnhBiv
ReprPC
ReprPCWk
Quies
3
0
-3
d
PRDM13 methyl plus motif - 596 peaks
Pathways in cancer
Cushing syndrome
Transcriptional misregulation in cancer
Thyroid hormone signaling pathway
Thyroid cancer
Parathyroid hormone synthesis, secretion and action
Melanogenesis
Human papillomavirus infection
Bladder cancer
Basal cell carcinoma
0
1
PRDM13 unmethylated motif - 596 peaks
Gastric cancer
Breast cancer
Prolactin signaling pathway
Focal adhesion
Wnt signaling pathway
Thyroid hormone signaling pathway
Regulation of actin cytoskeleton
Hepatocellular carcinoma
Calcium signaling pathway
Bacterial invasion of epithelial cells
0
0.8
e
POU5F1
methyl plus motif - 152 peaks
Axon guidance
NF-kappa B signaling pathway
Inflammatory mediator regulation of TRP channels
Glycosaminoglycan biosynthesis
Complement and coagulation cascades
Calcium signaling pathway
Arrhythmogenic right ventricular cardiomyopathy
Aldosterone synthesis and secretion
Adrenergic signaling in cardiomyocytes
Acute myeloid leukemia
0
0.3
unmethylated motif - 152 peaks
cGMP-PKG signaling pathway
Vascular smooth muscle contraction
Ubiquitin mediated proteolysis
Shigellosis
Renin secretion
Protein processing in endoplasmic reticulum
Phosphatidylinositol signaling system
Oocyte meiosis
Proteoglycans in cancer
Cell cycle
0
0.2
-log10(adjusted P value)
f
ZNF648
methyl plus motif - 363 peaks
Hippo signaling pathway
Cushing syndrome
Other glycan degradation
Melanogenesis
Gastric cancer
Wnt signaling pathway
Basal cell carcinoma
p53 signaling pathway
MicroRNAs in cancer
Alzheimer disease
0
0.25
unmethylated motif - 363 peaks
Focal adhesion
Signaling pathways regulating pluripotency of stem cells
Hippo signaling pathway
Axon guidance
Regulation of actin cytoskeleton
TGF-beta signaling pathway
Wnt signaling pathway
Leukocyte transendothelial migration
Fc epsilon RI signaling pathway
Breast cancer
0
3
-log10(adjusted P value)
g
ZNF445
methyl plus motif - 1000 peaks
Signaling pathways regulating pluripotency of stem cells
GnRH secretion
Gastric cancer
Endometrial cancer
Chemical carcinogenesis
Breast cancer
mTOR signaling path.
Phospholipase D signaling path.
Parathyroid hormone synthesis, secretion and action
Cantral carbon metabolism in cancer
1
2
3
-log10(adjusted P value)

**Supplementary Figure 8.**

**a)** Bar plot depicts percentages of TF-specific ChIP-seq peaks in which meSMiLE-seq-derived motifs were found, using the method shown in **Figure 5a-b. b) and c)** ChromHMM annotations of individual motif occurrences in TF-specific ChIP-seq peaks for all TFs, depicted as bar charts and as log2-transformed ratios as described in **Figure 5e-f. d) to g)** Gene ontology enrichment analysis of 'methyl plus' TFs PRDM13, POU5F1, ZBF648 and ZNF445. Adjusted P values were calculated with the Fisher's exact test and corrected via the Benjamini-Hochberg method.
