# Supplementary material for: Identification of methylation-sensitive human transcription factors using meSMiLE-seq
Source: Nat Commun. 2026 Aug 5;17:7771. doi: 10.1038/s41467-026-71387-y (PMC13444138; doi:10.1038/s41467-026-71387-y)
Supplement: Supplementary file 3 [file 41467_2026_71387_MOESM2_ESM.pdf]

nature portfolio

Peer Review File

Identification of methylation-sensitive human transcription factors using meSMiLE-seq

Corresponding Author: Professor Bart Deplancke

This manuscript has been previously reviewed at another journal. This document only contains information relating to versions considered at Nature Communications.

This file contains all reviewer reports in order by version, followed by all author rebuttals in order by version.

Version 0:

Reviewer comments:

Reviewer #1

(Remarks to the Author)
The authors have addressed all my previous concerns in a thoughtful and comprehensive manner. I am fully satisfied.

(Remarks on code availability)

Reviewer #3

(Remarks to the Author)
The authors have satisfactorily addressed my prior comments. While the authors are not able to provide or suggest mechanisms for the methylation-sensitive binding specificities that they found by meSMILE-Seq, the results reported in this paper, as well as the description of the meSMILE-Seq assay and data analysis methods, will nevertheless be a valuable resource to the regulatory genomics community.

(Remarks on code availability)
I have not reviewed the code.

Open Access This Peer Review File is licensed under a Creative Commons Attribution 4.0 International License, which permits use, sharing, adaptation, distribution and reproduction in any medium or format, as long as you give appropriate credit to the original author(s) and the source, provide a link to the Creative Commons license, and indicate if changes were made.
In cases where reviewers are anonymous, credit should be given to 'Anonymous Referee' and the source.
The images or other third party material in this Peer Review File are included in the article's Creative Commons license, unless indicated otherwise in a credit line to the material. If material is not included in the article's Creative Commons license and your intended use is not permitted by statutory regulation or exceeds the permitted use, you will need to obtain permission directly from the copyright holder.
To view a copy of this license, visit https://creativecommons.org/licenses/by/4.0/
