# Supplementary material for: Identification of methylation-sensitive human transcription factors using meSMiLE-seq
Source: Nat Commun. 2026 Aug 5;17:7771. doi: 10.1038/s41467-026-71387-y (PMC13444138; doi:10.1038/s41467-026-71387-y)
Supplement: Supplementary file 4 [file 41467_2026_71387_MOESM3_ESM.pdf]

nature portfolio

Corresponding author(s): Bart Deplancke

Last updated by author(s): Mar 3, 2026

Reporting Summary

Nature Portfolio wishes to improve the reproducibility of the work that we publish. This form provides structure for consistency and transparency in reporting. For further information on Nature Portfolio policies, see our Editorial Policies and the Editorial Policy Checklist.

Statistics

For all statistical analyses, confirm that the following items are present in the figure legend, table legend, main text, or Methods section.

n/a	Confirmed	
☐	☒	The exact sample size (*n*) for each experimental group/condition, given as a discrete number and unit of measurement
☐	☒	A statement on whether measurements were taken from distinct samples or whether the same sample was measured repeatedly
☐	☒	The statistical test(s) used AND whether they are one- or two-sided<br>*Only common tests should be described solely by name; describe more complex techniques in the Methods section.*
☒	☐	A description of all covariates tested
☐	☒	A description of any assumptions or corrections, such as tests of normality and adjustment for multiple comparisons
☐	☒	A full description of the statistical parameters including central tendency (e.g. means) or other basic estimates (e.g. regression coefficient) AND variation (e.g. standard deviation) or associated estimates of uncertainty (e.g. confidence intervals)
☐	☒	For null hypothesis testing, the test statistic (e.g. *F*, *t*, *r*) with confidence intervals, effect sizes, degrees of freedom and *P* value noted<br>*Give P values as exact values whenever suitable.*
☒	☐	For Bayesian analysis, information on the choice of priors and Markov chain Monte Carlo settings
☒	☐	For hierarchical and complex designs, identification of the appropriate level for tests and full reporting of outcomes
☐	☒	Estimates of effect sizes (e.g. Cohen's *d*, Pearson's *r*), indicating how they were calculated

Our web collection on statistics for biologists contains articles on many of the points above.

Software and code

Policy information about availability of computer code

Data collection	Ellipticity Data was acquired with a Chirascan V100 (AppliedPhotophysics) with accompanying software.
Data analysis	All Data analysis software/tools/etc are clearly mentioned in the manuscript and comprise: Python (v 3.9.5), pandas (v 2.0.3), NumPy (v 1.26.4), SciPy (v 1.13.1), matplotlib (v 3.6.2), logomaker (v 0.8), bedtools (v 2.30.0), R (v 4.1.1), regioneReloaded (v 4.3.1).<br>Custom analysis scripts are available https://doi.org/10.5281/zenodo.14217183 (2024).

For manuscripts utilizing custom algorithms or software that are central to the research but not yet described in published literature, software must be made available to editors and reviewers. We strongly encourage code deposition in a community repository (e.g. GitHub). See the Nature Portfolio guidelines for submitting code & software for further information.

Data

Policy information about availability of data

All manuscripts must include a data availability statement. This statement should provide the following information, where applicable:

- Accession codes, unique identifiers, or web links for publicly available datasets
- A description of any restrictions on data availability
- For clinical datasets or third party data, please ensure that the statement adheres to our policy

All sequencing data are publicly available on ArrayExpress (E-MTAB-14597 and E-MTAB-14598) and used motifs can be found on the github page (https://github.com/DeplanckeLab/meSMiLEseq) as well as in Supplementary Data 5 (used in figures 1, 2, 3, 4, 5 and 6). Raw imaging data (Figure 6, Supplementary Figure 7) and raw ellipticity data (figure 6b) are available in the Source Data.

Publicly available datasets that were used in the manuscript (e.g. ChIP-seq data) are listed and available in Supplementary Data 3.

Research involving human participants, their data, or biological material

Policy information about studies with human participants or human data. See also policy information about sex, gender (identity/presentation), and sexual orientation and race, ethnicity and racism.

Reporting on sex and gender	n/a
Reporting on race, ethnicity, or other socially relevant groupings	n/a
Population characteristics	n/a
Recruitment	n/a
Ethics oversight	n/a

Note that full information on the approval of the study protocol must also be provided in the manuscript.

Field-specific reporting

Please select the one below that is the best fit for your research. If you are not sure, read the appropriate sections before making your selection.

[x] Life sciences   [ ] Behavioural & social sciences   [ ] Ecological, evolutionary & environmental sciences

For a reference copy of the document with all sections, see nature.com/documents/nr-reporting-summary-flat.pdf

Life sciences study design

All studies must disclose on these points even when the disclosure is negative.

Sample size	Each sequencing run targeted at least 2 million reads per experiment, and all experiments presented in this manuscript were acquired in four batches on either Illumina MiSeq or NextSeq500 platforms. This yields a mean coverage of 166,666 reads per sample (per TF), each comprising a random region of 24 nucleotides. To assess whether this coverage sufficiently samples sequence space, we partitioned reads into kmers and show that their distribution matches the theoretically expected normal distribution of all possible kmers (Supplementary Figure 3c)
Data exclusions	Raw sequences from SMiLE-seq experiments that did not contain significantly enriched k-mers were filtered out of the analysis (also described in detail in the main and method section of the manuscript). meSMiLEseq reads were not filtered.
Replication	meSMiLE-seq experiments have been replicated over 24 independent experiments, each experiment contained a random set of 12 TFs. Detailed information can be accessed ArrayExpress (E-MTAB-14597 and E-MTAB-14598). DNA motifs showed high concordance with motifs inferred from orthogonal methods. For a selected set of TFs, the binding site was additionally proven via EMSAs (single, independent experiments).
Randomization	The samples (TFs) were allocated randomly across experiments and exposed to randomized DNA libraries across different experimental batches.
Blinding	not applicable, all data acquired with (me)SMiLE-seq were sequenced and analyzed

Reporting for specific materials, systems and methods

We require information from authors about some types of materials, experimental systems and methods used in many studies. Here, indicate whether each material, system or method listed is relevant to your study. If you are not sure if a list item applies to your research, read the appropriate section before selecting a response.

Materials & experimental systems

n/a	Involved in the study	
[ ]	[x]	Antibodies
[x]	[ ]	Eukaryotic cell lines
[x]	[ ]	Palaeontology and archaeology
[x]	[ ]	Animals and other organisms
[x]	[ ]	Clinical data
[x]	[ ]	Dual use research of concern
[x]	[ ]	Plants

Methods

n/a	Involved in the study	
[x]	[ ]	ChIP-seq
[x]	[ ]	Flow cytometry
[x]	[ ]	MRI-based neuroimaging

Antibodies

Antibodies used

-biotinylated goat anti-GFP antibody to capture TF on the microfluidic device (ab6658), Lt: GR288567-27, diluted 1:100 in PBS)
-mouse anti-Z-DNA Z22, CN: Ab00783-3.0, LN: T2248B29

Validation

-Verification and detailed protocol of the technique is described in Isakova et al. (2017), SMiLE-seq identifies binding motifs of single and dimeric transcription factors https://doi.org/10.1038/nmeth.4143.
-mouse anti-Z-DNA Z22, CN: Ab00783-3.0 as described in Meng et al. (2022), Z-DNA is remodelled by ZBTB43 in prospermatogonia to safeguard the germline genome and epigenome

Plants

Seed stocks

n/a

Novel plant genotypes

n/a

Authentication

n/a
