# Supplementary material for: Identification of methylation-sensitive human transcription factors using meSMiLE-seq
Source: Nat Commun. 2026 Aug 5;17:7771. doi: 10.1038/s41467-026-71387-y (PMC13444138; doi:10.1038/s41467-026-71387-y)
Supplement: Supplementary file 5 [file 41467_2026_71387_MOESM4_ESM.pdf]

Description of Additional Supplementary Files

Supplementary Data 1: Overview of TFs that were investigated in this study.

Supplementary Data 2: Re-curated and unique TFs differing from collaborator's study.

Supplementary Data 3: Orthogonal datasets used in this study.

Supplementary Data 4: Overview of all meSMiLE-seq-derived DNA binding motifs and scatterplots.

Supplementary Data 5: Fine grained enrichment analysis for uLib and mLib and TF classification based on affinity towards methylated DNA.

Supplementary Data 6: Methylation levels of genomic TFBS.

Supplementary Data 7: DNA libraries and primers.
