# Supplementary material for: Identification of methylation-sensitive human transcription factors using meSMiLE-seq
Source: Nat Commun. 2026 Aug 5;17:7771. doi: 10.1038/s41467-026-71387-y (PMC13444138; doi:10.1038/s41467-026-71387-y)

Supplementary Data 4. Overview of all meSMiLE-seq-derived DNA binding motifs and scatterplots

- ⚠ = **weak enrichment**
- ○ = **without CG**
- ○ (red) = **with CG**

C2H2 ZNF

PRDM5 DBD: weak methyl minus

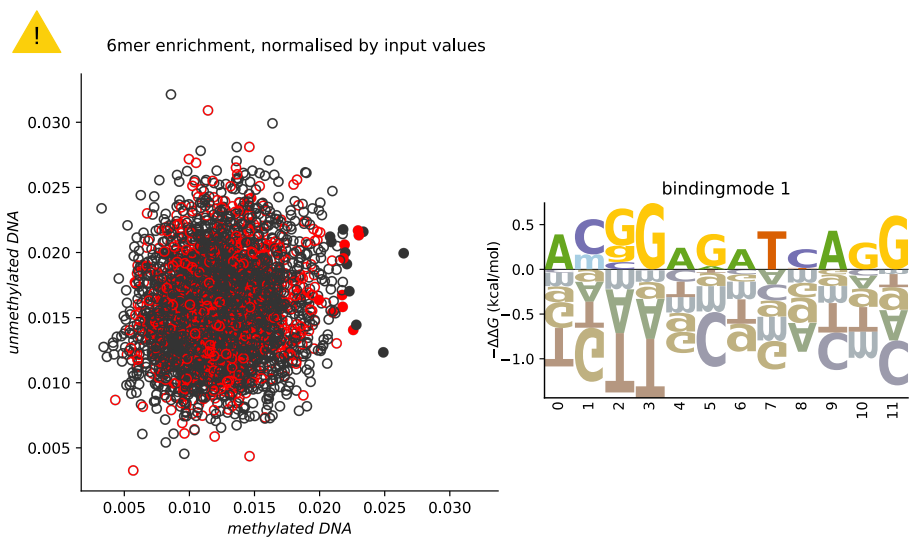

PRDM10 DBD: little effect

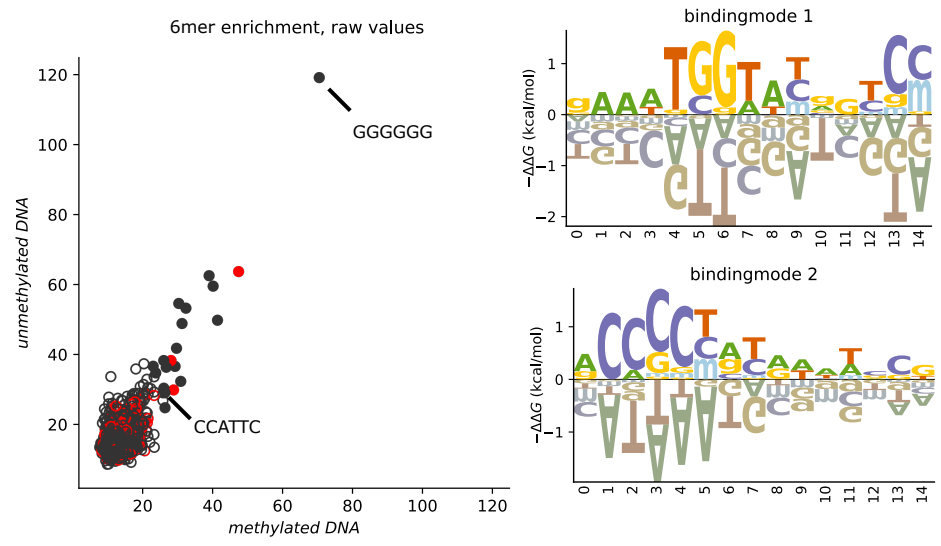

PRDM13 DBD: weak methyl plus

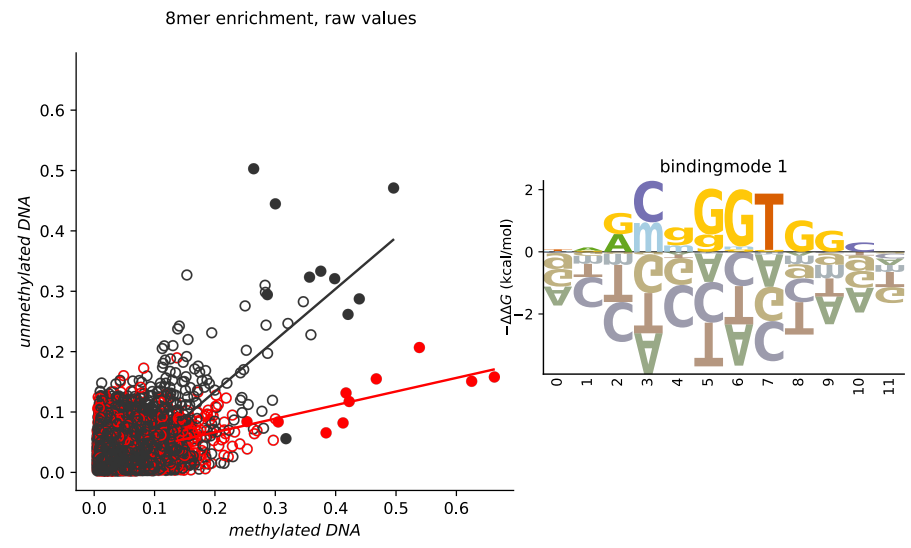

ZBTB5 DBD: no CG

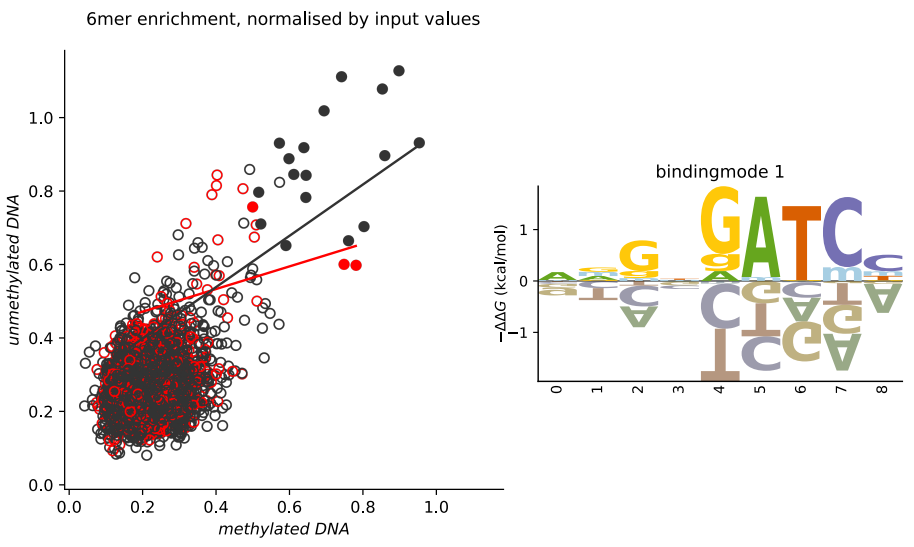

ZBTB46 DBD: little effect

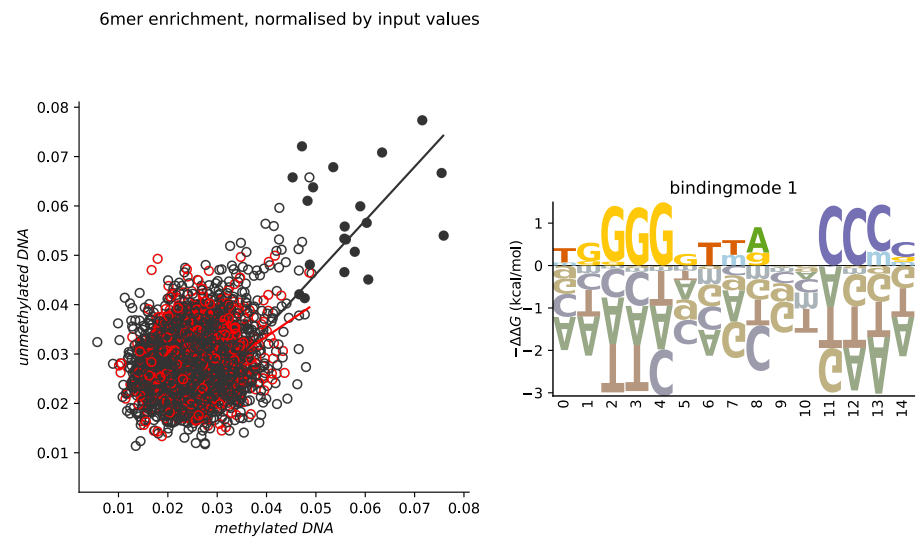

ZFP1 FL: no CG

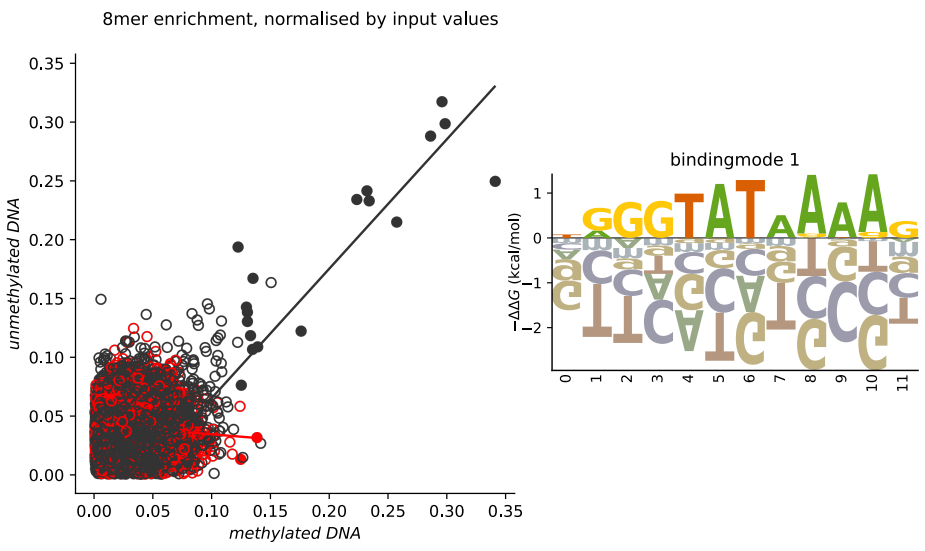

ZFP14 FL: little effect

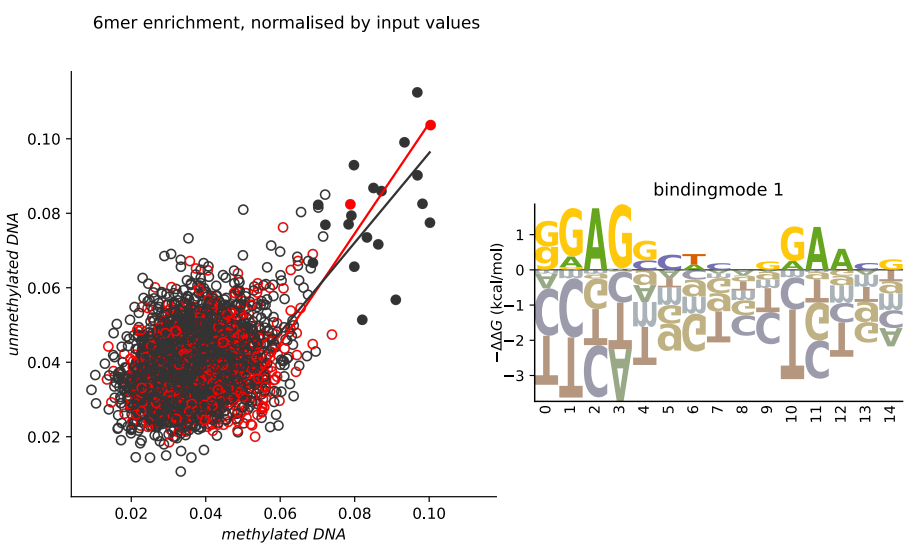

ZKSCAN4 DBD: weak methyl plus

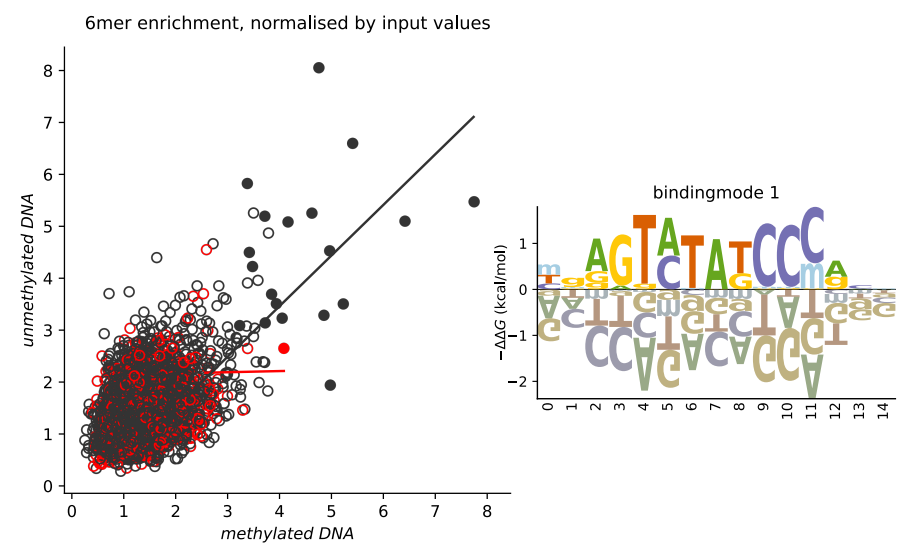

ZNF8 FL: weak methyl minus

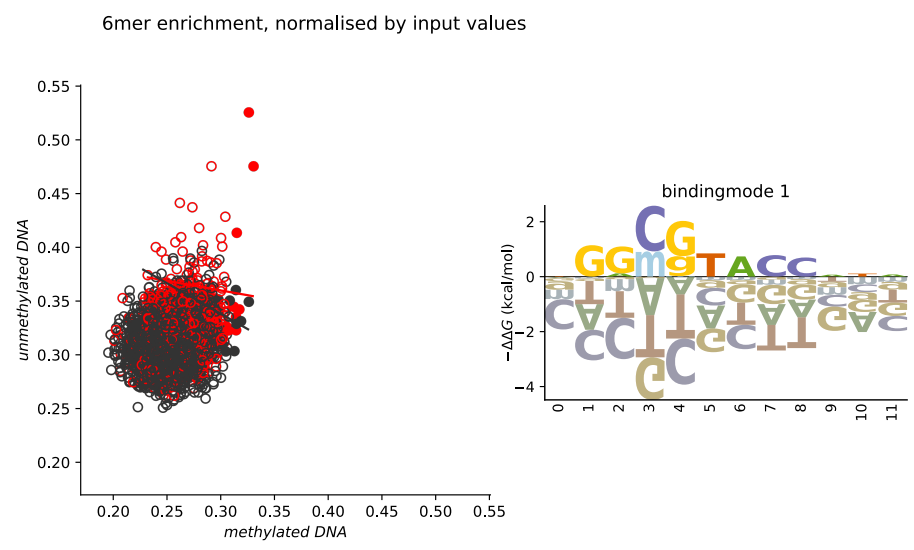

ZNF18 FL: weak methyl plus

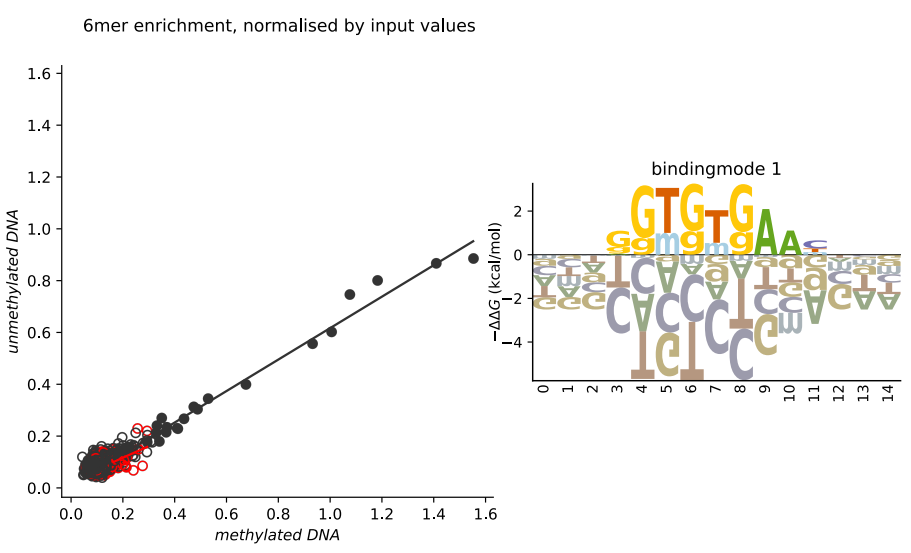

ZNF23 FL: weak methyl plus

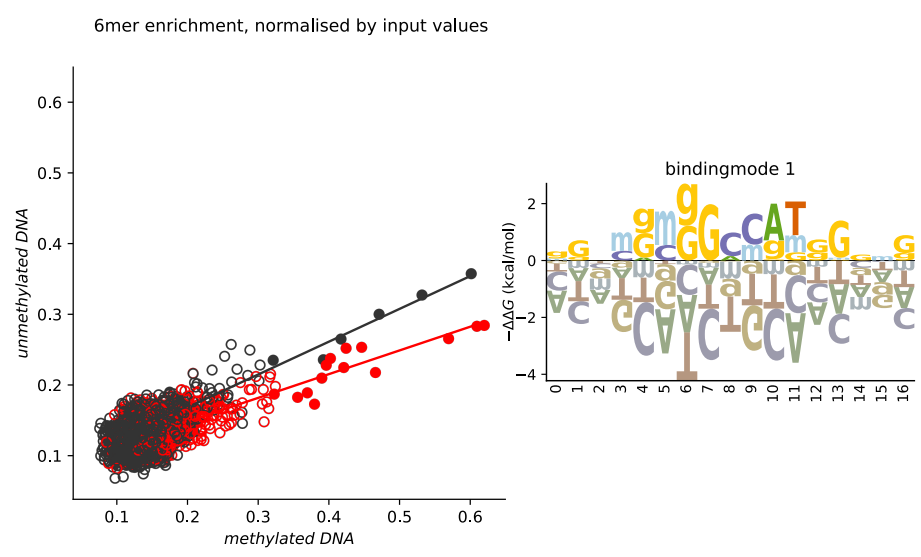

ZNF75D FL: no CG

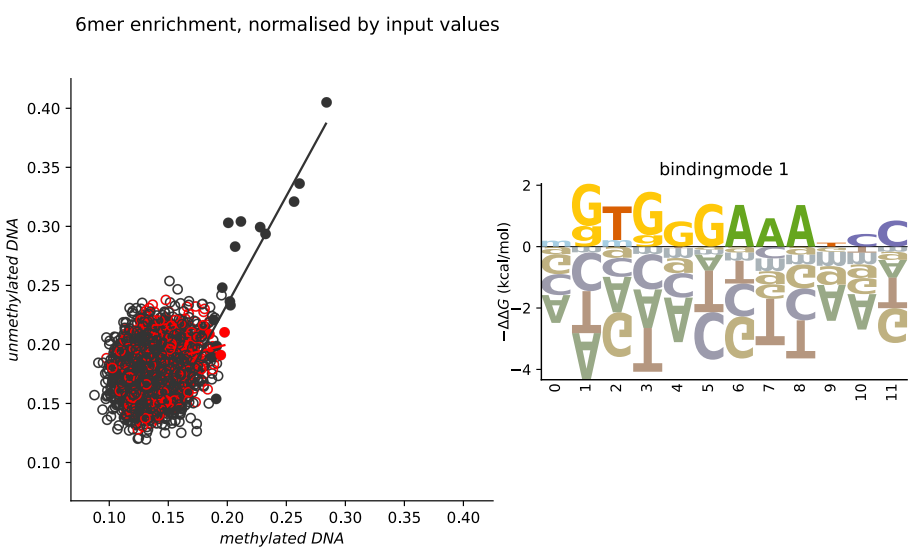

ZNF131 FL: methyl minus

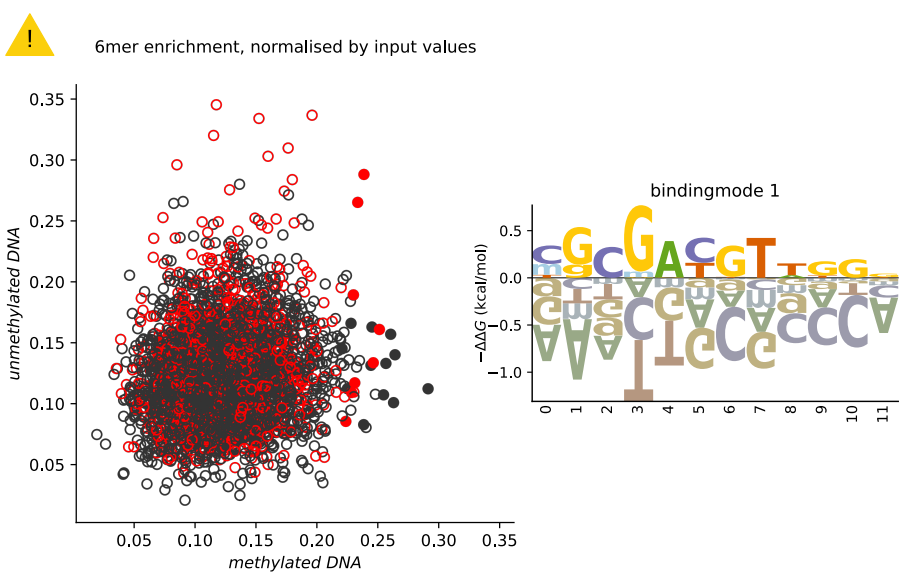

ZNF133 FL: weak methyl plus

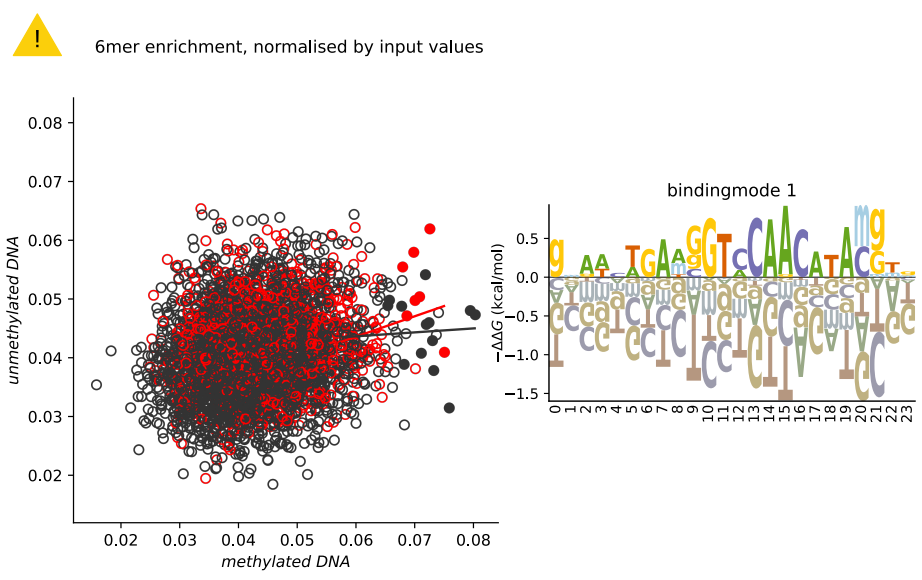

ZNF248 FL: little effect

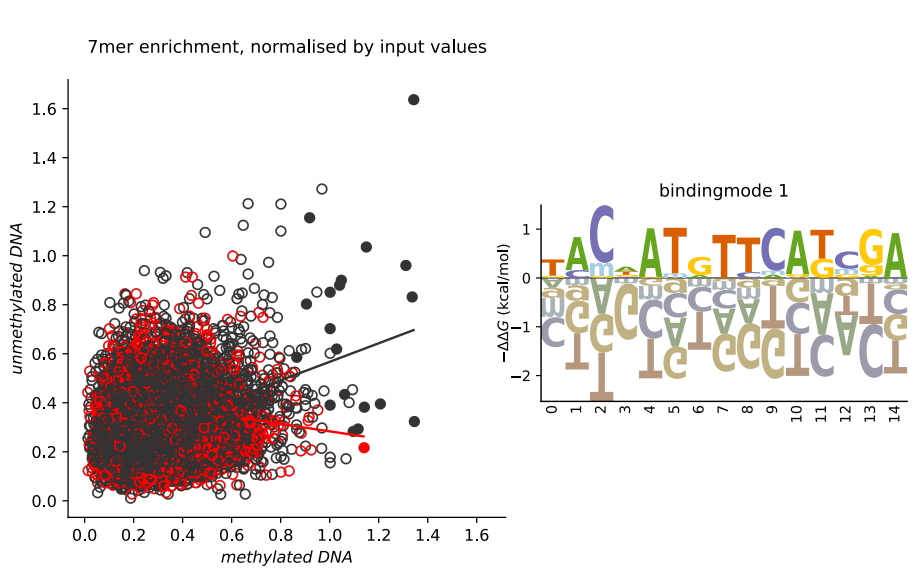

C2H2 ZNF

ZNF263 FL: little effect

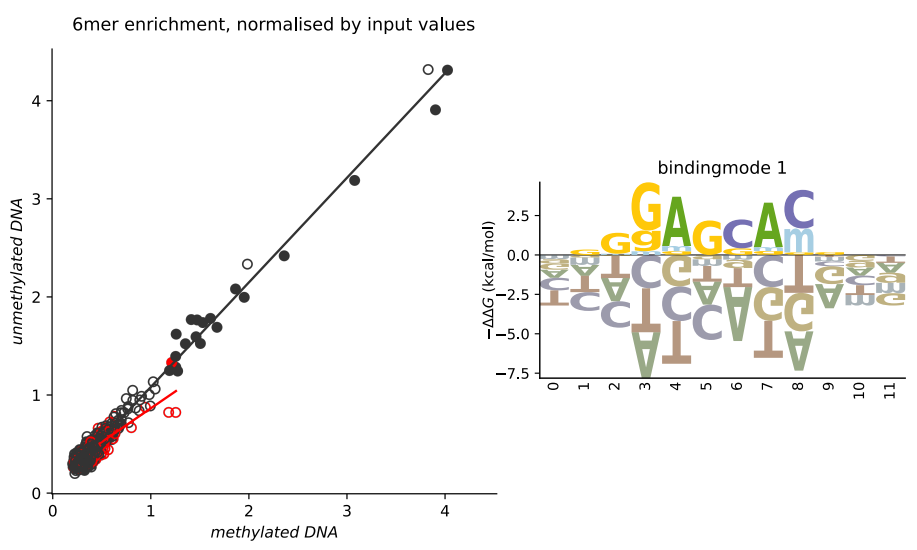

ZNF333 FL: little effect

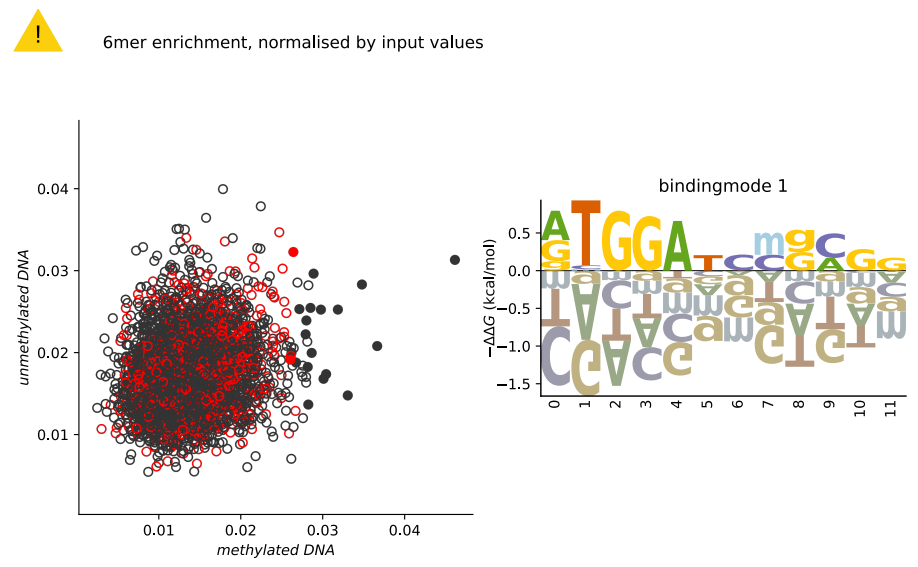

ZNF367 FL: no CG

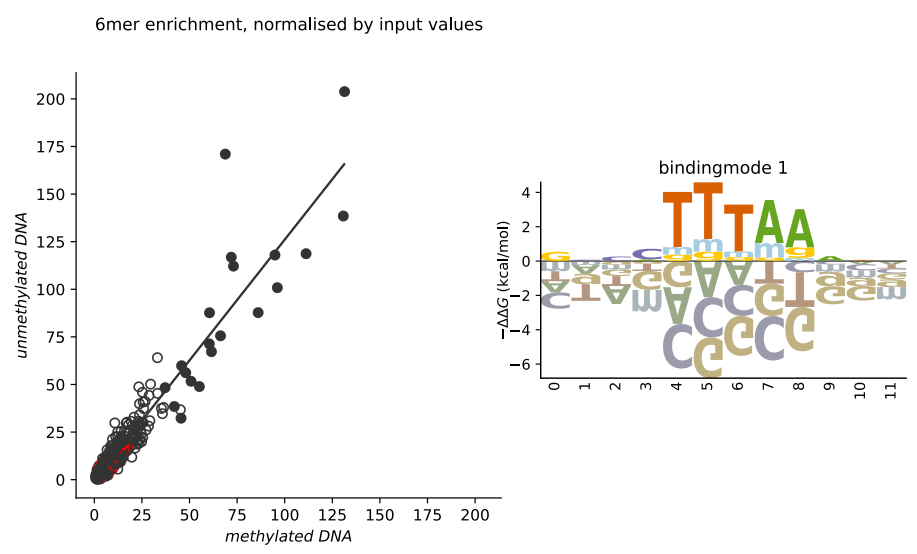

ZNF395 DBD: methyl minus

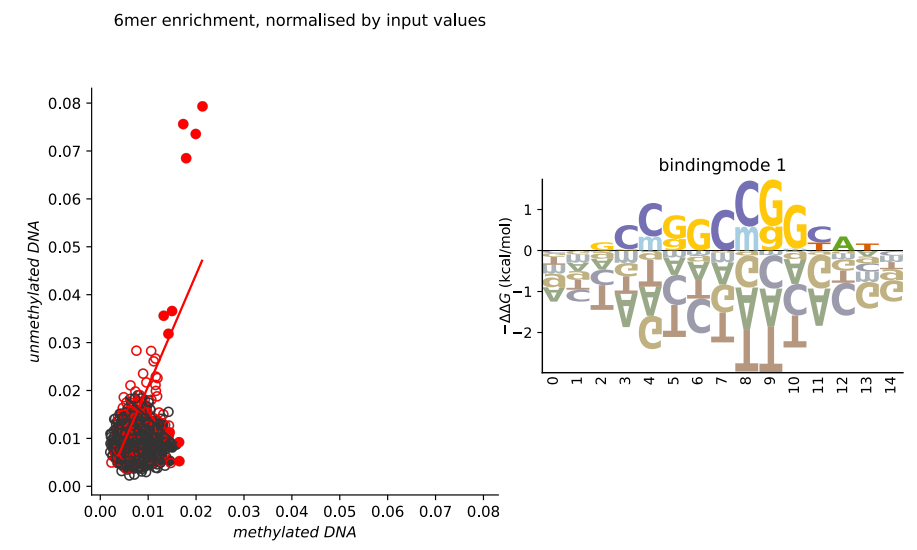

ZNF445 FL: methyl plus

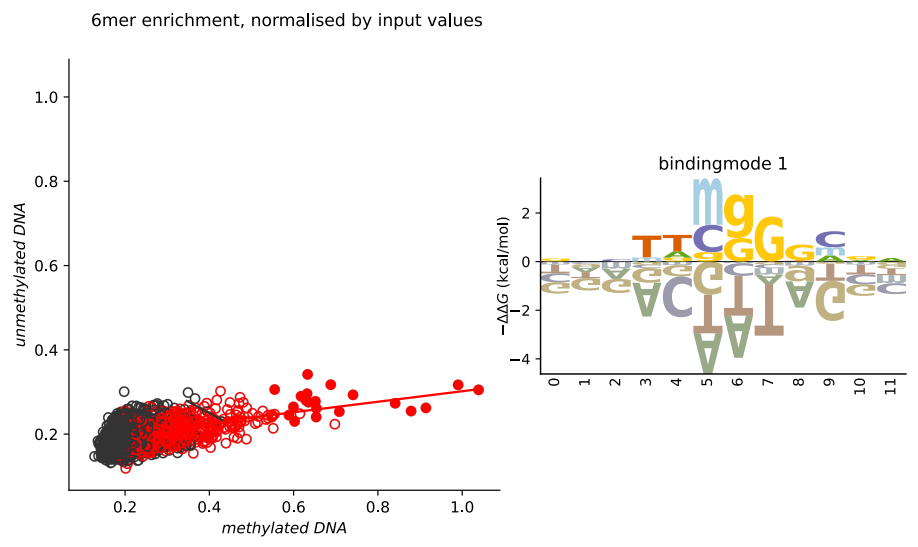

ZNF468 FL: no CG

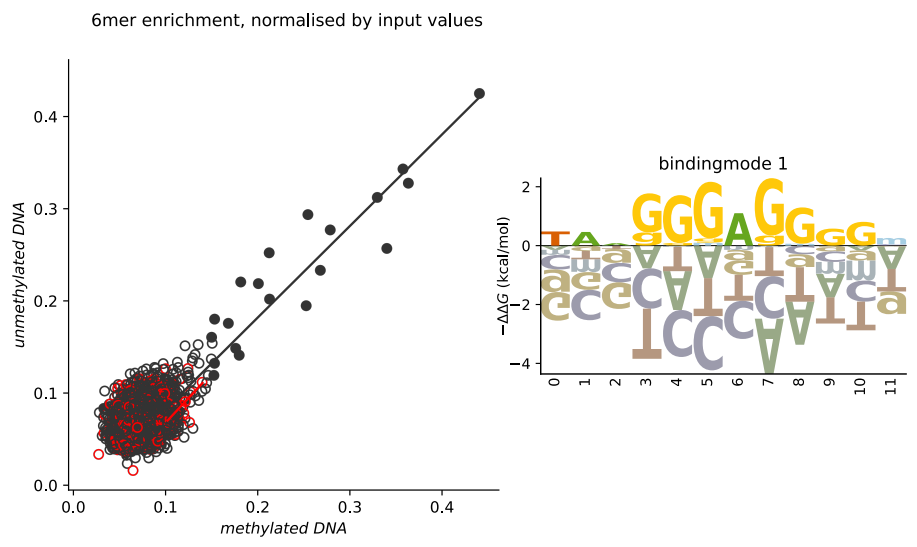

ZNF503 DBD: weak methyl plus

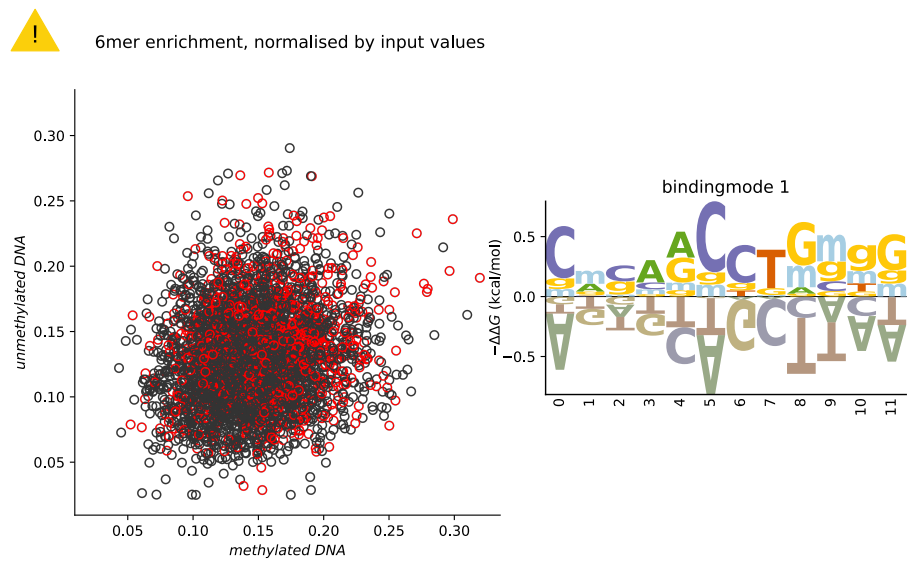

ZNF518B DBD: little effect/weak methyl plus

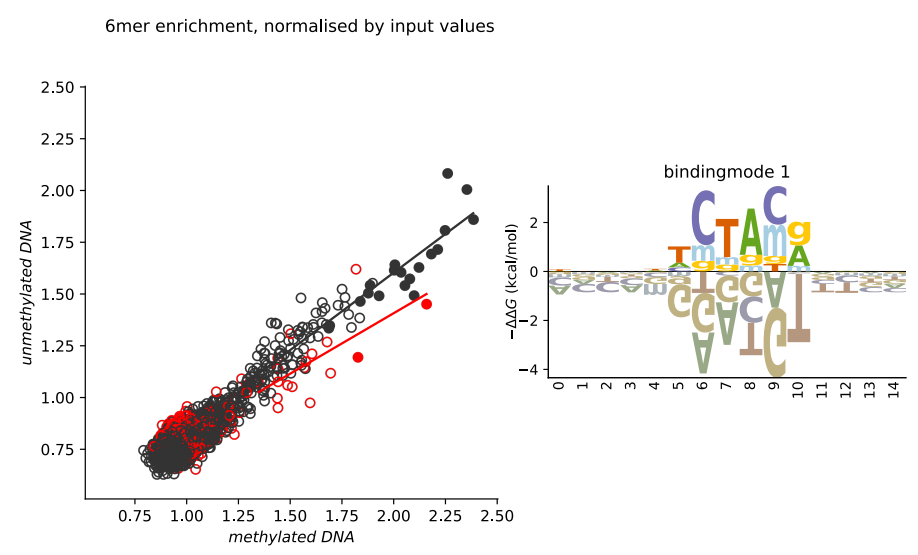

ZNF614 FL: weak methyl minus

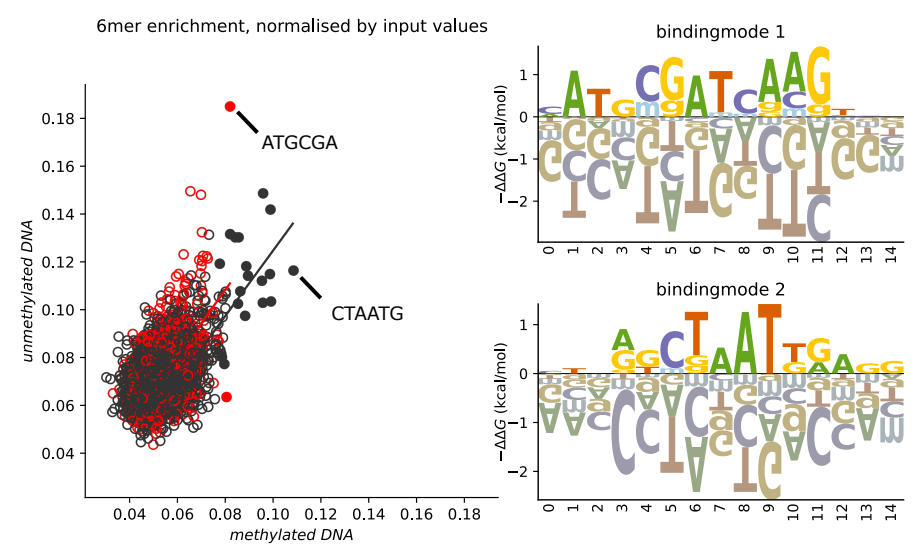

ZNF648 DBD: weak methyl plus

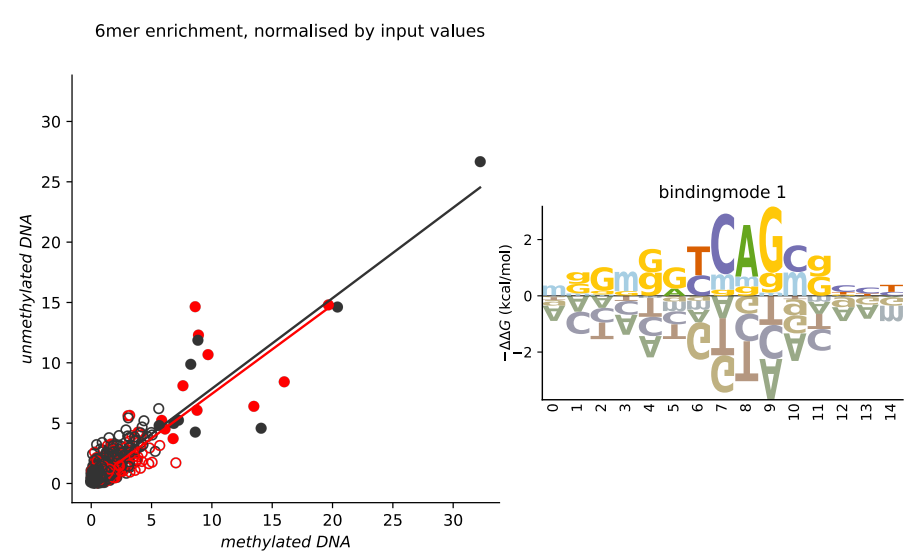

ZNF680 FL: little effect

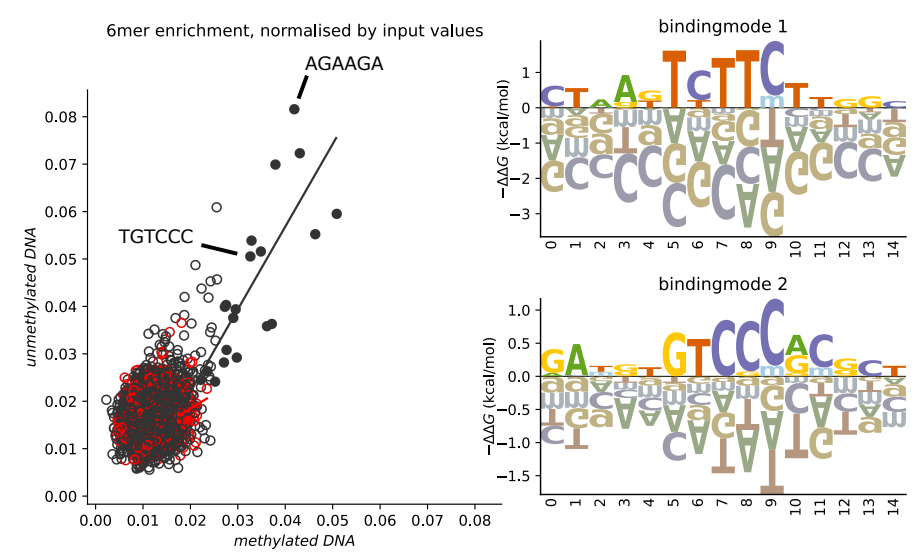

ZNF695 FL: little effect

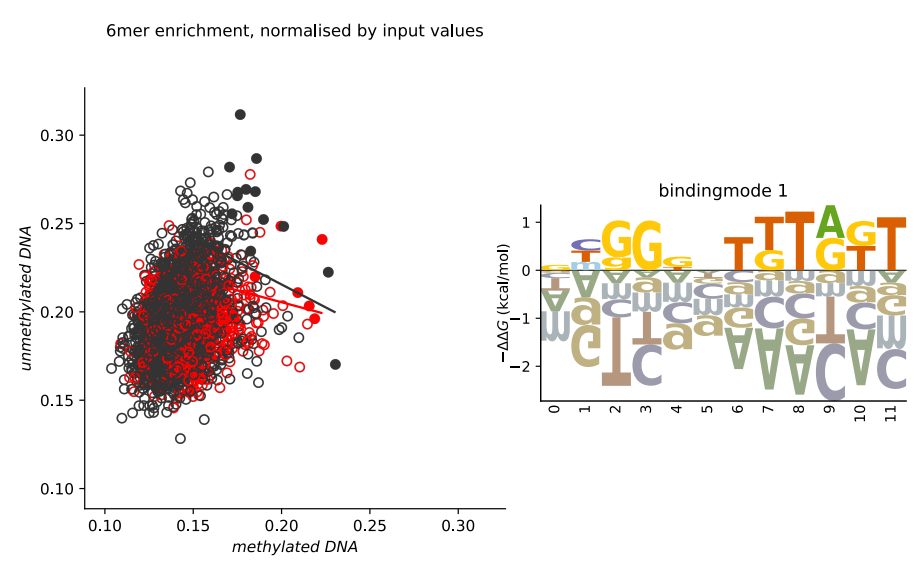

ZNF716 FL: methyl plus

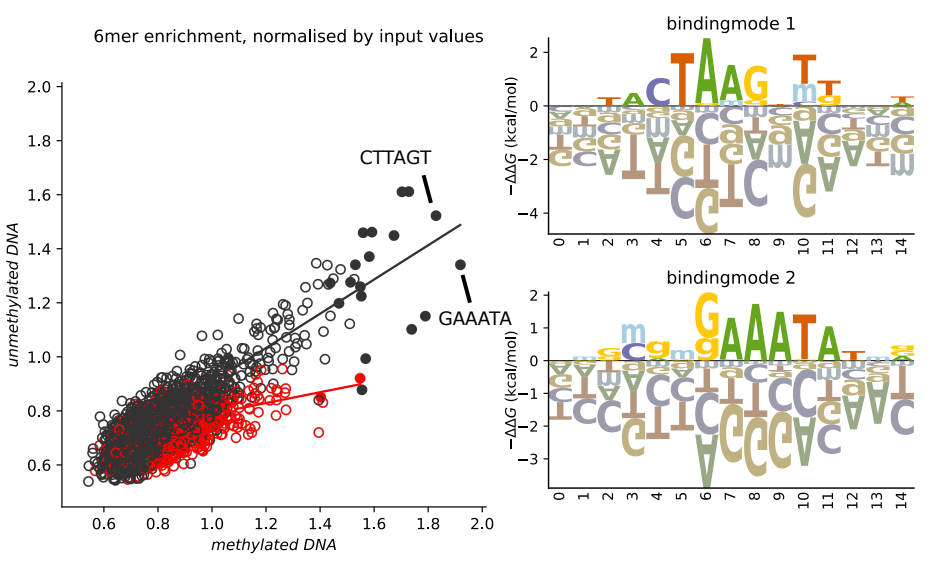

ZNF777 FL: methyl minus

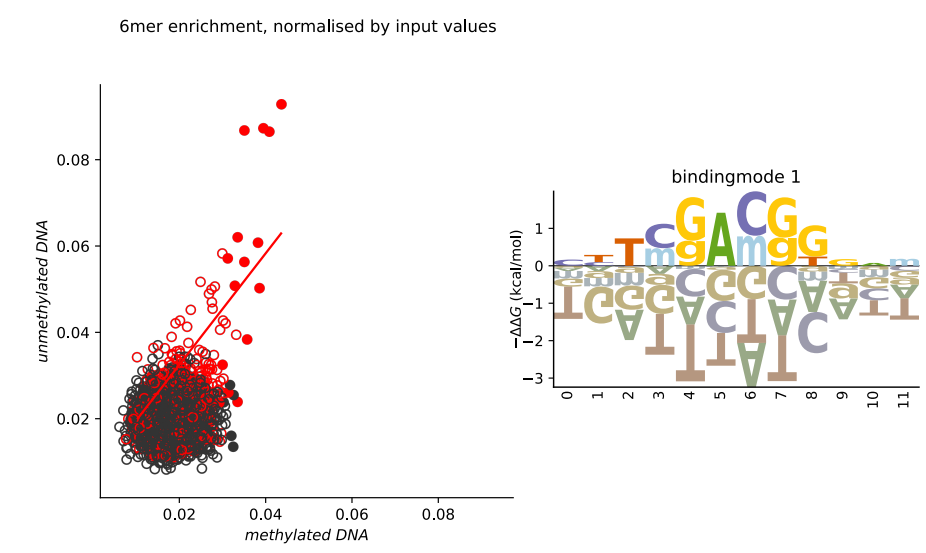

ZNF793 FL: little effect

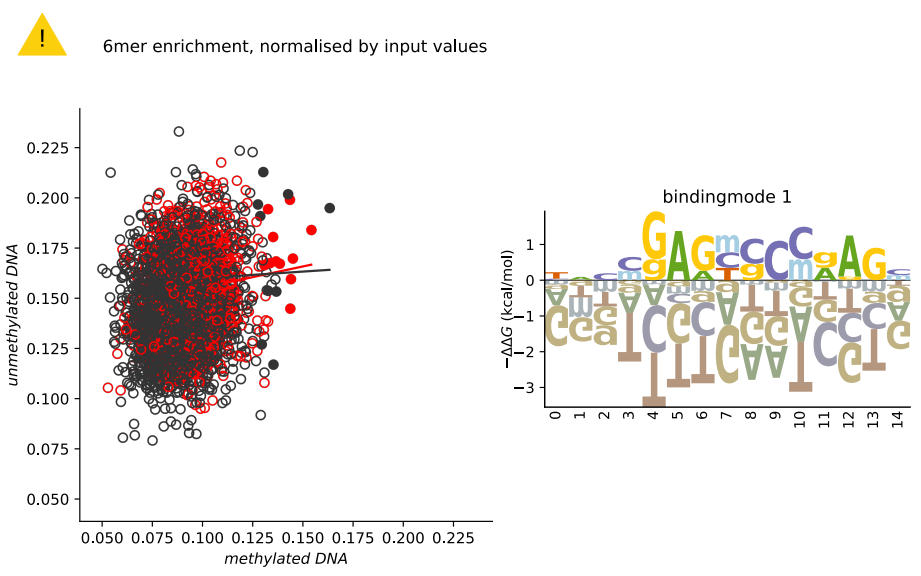

C2H2 ZNF

ZNF831 DBD: little effect

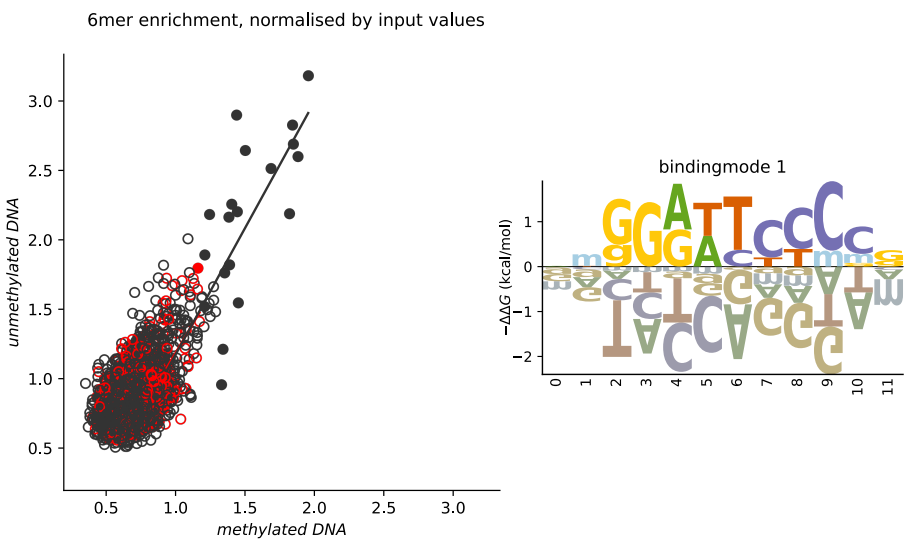

ZNF891 FL: little effect

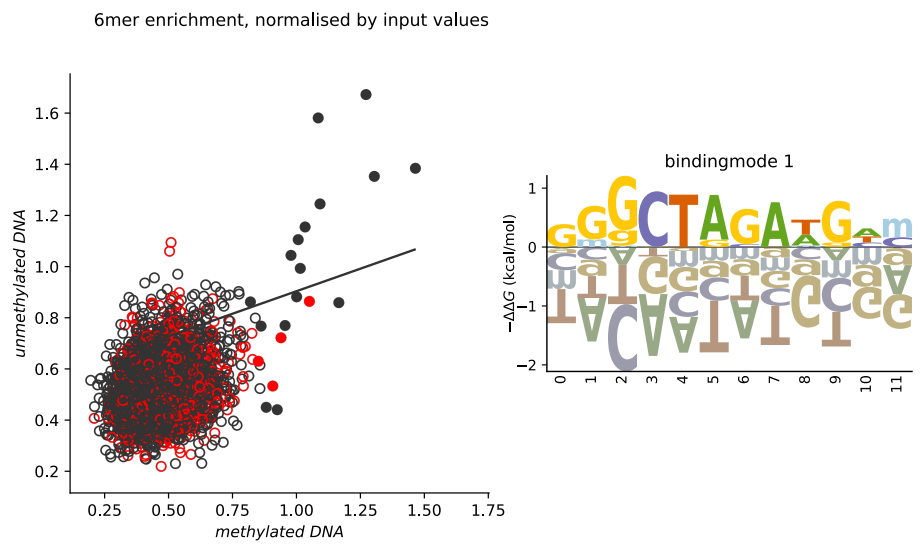

ZXDB DBD: weak methyl minus

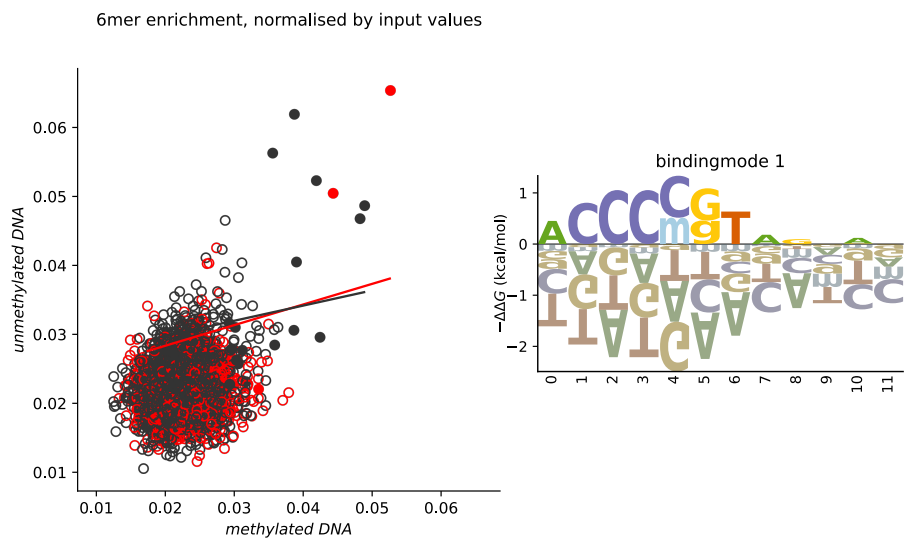

Homeodomain, C2H2 ZNF

ZHX2 FL: methyl plus

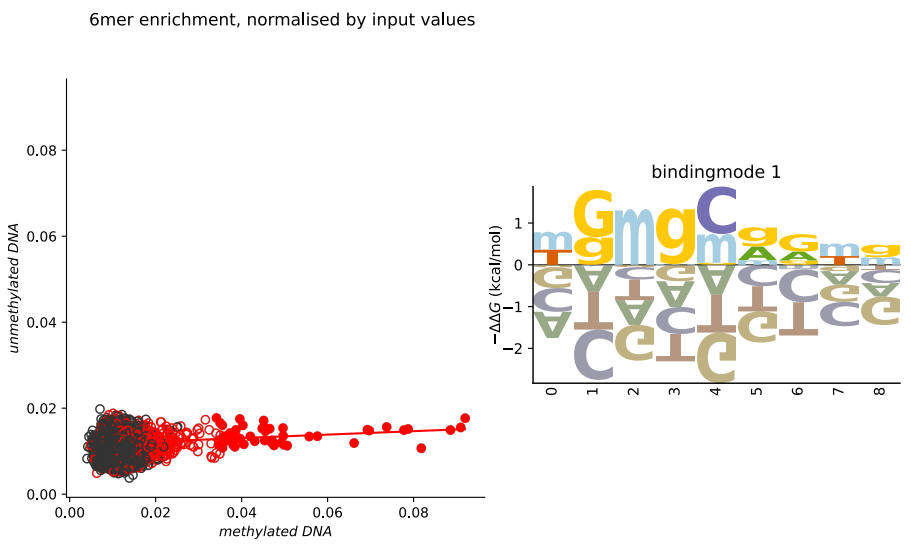

BED ZF

FAM200B DBD: methyl minus

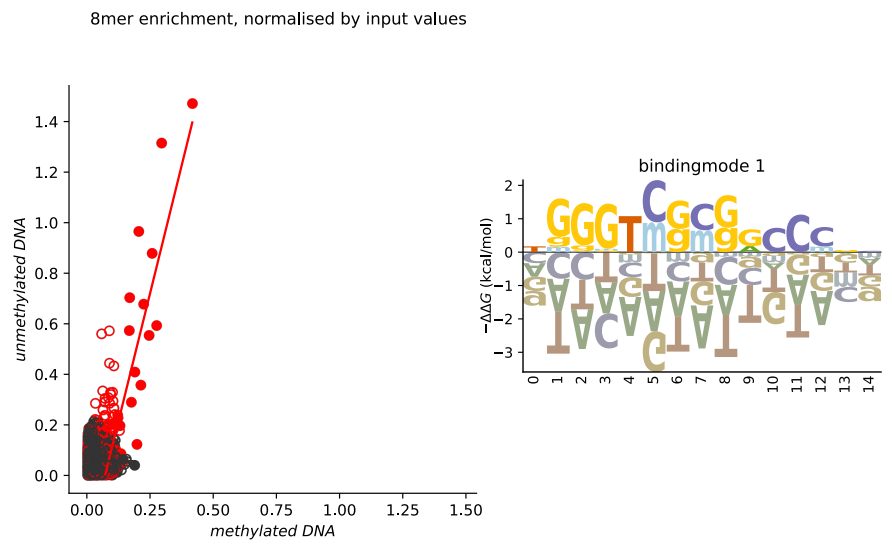

ZBED4 DBD: weak methyl minus

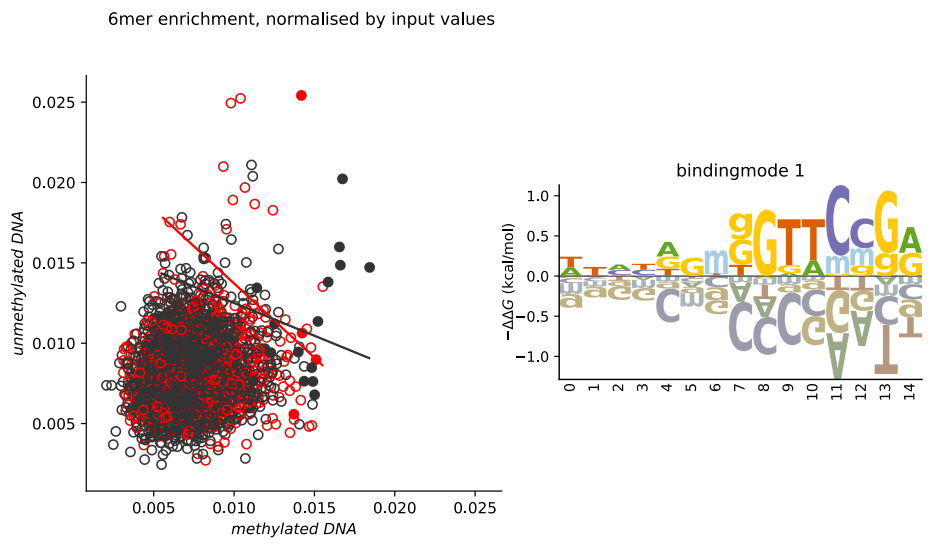

unknown

TET2 FL: methyl plus

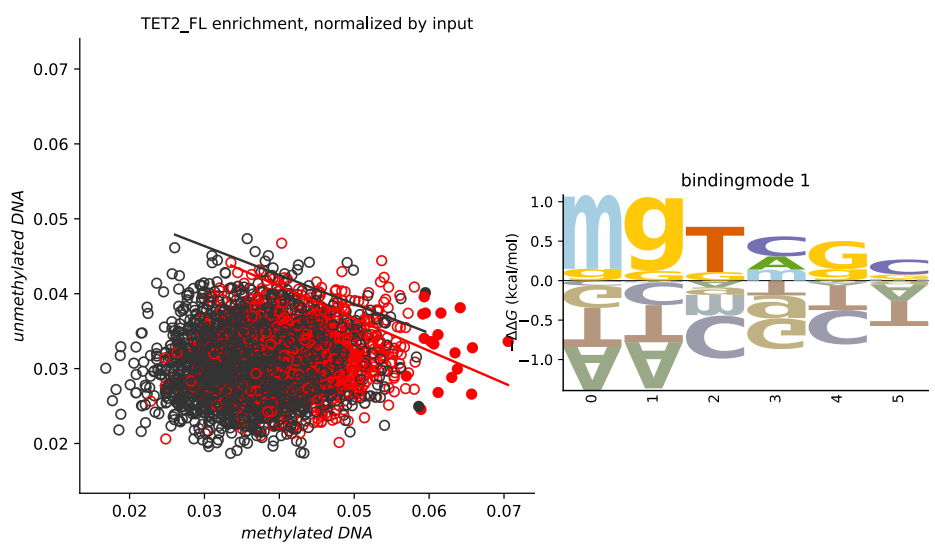

bHLH

USF3 DBD: methyl minus

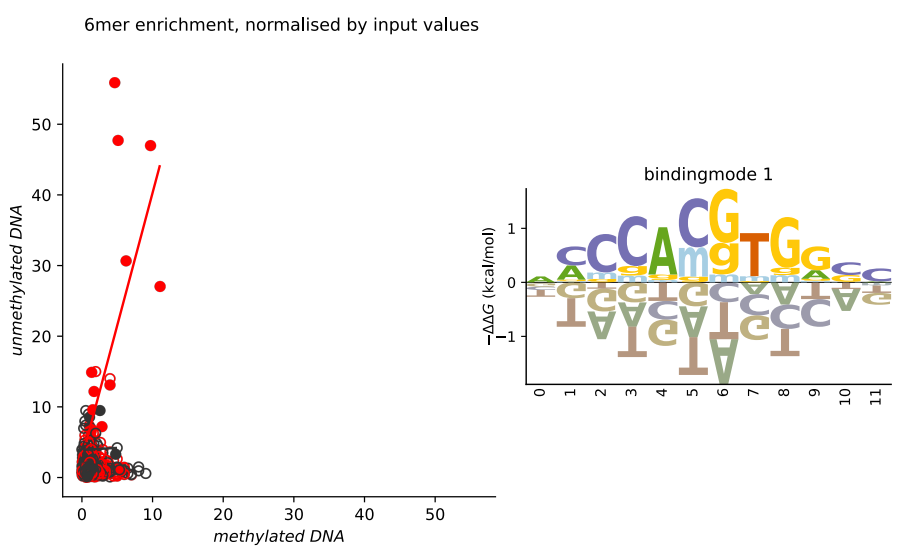

CENPB

TIGD4 FL: weak methyl minus

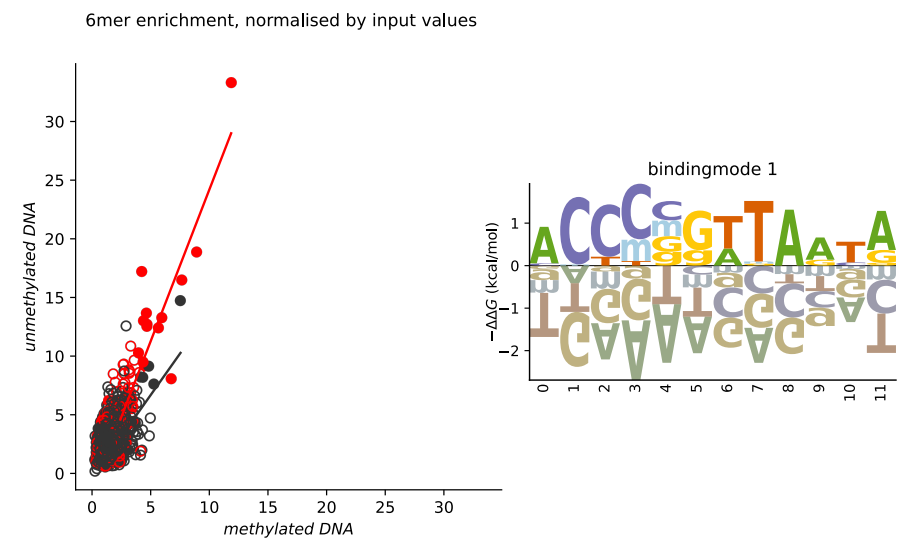

TIGD5 DBD: methyl minus

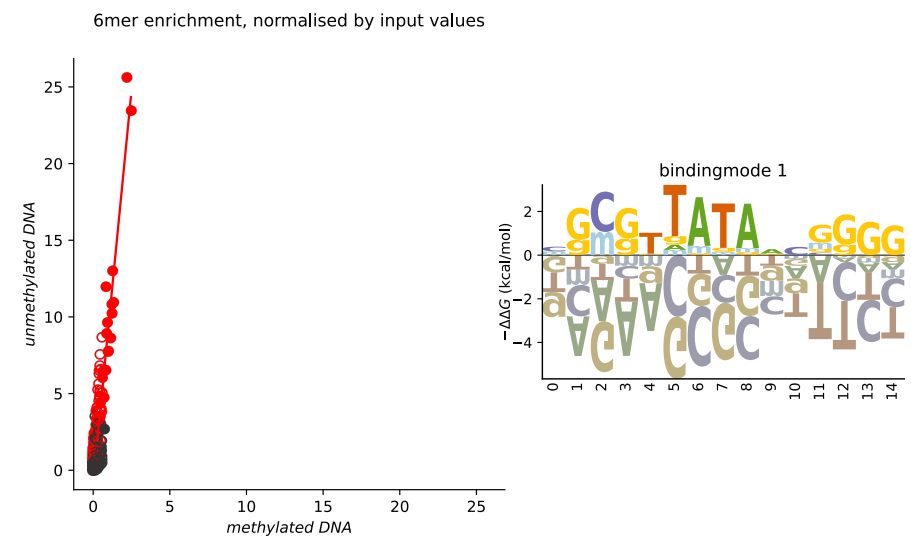

CG-1

CAMTA1 DBD: little effect

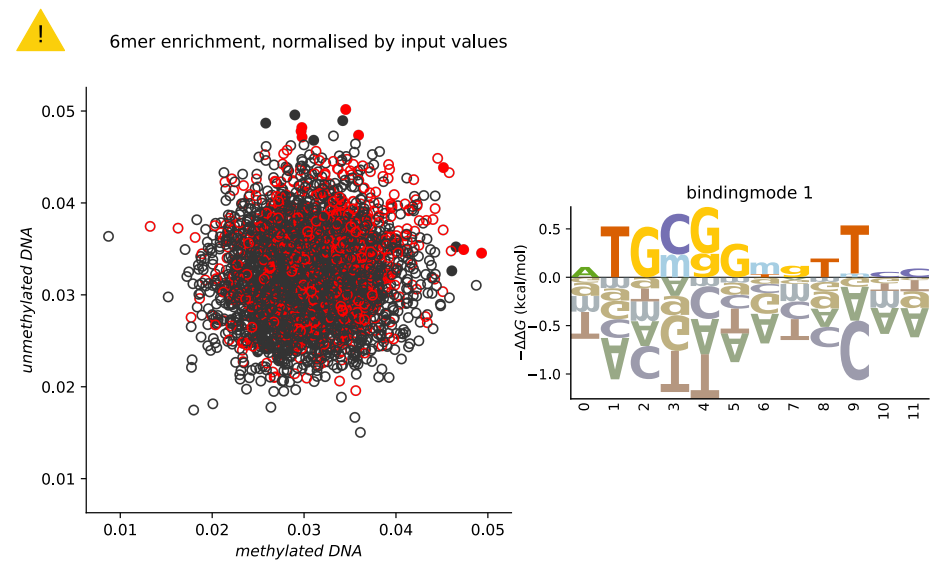

CXXC ZF/MBD

CXXC4 FL: methyl minus

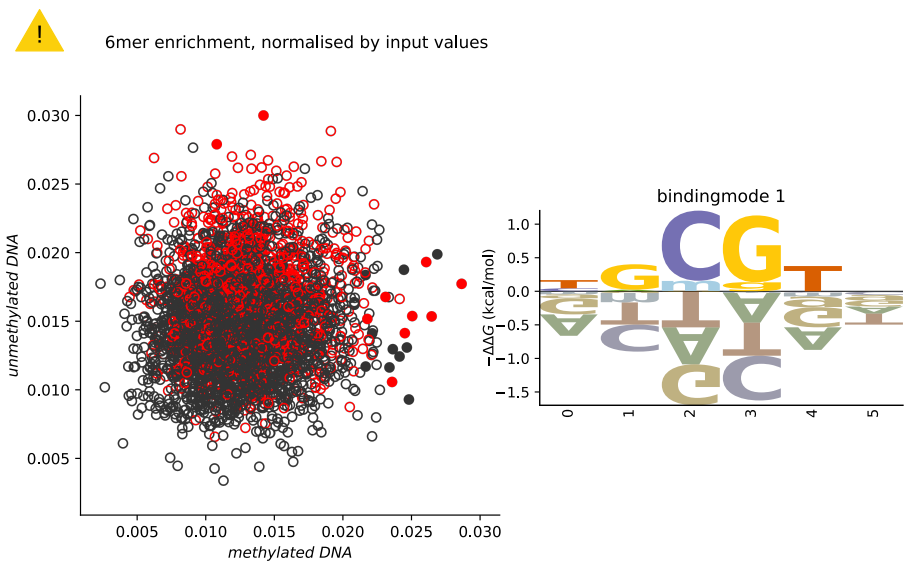

MBD1 DBD: methyl plus

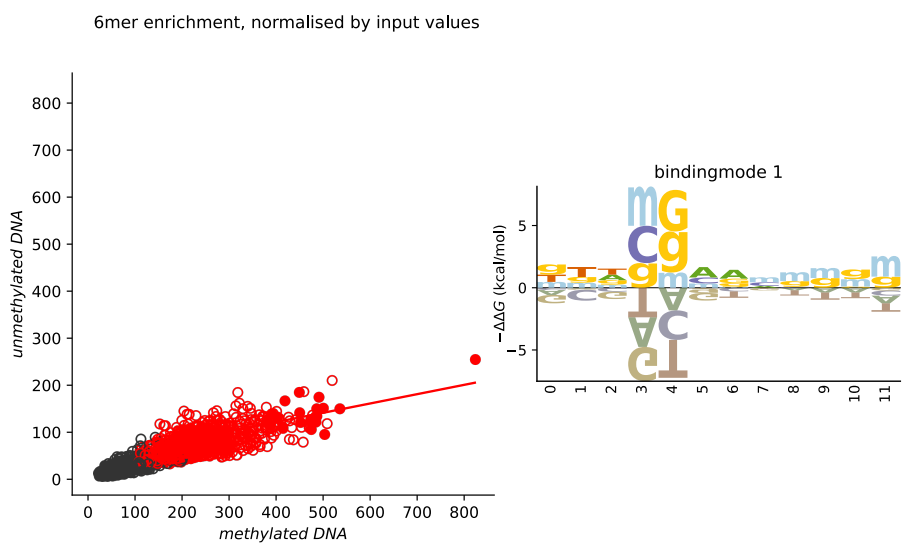

MADF

MSANTD1 FL: no CG

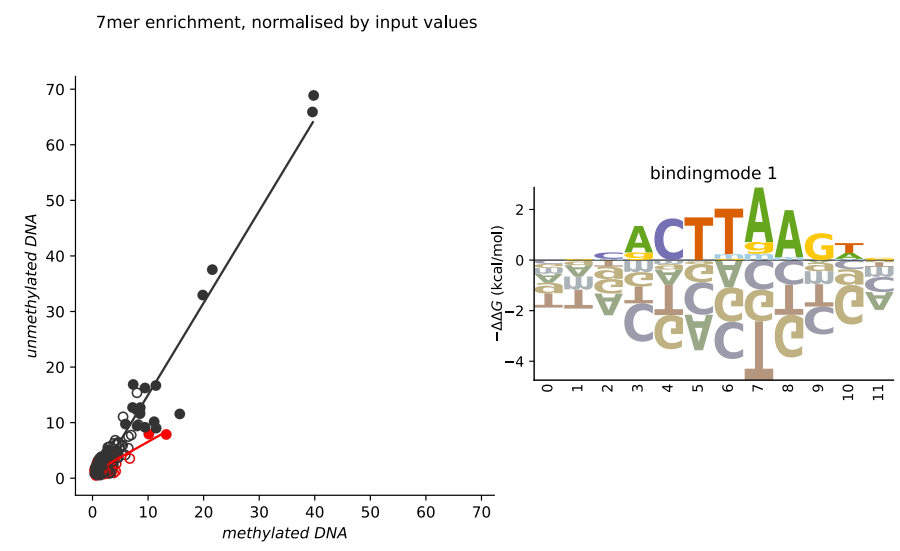

Homeodomain/POU

TPRX1 DBD: no CG

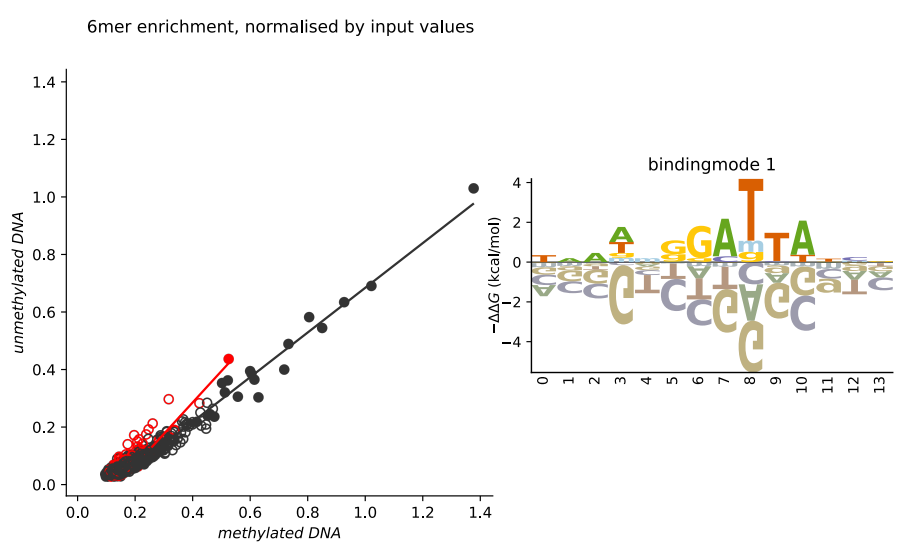

POU5F1 FL: methyl plus

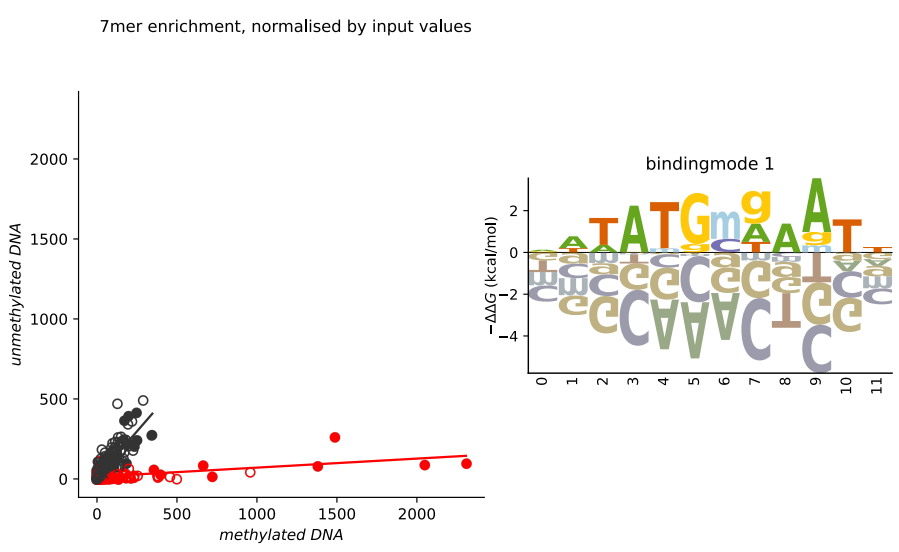

Myb/SANT

TTF1 DBD: little effect

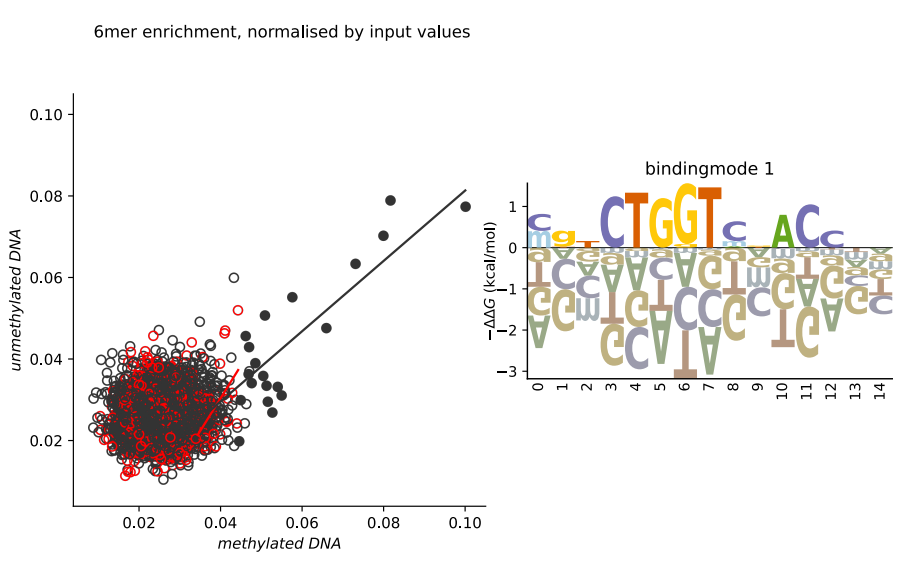

RFX

RFX5 FL: methyl plus

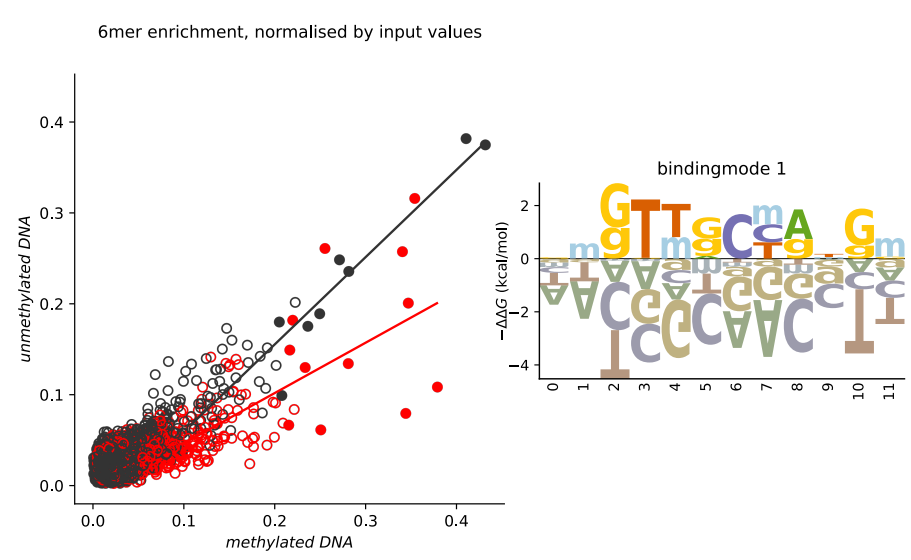

Supplement: Supplementary file 6 [file 41467_2026_71387_MOESM8_ESM.pdf]
